# High probability of yield gain through conservation agriculture in dry regions for major staple crops

**DOI:** 10.1038/s41598-021-82375-1

**Published:** 2021-02-08

**Authors:** Yang Su, Benoit Gabrielle, Damien Beillouin, David Makowski

**Affiliations:** 1 UMR ECOSYS, INRAE AgroParisTech, Université Paris-Saclay, 78850 Thiverval-Grignon, France; 2 UMR Agronomie, INRAE AgroParisTech, Université Paris-Saclay, 78850 Thiverval-Grignon, France; 3 Applied Mathematics and Computer Science (MIA 518), INRAE AgroParisTech, Université Paris-Saclay, 75005 Paris, France; 4 CIRAD, UPR HortSys, 34398 Montpellier, France; 5 HortSys, Univ Montpellier, CIRAD, Montpellier, France

**Keywords:** Climate-change mitigation, Climate-change adaptation, Agroecology

## Abstract

Conservation agriculture (CA) has been promoted to mitigate climate change, reduce soil erosion, and provide a variety of ecosystem services. Yet, its impacts on crop yields remains controversial. To gain further insight, we mapped the probability of yield gain when switching from conventional tillage systems (CT) to CA worldwide. Relative yield changes were estimated with machine learning algorithms trained by 4403 paired yield observations on 8 crop species extracted from 413 publications. CA has better productive performance than no-till system (NT), and it stands a more than 50% chance to outperform CT in dryer regions of the world, especially with proper agricultural management practices. Residue retention has the largest positive impact on CA productivity comparing to other management practices. The variations in the productivity of CA and NT across geographical and climatical regions were illustrated on global maps. CA appears as a sustainable agricultural practice if targeted at specific climatic regions and crop species.

## Introduction

Conservation agriculture was originally designed to decrease soil erosion while sustaining crop productivity in the long run^1^. It consists of three principles: preserving a permanent soil cover, minimizing soil disturbance (going as far as NT), and diversifying crop species^2^. Under certain environmental conditions, CA system can provide positive environmental externalities such as increased biodiversity, enhanced carbon sequestration and improved soil quality through an increase in soil organic matter^3,4^, and is expected to enhance soil fertility, soil structure and water retention properties over time^5–7^, thereby CA could increase crop yields in particularly in regions experiencing water scarcity^8^. Nonetheless, its impacts on crop yield remains controversial. It is also reported that CA may lead to a yield reduction^1,9^ especially when the principles of conservation agriculture are only partially applied (e.g. in NT system without soil cover or without rotation)^1,9,10^. Further analyses were conducted to reveal how the productive performance of CA and NT system varies as the function of several interacting factors such as agricultural management practices^1,9,11^, soil characteristics^11^, crop species^1,12^ and climatic conditions^1,9,11,13^. However, the dataset used in those analyses only provide no or limited information on soil characteristics, climate variables, and management practices. In particular, information on fertilizer inputs, weed and pest control, and intra- and inter- annual climatic variability were frequently missing. To date, a comprehensive synthesis of the productivity of CA and NT system at the global scale, including multiple crops, a wider range of climatic parameters on yields, and a map showing the local productivity of CA and NT vs. CT at the global scale is still lacking.

In this paper, we compared the productive performance of CA and NT vs. CT under different climate conditions and different agricultural managements based on a new global dataset^14,15^. This dataset contains yield comparisons of NT vs. CT, and CA vs. CT, where CA was defined as NT with crop rotation and soil cover based on FAO’s definition^16^. In contrast with previous papers, we used here a probabilistic approach to analyse the dataset. Machine learning models^17,18^ were built to estimate the probability that CA (and NT) outperforms CT, and to compute plausible ranges of relative yield change when shifting from CT to CA (or NT) systems for eight major crops, i.e. spring barley, cotton, maize, rice, sorghum, soybean, sunflower, and winter wheat. Unlike previous studies, we included a wider range of climate drivers (with their inter- and intra-annual variabilities) in the models predicting the impact of CA and NT on crop yields, rather than relying on aridity indices or broad climate zones. This provided further insight into the effects of climate on the comparison with conventional agricultural systems.

### Data collection and analysis

A systematic literature review was performed in February of 2020 (see Supplementary S1). We collected the papers cited in above mentioned meta-analyses^1,9,11^, supplemented them by the most recently published experimental studies. The yield data of NT and CT, details of experimental site and agricultural management practices were extracted from these papers, with a broader set of climatic parameters from external databases. In the end, 4403 paired yield comparisons between NT (or CA when NT is implemented with soil cover, and crop rotation) and CT were collected from 413 papers, along with the information of crop types, years and locations of the experiments, and the detailed agricultural management practices such as crop irrigation, fertilization, the control of weeds and pests, crop rotation, the management of crop residue and soil cover. Additional data were extracted from several external databases, including crop growing season^19,20^, soil texture^21^ and climate factors such as precipitation balance (precipitation^22^—potential evapotranspiration^23,24^), minimum temperature^25^, average temperature^22^, maximum temperature^25^ throughout the growing season in a particular year. The observations covered 50 countries (see Supplementary S2) and 8 crops. Pairs of yield values were used to compute the yield ratios of CA (or NT) to CT $\left(\frac{Yiel{d}_{CA (or NT)}}{Yiel{d}_{CT}}\right)$ and the relative yield change ratios $\left(\frac{Yiel{d}_{CA (or NT)} - Yiel{d}_{CT}}{Yiel{d}_{CT}}\right)$.

Machine learning models, namely random forest and quantile regression forest were developed to analyse the database. The inputs of both models were the climatic conditions throughout growing season, crop type, soil texture, and the agricultural management practices. The output of the random forest model was the probability yield gain of CA (or NT) vs. CT (yield ratio > 1). The performance of this model was assessed by estimating the area under the ROC curve by leave-one-out cross validation (LOOCV) (AUC = 0.79, see Supplementary S3). The output of quantile regression forest model was the 1st and 3rd quartile of relative yield change ratios, corresponding to levels of losses and gains achieved in 25% and 75% of the cases. The performance of this model was assessed using a specific LOOCV procedure to check that the proportion of yield ratio in the observation within the predicted intervals defined by the two quartiles (25th and 75th quantiles) was close to 50% (51.3%, see Supplementary S4)^26^.

To predict the global performance of CA (and NT) vs. CT, the two trained machine learning models were supplemented with global climate data (the average of 1981–2010), agricultural management practices (NT: without crop rotation and without soil cover, CA: with crop rotation and with soil cover, CT: without crop rotation and without soil cover), the masks of crop presence^27^, irrigation^27^ and soil texture^21^. Details of model setting are available in Supplementary S5. Model results were then projected on map.

## Results

**Figure 1:**
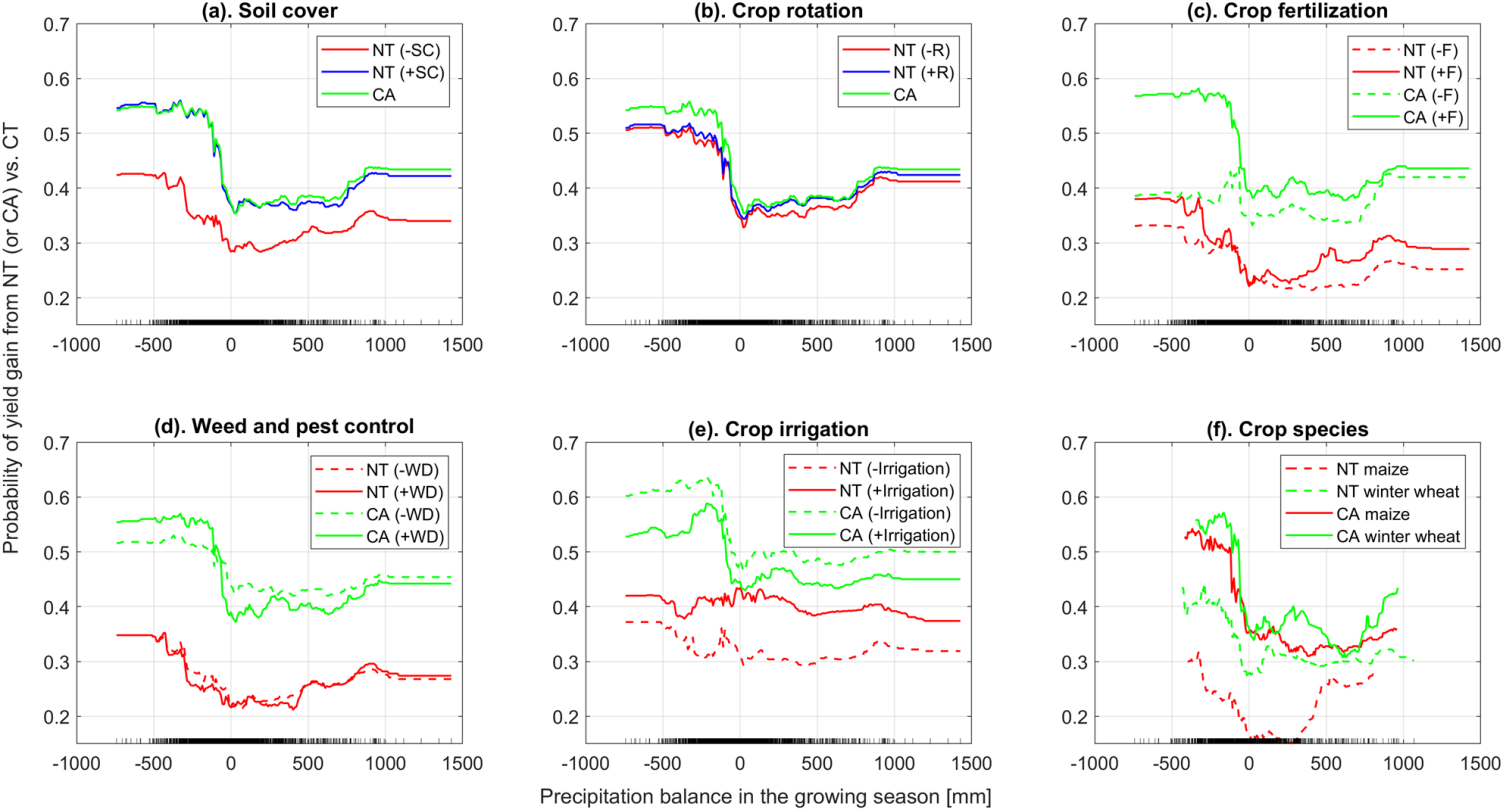
Impact of crop management practices on CA performance, shown in 1-dimension partial dependence plot of the probability of yield gain as a function of precipitation balance (mm). The tick marks on the x-axis show the distributions of observations of precipitation balance in the dataset. ± SC indicates NT with/without soil cover. ± R indicates NT with/without rotation. ± F indicates NT or CA and CT with/without fertilization. ± WD indicates NT or CA and CT with/without weed and pest control. Plot **a** compares the productive performance of CA, NT+SC, and NT-SC. Plot **b** compares the productive performance of CA, NT+R, and NT-R. Plot **c** compares the productive performance of CA+F, CA-F, NT+F, and NT-F. Plot **d** compares the productive performance of CA+WD, CA-WD, NT+WD, and NT-WD. Plot **e** compares CA+Irrigation, CA-Irrigation, NT+irrigation, and NT-Irrigation. Plot **f** compares the productive performance of CA and NT in maize and winter maize systems.

**Figure 2:**
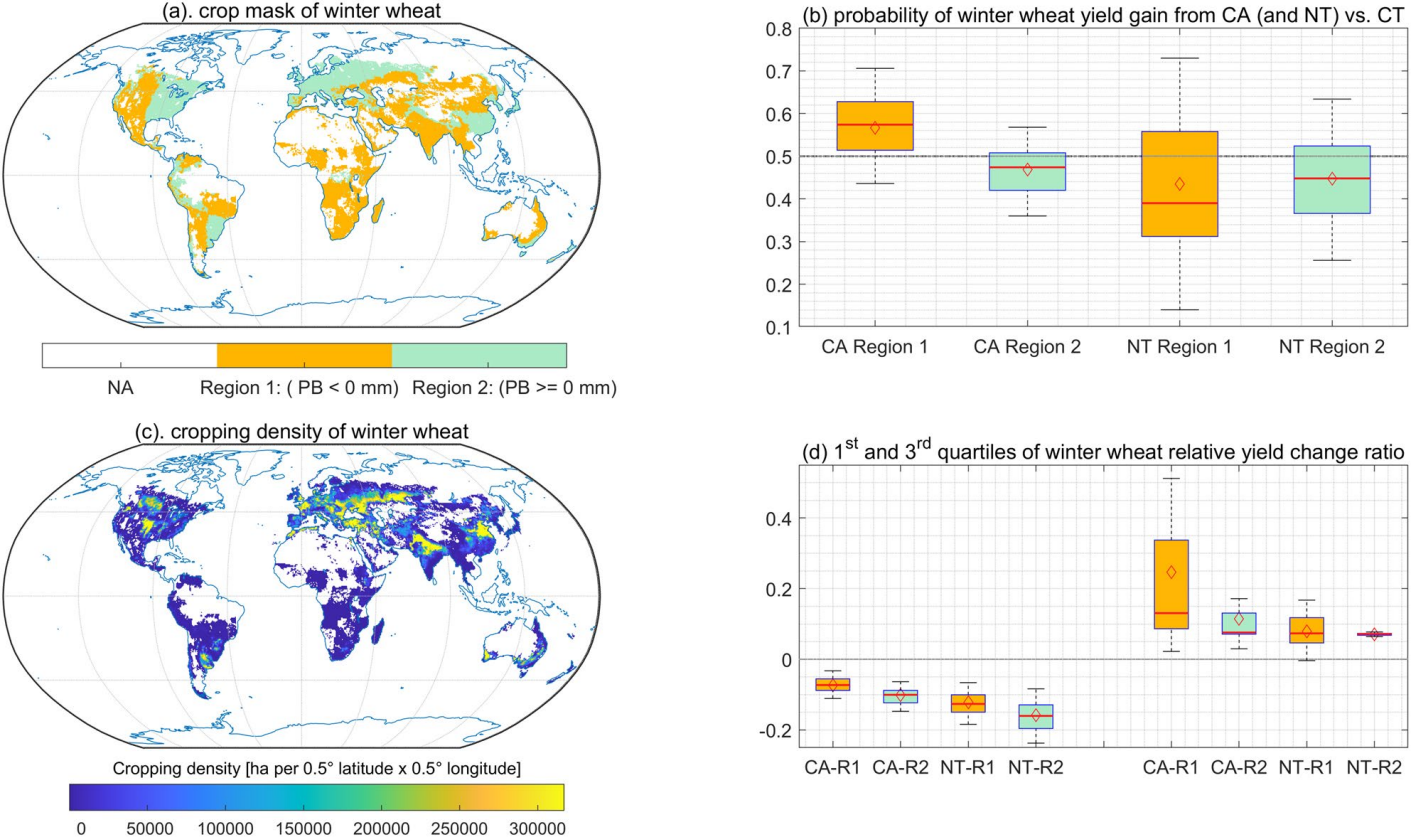
Productivity of CA and NT vs. CT (with fertilization and integrated weed and pest control) for winter wheat in a relatively dry (region #1) and a relatively wet (region #2) region. Plot **a** illustrates region #1 and region #2 on a global map with the two different colours showed in the sub-legend, the blank area indicates the non-cropping region of winter wheat or the regions without available climate data. PB represents precipitation balance over the crop growing season. Plot **b** shows the probability of winter wheat yield gain (CA and NT vs. CT). Plot **c** shows the global map of wheat cropping density^27^, the yellower shades indicate the higher density, and vice versa. The yellow colour in this map indicates the crop density is equal or higher than 20% of maximum density of crop in the cell of $0.5^\circ\, \mathrm{ latitude }\times 0.5^\circ \,\mathrm{ longitude}$ at the global scale. Plot **d** shows the 1st and 3rd quartiles of winter wheat relative yield change under CA and NT vs. CT in these two regions, and the x axis tick label in this plot: R1, R2 indicate region #1 or dry region; region #2 or wet region. The left part of plot **d** indicates the yield change ratios at the 1st quartile, while the right part represents the yield change ratios at 3rd quartile. The colours in plot **a**,**b**,**d** indicate the same regions (i.e., R1 and R2). In plot **b**,**d**, the mean value of relative yield change in its region is marked by the red diamond, while the median value is depicted by the red horizontal line.

The functional relationships among the probability of yield gain from CA (and NT) vs. CT, the climatic factors and the agricultural management practices were demonstrated through partial dependence plots^28,29^. The results showed that CA has better performance than NT due to the positive effects of soil cover and crop rotation on crop yield (Fig. 1a,b). Fertilizer application increased the productivity of CA and NT systems as well (Fig. 1c). The probability of yield gain from CA was slightly higher with weed and pest control under the dry conditions, while there was no significant effect of weed and pest control on NT systems (Fig. 1d). Irrigation improved the performance of NT systems, but decreased the competitiveness of CA vs. CT (Fig. 1e). Our results also showed that both CA and NT practices were likely to result in a better productivity in regions where water stress prevails compared to wetter conditions (Fig. 1). Here, we defined a relatively dry region (region #1) and a relatively wet region (region #2) based on the precipitation balance: region #1 indicates that the accumulated precipitation balance throughout the growing season is lower than 0 mm, while region #2 indicates a positive balance. Further analyses on the probability of yield increase and 1st and 3rd quartile of relative yield change for different crops in relatively dry (region #1) and wet (region #2) regions also showed that, in general, CA outperformed NT, and that CA and NT have better performance in regions that were relatively dry (Fig. 2d, Supplementary S8d, S11d, S14d, S16d, S19d, S22d, S25d). For winter wheat, the mean probability of yield gain with CA vs. CT is 56% and 47% in region #1 and region #2, respectively (Fig. 2b); The plausible range of yield change when shifting from CT to CA in region #1 is from − 0.11 to 0.51, and − 0.15 to 0.17 for region #2 (Fig. 2d).

**Figure 3:**
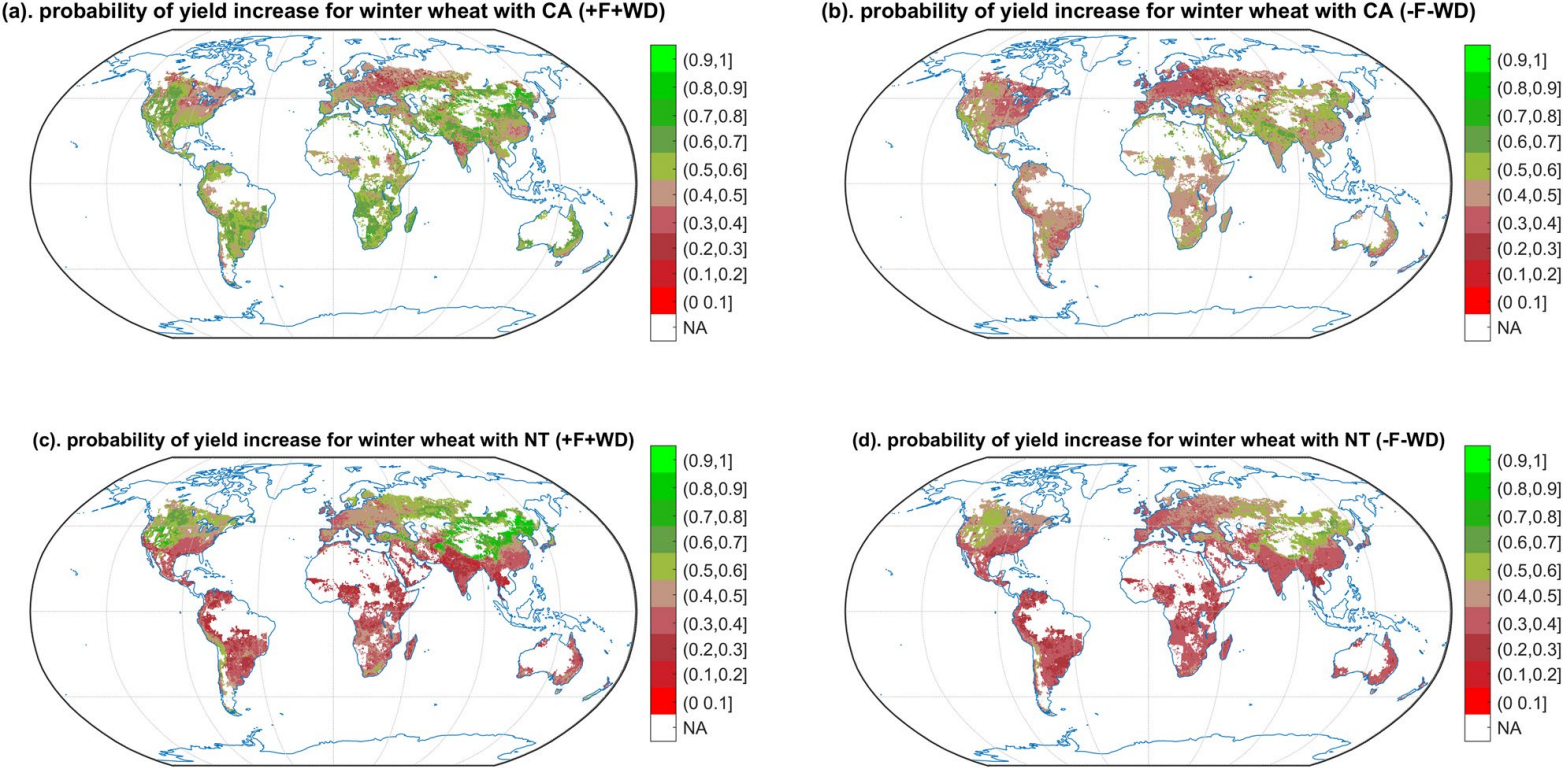
Probability of yield gain with CA and NT vs. CT winter wheat. Only the cropping regions are presented. The different colours indicate different probabilities of yield gain from CA and NT comparing to CT system. Greener shades indicate a higher probability of yield gain, while redder shades indicate a lower probability of yield gain. NA indicates the non-cropping region of winter wheat and the regions without available climate data. ± F indicates NT or CA and CT with/without fertilization. ± WD indicates NT or CA and CT with/without weed and pest control.

**Figure 4:**
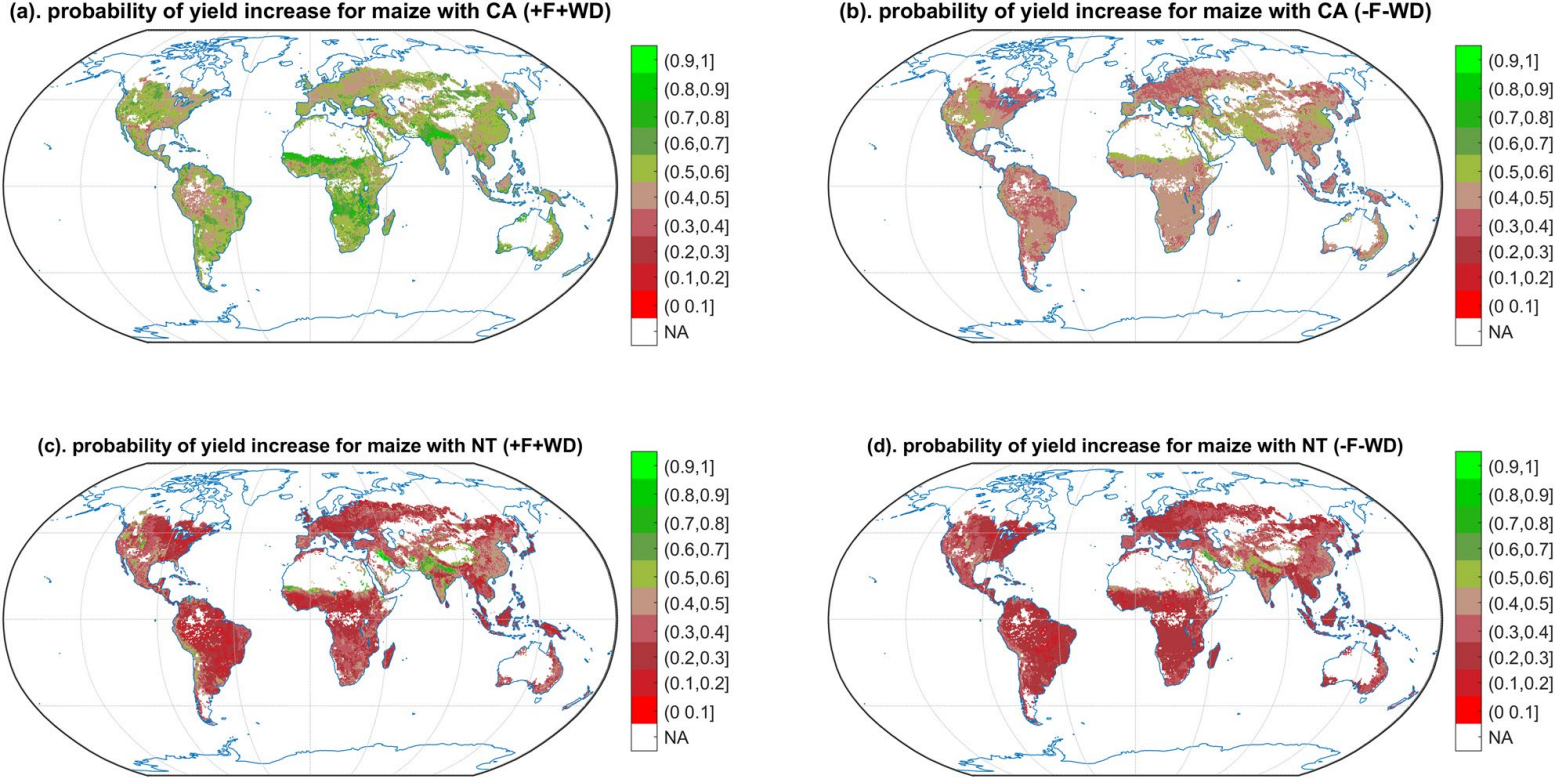
Probability of yield gain with CA and NT vs. CT maize. Only the cropping regions are presented. The different colours indicate different probabilities of yield gain from CA and NT comparing to CT system. Greener shades indicate a higher probability of yield gain, while redder shades indicate a lower probability of yield gain. NA indicates the non-cropping region of maize and the regions without available climate data. ± F indicates NT or CA and CT with/without fertilization. ± WD indicates NT or CA and CT with/without weed and pest control.

Probabilities of yield gain show important geographical variations. CA, in particular when associated with fertilization and weed and pest control, is likely to lead to a yield gain compared to CT in North-western America for spring barley (Supplementary S9a,b), maize (Fig. 4a,b), sorghum (Supplementary S20a,b), sunflower (Supplementary S26a,b) and winter wheat (Fig. 3a,b); In Pakistan and the west of India for spring barley (Supplementary S9a,b), cotton (Supplementary S12a,b), maize (Fig. 4a,b), sorghum (Supplementary S20a,b) and winter wheat (Fig. 3a,b); In north of China for cotton (Supplementary S12a,b), sorghum (Supplementary S20a,b), sunflower (Supplementary S26a,b), and winter wheat (Fig. 3a,b). On the other hand, CA, especially without fertilization and the control of weeds and pests, has lower probability of yield gain in tropical region for rice (Supplementary S17a,b), sorghum (Supplementary S20a,b), soybean (Supplementary S23a,b), sunflower (Supplementary S26a,b); In south of China for sorghum (Supplementary S20a,b), soybean (Supplementary S23a,b), winter wheat (Fig. 3a,b); In North-eastern America, and Europe for spring barley (Supplementary S9a,b), maize (Fig. 4a,b), soybean (Supplementary S23a,b), and winter wheat (Fig. 3a,b).

**Figure 5:**
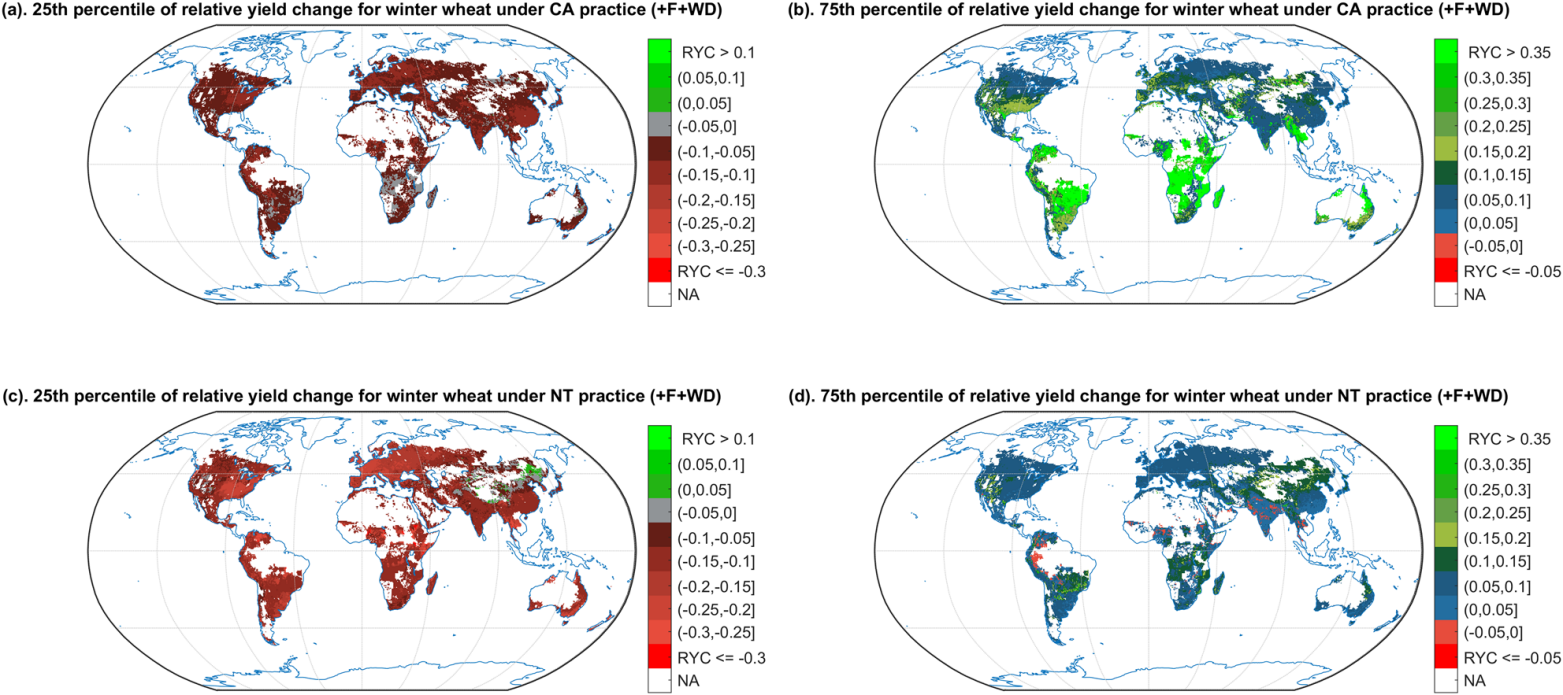
Relative yield change probability (1st and 3rd quartile estimate) of shifting CT to CA/NT for winter wheat, with fertilization and weed and pest control (+ F + WD). There is a 75% chance that the relative yield change will be higher than the ratio shown on the map in plot (**a**,**c**), and conversely a 25% chance that the relative change will be lower. There is a 75% chance that the relative yield change will be lower than the ratio shown on the map in plot (**b**,**d**), and conversely a 25% chance that the relative change will be higher. The colours indicate different levels of yield change ratio, and the red colour shades indicate negative yield changes or yield losses. NA indicate the non-cropping region of winter wheat and the regions without available climate data.

Here we show that, comparing with CT, CA with proper management has a 25% chance of producing large yield gains (more than 30%) in most of Africa for spring barley (Supplementary S10b), cotton (Supplementary S13b), maize (Supplementary S15b), sorghum (Supplementary S21b), soybean (Supplementary S24b), sunflower (Supplementary S27b), winter wheat (Fig. 5b); In some part of South America for spring barley (Supplementary S10b), cotton (Supplementary S13b), maize (Supplementary S15b), sorghum (Supplementary S21b), soybean (Supplementary S24b), sunflower (Supplementary S27b), winter wheat (Fig. 5b); In some part of South-eastern US for spring barley (Supplementary S10b), maize (Supplementary S15b), sorghum (Supplementary S21b), soybean (Supplementary S24b), sunflower (Supplementary S27b). Conversely, NT has a 75% chance of increasing yield by more than 5% for winter wheat in the northeast of China (Fig. 5c).

However, even with proper agricultural management practices, there is a 75% chance that NT will lead to a yield decrease in some part of the tropical regions for cotton (Supplementary S13d), maize (Supplementary S15d), rice (Supplementary S18d), sorghum (Supplementary S21d), soybean (Supplementary S24d), and sunflower (Supplementary S27d); In some part of China for rice (Supplementary S18d), sorghum (Supplementary S21d), soybean (Supplementary S24d), and sunflower (Supplementary S27d); In eastern EU for maize (Supplementary S15d), sorghum (Supplementary S21d), and soybean (Supplementary S24d); And in North-eastern America for maize (Supplementary S15d).

## Discussion

As the probabilities and plausible ranges of yield gain and loss with CA and NT systems have not been mapped in previous meta-analyses^1,9,11^, our results bring meaningful and novel information to policymakers and agricultural extension services. In this study, we were able to identify the regions that have higher or lower probability of yield gain from shifting CT to CA and NT for eight major field crops. The magnitude of these gains was assessed, as well as the potential yield losses.

Although based on an expanded dataset, our study has several limitations. Most of the data collected pertains to humid climates rather than arid regions. Crop irrigation was considered only as a categorical variable here due to a lack of global data on this practice, but still proved meaningful in terms of yield impacts. Finally, to deal with missing climate and soil data in the selected papers, we used climate and soil data from external databases on a systematic basis. Consequently, crop growing season, precipitation, potential evapotranspiration, minimum temperature, average temperature, maximum temperature throughout the growing season, and soil texture may not always match local records. However, the use of external databases allowed us to analyse the effects of the inter-annual and intra-annual climate variabilities on CA and NT productivity.

Our study revealed large differences in the likelihood of yield gains associated with the adoption of CA (or NT) across crops, crop management practices, geographical regions, and climatic conditions. Based on our results, NT appeared more likely to increase yields in dry conditions compared to wet conditions, especially when it combined with soil cover. The potential benefits of such practice are well known.

The layer of crop residues retained on the soil surface in no-till systems reduces soil evaporation and water runoff^30,31^, fosters the build-up of organic matter in soils^32^, preserves soil water resources for crops^33^, increases soil water retention capacity and mitigates drought effects^34–36^. These factors all contribute to increase the probability of yield gain. Conversely, in humid regions, the comparative advantages of CA or NT with soil cover were no longer evident and can even be detrimental in the case of soils prone to waterlogging^1^. In some other conditions, such as winter crop in cold region, we did notice that not covering the soil increased the chance of yield gain compared to continuous soil cover. We showed that winter wheat in northeast of China, NT has better performance than CA, this might be because the soil cover reduced the mean soil temperature^37^, which delayed the crop establishment and growth^4,38,39^. However, it is also reported that residue cover could decrease the rate of soil temperature change^37^, increase the minimum soil temperature in extreme cold conditions^40–42^, and provide a buffer layer that can increase the crop resistance to the increasing climate variability and the occurrence of extreme events^4^. Therefore, soil cover reduces the risk of crop failure and increased yield stability. The fact that soil cover management had the largest positive impact on the productivity of NT, compared to other management practices including crop rotation and the control of weeds and pests, was also reported in previous studies^1,43^. Despite the recognized positive effects of residue cover and crop rotation, these two practices were not always implemented with NT systems^44^.

Our results also showed that, with integrated weed and pest management, CA systems tend to perform slightly better than without (Fig. 1d), which might indirectly suggest that the crop residue cover used in CA may increase the weed or pest pressure in dry conditions^45,46^. While in humid condition, CA might have a slightly higher probability of yield gain in the absence of weed control. This may be due to a greater competition for water resources between crops and weeds when weeds were not controlled^47,48^, leading to dryer conditions and to an increased probability of yield gain with CA. Our results showed again that CA and NT had a higher probability of yield gain under fertilized conditions^1^.

Overall, we showed that CA has a better productivity than NT, especially when combined with the proper agronomical practices of fertilization and integrated weed and pest management. Therefore, we recommend that NT systems should be implemented with soil cover, crop rotation (thus following the definition of Conservation Agriculture by the FAO), crop fertilization, integrated weed and pest management, and all the other good agronomic practices like good seed, water management. Although CA may not always outperform CT concerning on crop yield, CA can provide a range of ecosystem services far beyond biomass production, those ecosystem services included improve the soil health, reduce the soil erosion risk, etc.^49^. The present traditional tillage systems are resulting in serious land degradation, which will increase the risk of food insecurity in the future, and increase emissions and reduce carbon sinks^50^. Therefore, CA is a promising practice that can be promoted to sustain long-term food production.

## Supplementary Information

Supplementary Information.
