# Supplementary material for: High probability of yield gain through conservation agriculture in dry regions for major staple crops
Source: Sci Rep. 2021 Feb 8;11:3344. doi: 10.1038/s41598-021-82375-1 (PMC7870656; doi:10.1038/s41598-021-82375-1)
Supplement: Supplementary file 1 — Supplementary Information. [file 41598_2021_82375_MOESM1_ESM.pdf]

High probability of yield gain through conservation agriculture in dry regions for major staple crops

Authors

Yang Su[a], Benoit Gabrielle[a], Damien Beillouin[b,d,e], David Makowski[b,c]

[a] UMR ECOSYS, INRAE AgroParisTech, Université Paris-Saclay, 78850 Thiverval-Grignon, France

[b] UMR Agronomie, INRAE AgroParisTech, Université Paris-Saclay, 78850 Thiverval-Grignon, France

[c] Applied mathematics and computer science (MIA 518), INRAE AgroParisTech, Université Paris-Saclay, 75005 Paris, France.

[d] CIRAD, UPR HortSys, F-34398 Montpellier, France

[e] HortSys, Univ Montpellier, CIRAD, Montpellier, France

Corresponding Author

Yang SU yang.su@inrae.fr +33 1 30 81 55 51 INRAE AgroParisTech

S1 Flow chart of literature search and data collection

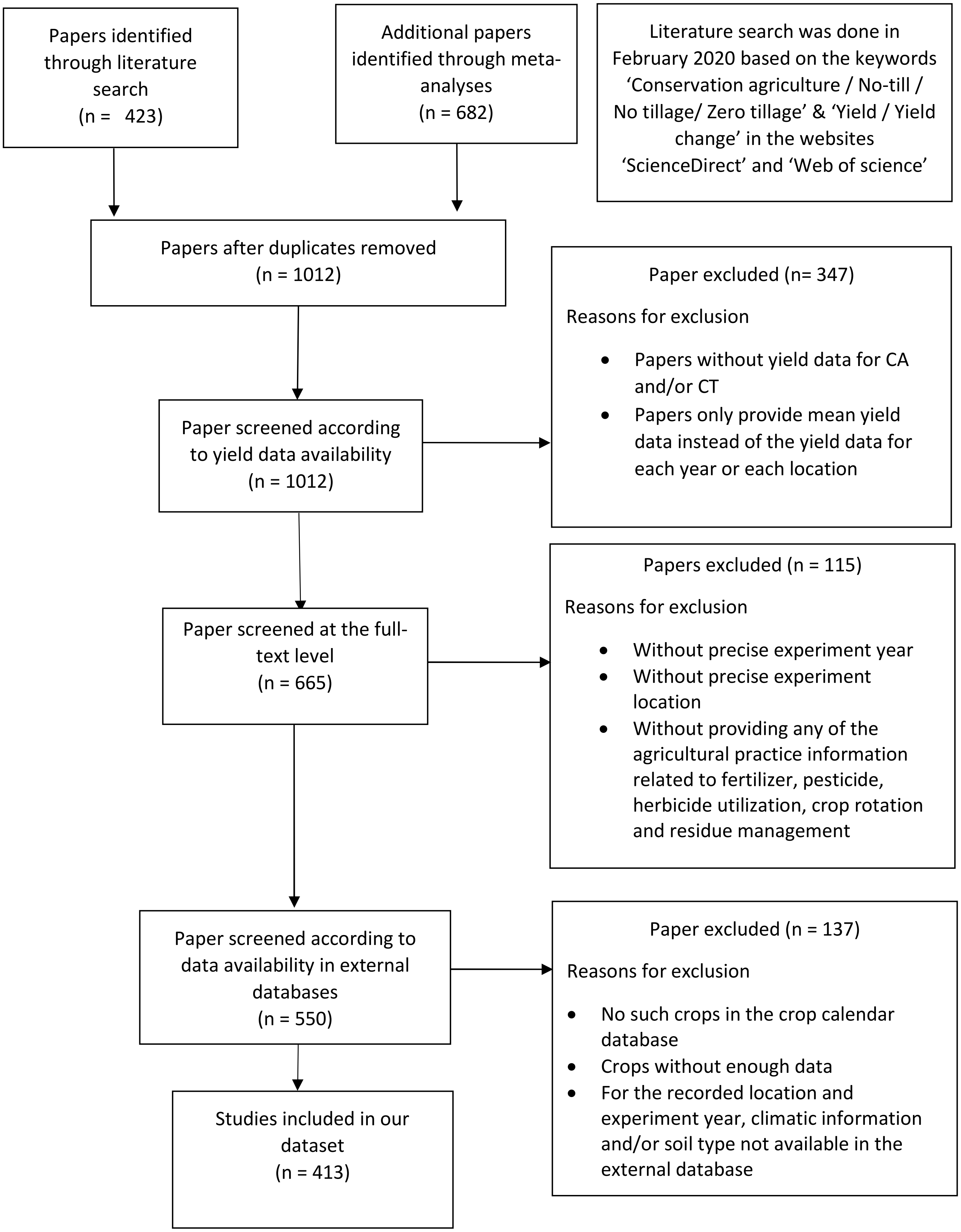

S2 Locations of experiments included in the dataset

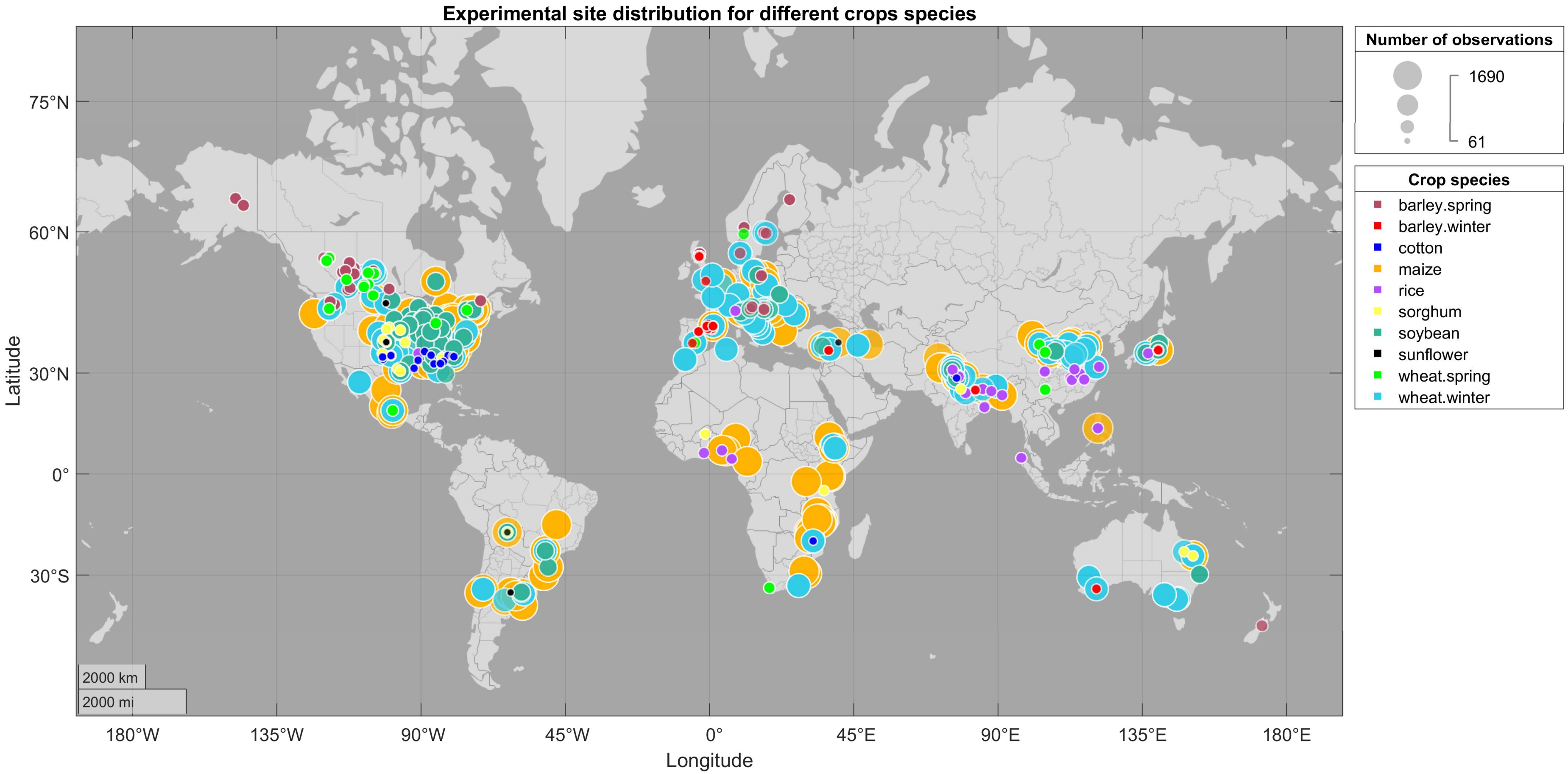

S3 Ability of the random forest model to discriminate between yield ratio (CA and CA like systems vs. CT) higher and lower than one. ROC curves were computed by leave one out cross validation (LOOCV).

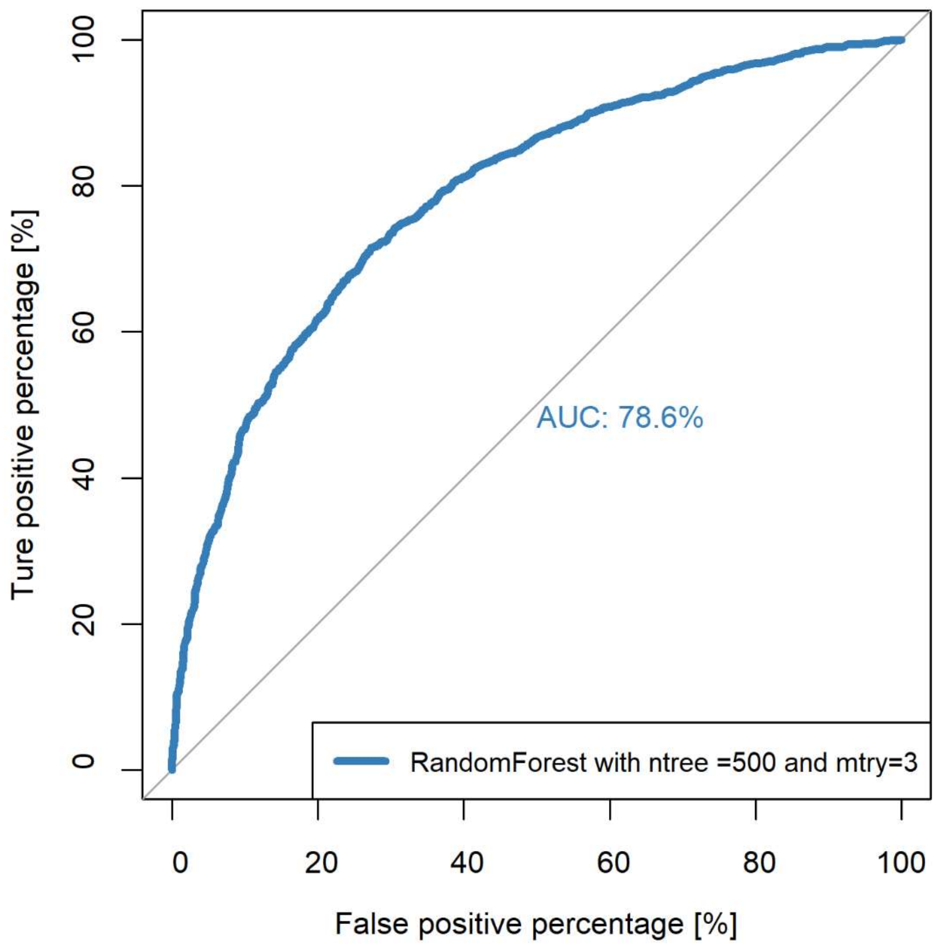

Cross-validation:

One yield ratio (and its associated set of inputs) was removed from the dataset at each iteration of the LOOCV, and the rest of the dataset was used to train the random forest model. The trained model was then used to compute the probability of yield gain from the input data of the removed yield ratio. This procedure was repeated for all yield ratios. The R package "randomForest" was used. "randomForest" is a standard package for implementing ensemble of regression trees and is very robust [1]. The probabilities of yield gain computed by the package were used to compute the Area under the ROC curve (AUC) with the R package "pROC". AUC is equal to the probability that the classifier (here the output of the random forest algorithm) will rank a random pair of positive (ratio>1) and negative (ratio<=1) cases correctly [2], so a higher AUC means a better model classification performance. Here we see that AUC of "randomForest" is 78.6%, which indicates that the probability that random forest model can classify a pair of observed "yield gain" and "yield loss" correctly is 78.6%.

S4 Ability of quantile regression forests model to predict the level of yield change from shifting CT to CA or CA like systems. Result based on leave one out cross validation (LOOCV) and the coverage probability

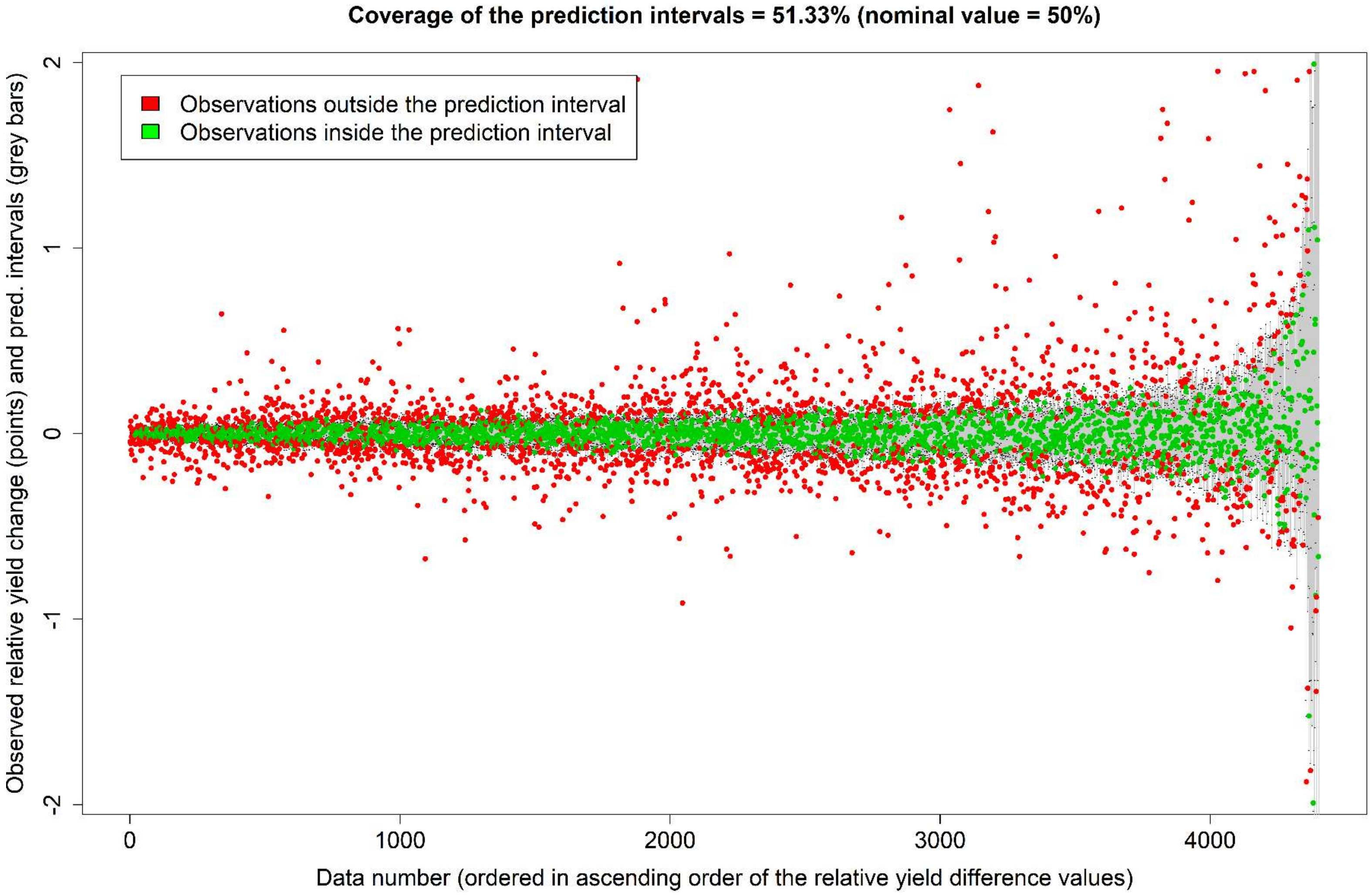

Cross-validation:

The performance of the quantile regression forest (based on “ranger” package in R) was evaluated by computing the proportion of data within the prediction intervals estimated by the model. When this proportion is close to the expected coverage probability, the intervals calculated by the quantile regression forest can be considered accurate. This approach was implemented here by LOOCV. One relative yield change data (and its associated set of inputs) was thus removed from the dataset at each iteration of the LOOCV, and the rest of the dataset was used to train quantile regression forest model. The trained model was then used to compute the 25th and 75th percentiles of the relative yield change from the input values of the removed data. This procedure was repeated for all relative yield change data, and the proportion of data within the prediction intervals was computed and compared to its expected value, i.e. 50%. In S4, the vertical grey bars with two black dots correspond to the intervals defined by the 25th and 75th percentiles and the points correspond to observed relative yield changes. Data within the intervals are in green and data outside are in red.

S5 Model setting for global projection of probability of yield grain and of the 1st and 3rd quartiles of relative yield change.

Model input	Setting (for each grid cell and each crop)	Source
Precipitation	Mean precipitation in the growing season of 1981-2010	National Oceanic and Atmospheric Administration (NOAA)[3]
Evapotranspiration	Mean potential evapotranspiration in the growing season of 1981-2010	Data from GLEAM (Global Land Evaporation Amsterdam Model) [4,5]
Average temperature	Mean average temperature in the growing season of 1981-2010	National Oceanic and Atmospheric Administration (NOAA)[3]
Maximum temperature	Mean maximum temperature in the growing season of 1981-2010	National Oceanic and Atmospheric Administration (NOAA)[3,6]
Minimum temperature	Mean minimum temperature in the growing season of 1981-2010	National Oceanic and Atmospheric Administration (NOAA)[6]
Soil texture	HWSD data	The University of Tokyo[7]
Crop growing season	Month of planting to harvesting in the crop calendar dataset for each crop	University of Wisconsin-Madison[8,9]
Crop mask	MIRCA2000 data	Goethe University[10]
Irrigation mask	MIRCA2000 data	Goethe University[10]
Crop type	Spring barley, cotton, maize, rice, sorghum, soybean, sunflower, winter wheat	
Fertilizer utilization in NT/CA	Yes (+F) or No (-F)	
Fertilizer utilization in CT	Yes (+F) or No (-F)	
Control of weed and pest in NT/CA	Yes (+WD) or No (-WD)	
Control of weed and pest in CT	Yes (+WD) or No (-WD)	
Crop rotation in NT/CA	With crop rotation for CA, without crop rotation for NT by default. In Figure 1b, NT (+/- R) indicated NT with/without crop rotation.	
Crop rotation in CT	Without crop rotation	
Soil cover in NT/CA	With soil cover for CA, without soil cover for NT by default. In Figure 1a, NT (+/- SC) indicated NT with/without soil cover.	
Soil cover in CT	Without soil cover	
Crop irrigation in NT/CA	Based on crop irrigation mask by default. In figure 1e, NT/CA/CT (+/- Irrigation) indicated NT/CA/CT with/without irrigation.	Goethe University[10]
Crop irrigation in CT	Based on crop irrigation mask by default. In figure 1e, NT/CA/CT (+/- Irrigation)	Goethe University[10]

	indicated NT/CA/CT with/without irrigation	

S6 Flow chart showing the algorithm of making one-dimensional partial dependence plot

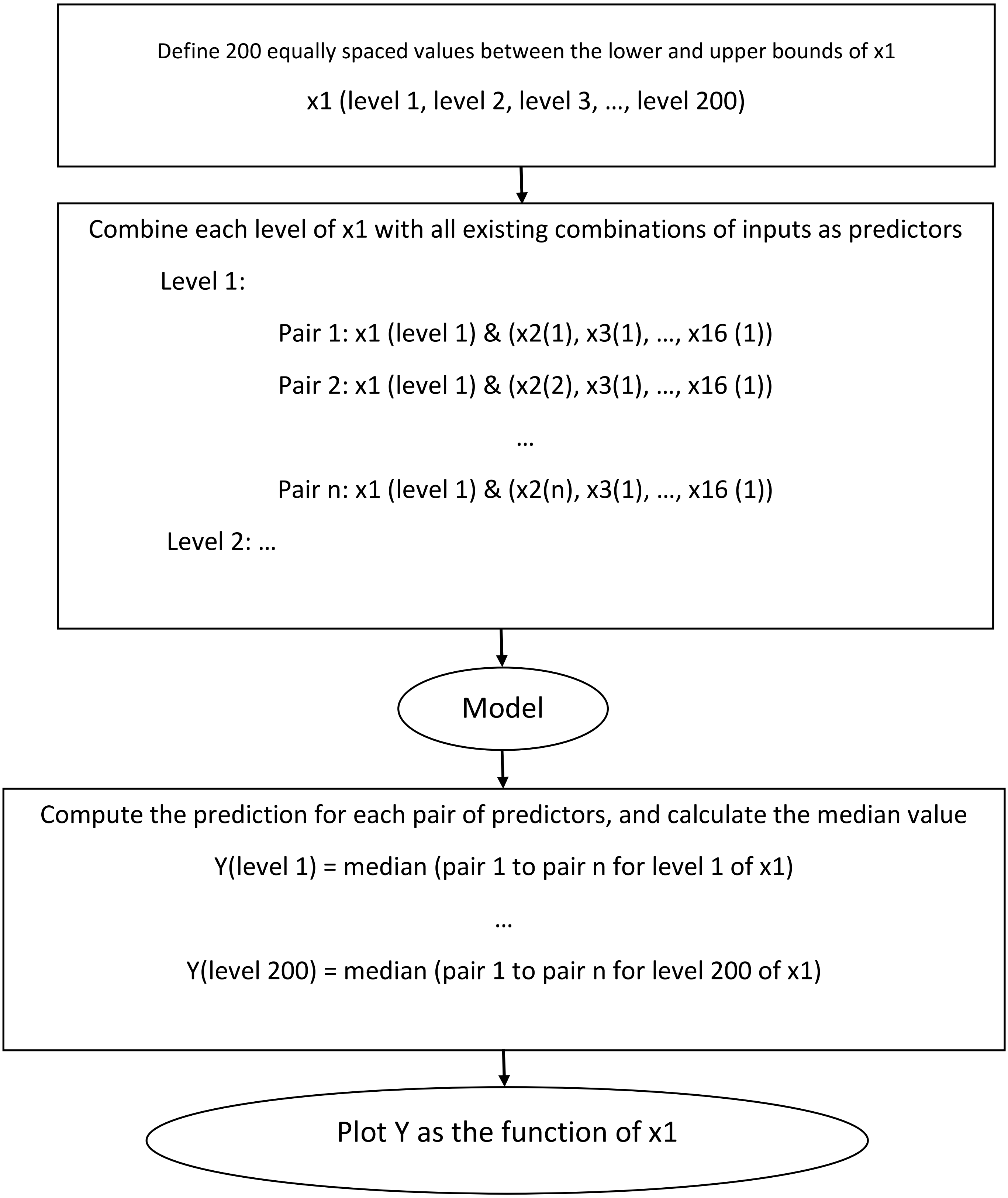

Here, x1 represented precipitation balance (PB). The ranges of PB values were divided into 200 values and the probability of yield gain resulting from CA or NT vs. CT was calculated using the random forest model for all combinations of the other input variables included in the dataset. The resulting probabilities were then averaged for each level of PB, and the medians were plotted in a one-dimensional plot. The default setting of different crop systems is, NT: without crop rotation and without soil cover; CA: with crop rotation and with soil cover; CT: without crop rotation and without soil cover. And in Figure 1a NT: without crop rotation but with soil cover; In Figure 1b NT: with crop rotation but without soil cover. As for other crop managements such as fertilization, what is compared in this paper is CA with fertilization or NT with fertilization vs. CT with fertilization.

S7 Relative importance ranking of the model inputs. The importance was defined by the mean decrease of Gini impurity in random forest model or quantile regression forest model

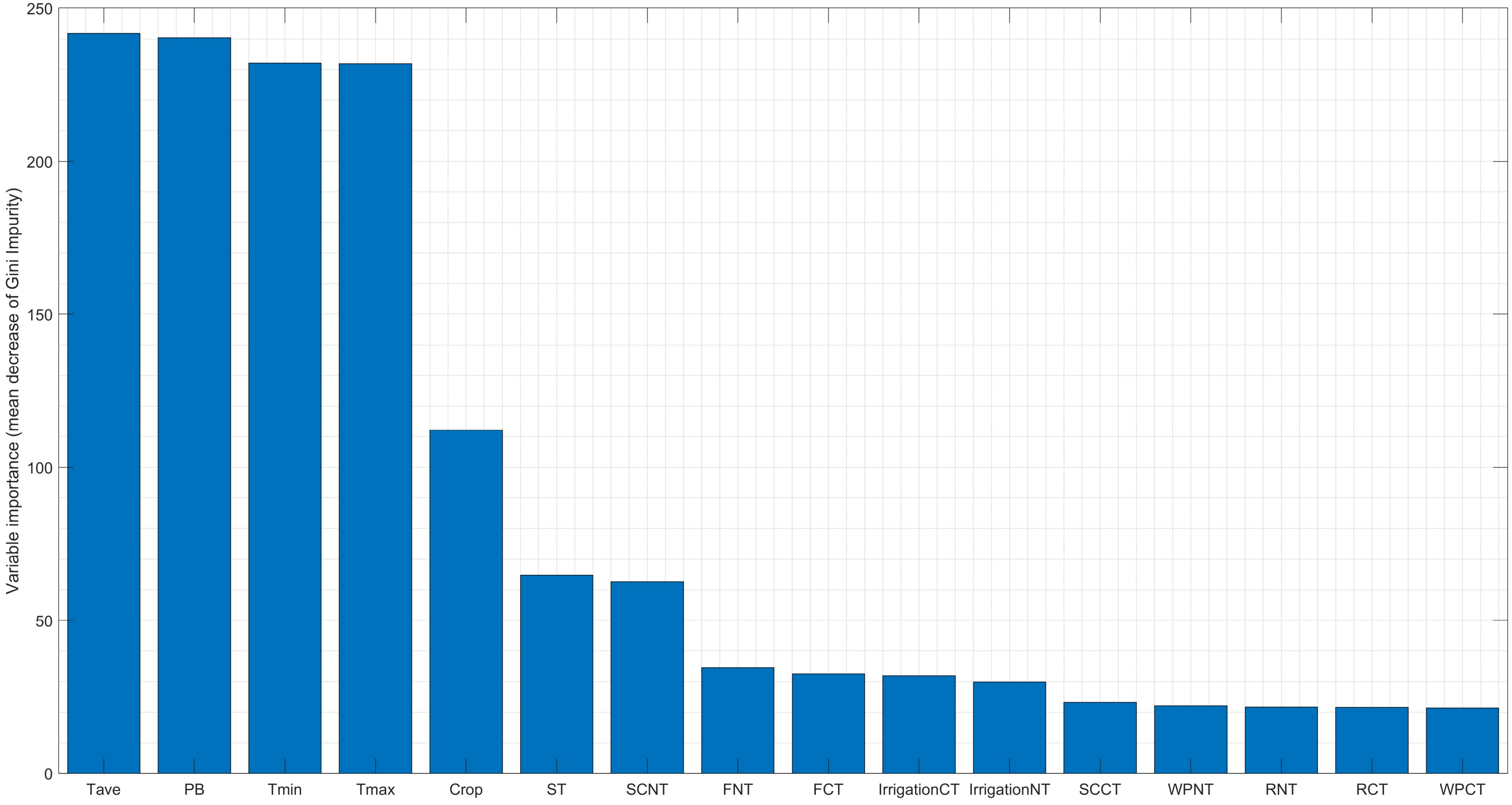

S8 Productive performance of CA and NT vs. CT for spring barley in relatively dry and relatively wet regions

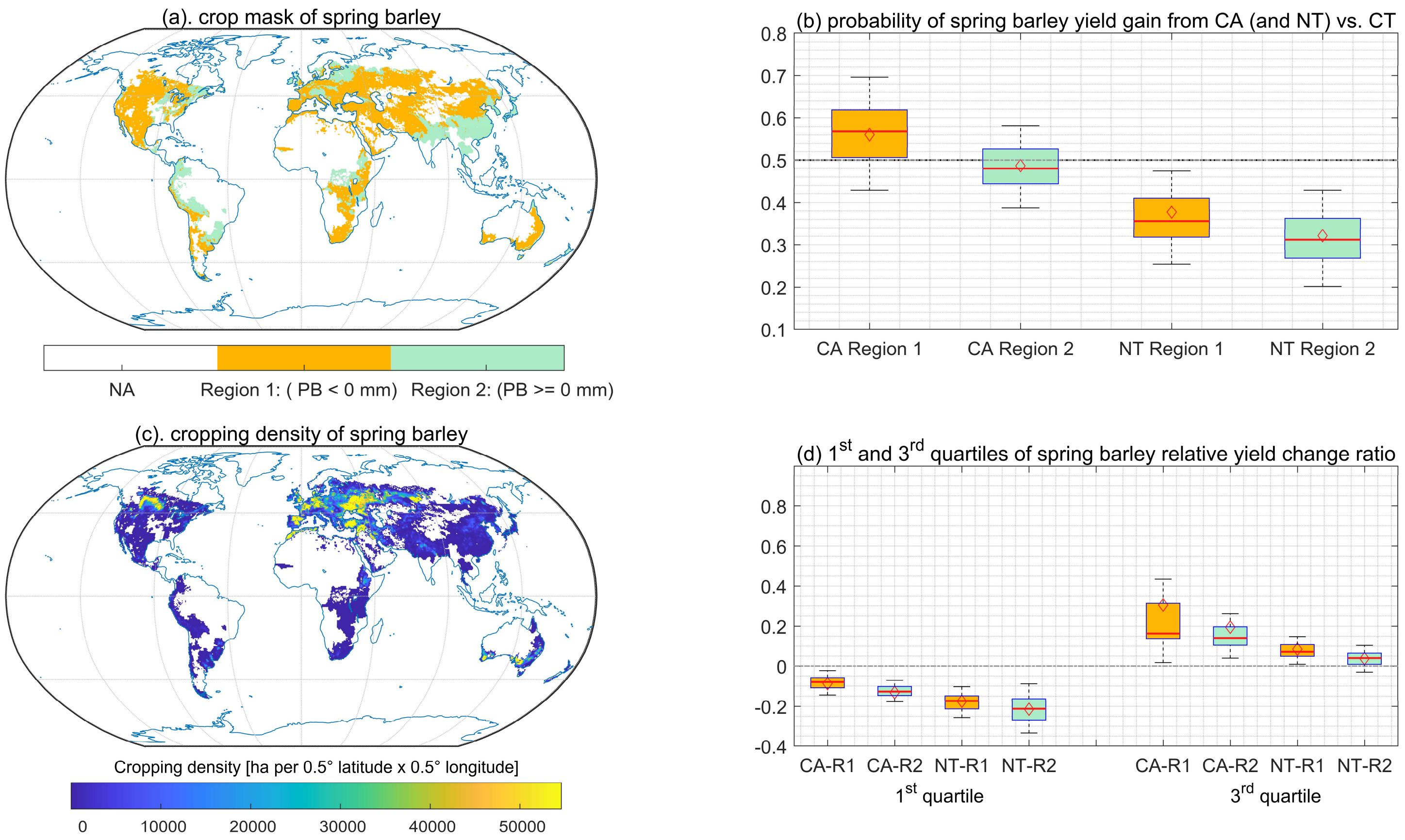

Productivity of Conservation Agriculture for spring barley in a relatively dry (region #1) and a relatively wet (region #2) region. (a) Illustration of the two regions on a global map with the two different colours showed in the sub-legend of plot a, while the blank area indicated the non-cropping region of spring barley. (b) The probability of spring barley yield gain (CA and NT vs. CT). (c) Barley cropping density on global map, the yellow shades indicated the higher density, and vice versa. The yellow colour in this map indicated the crop density is equal or higher than 20% of maximum density in the cell of 0.5° latitude ×0.5° longitude at the global scale. (d) The 1st and 3rd quartiles of spring barley relative yield change under CA and NT vs. CT practice in the two regions, and the x axis tick label in plot d: R1, R2 indicated the two different regions, and the left part of plot d indicated the yield change ratios at the 1st quartile, while the right part were the yield change ratios at 3rd quartile. The colours in plot a, b and d indicated the same regions. In plot b and d, the mean value of relative yield change in its region is marked by the red diamond, while the median value is depicted by the red horizontal line.

S9 The probability of yield gain of spring barley with CA and NT vs. CT practice

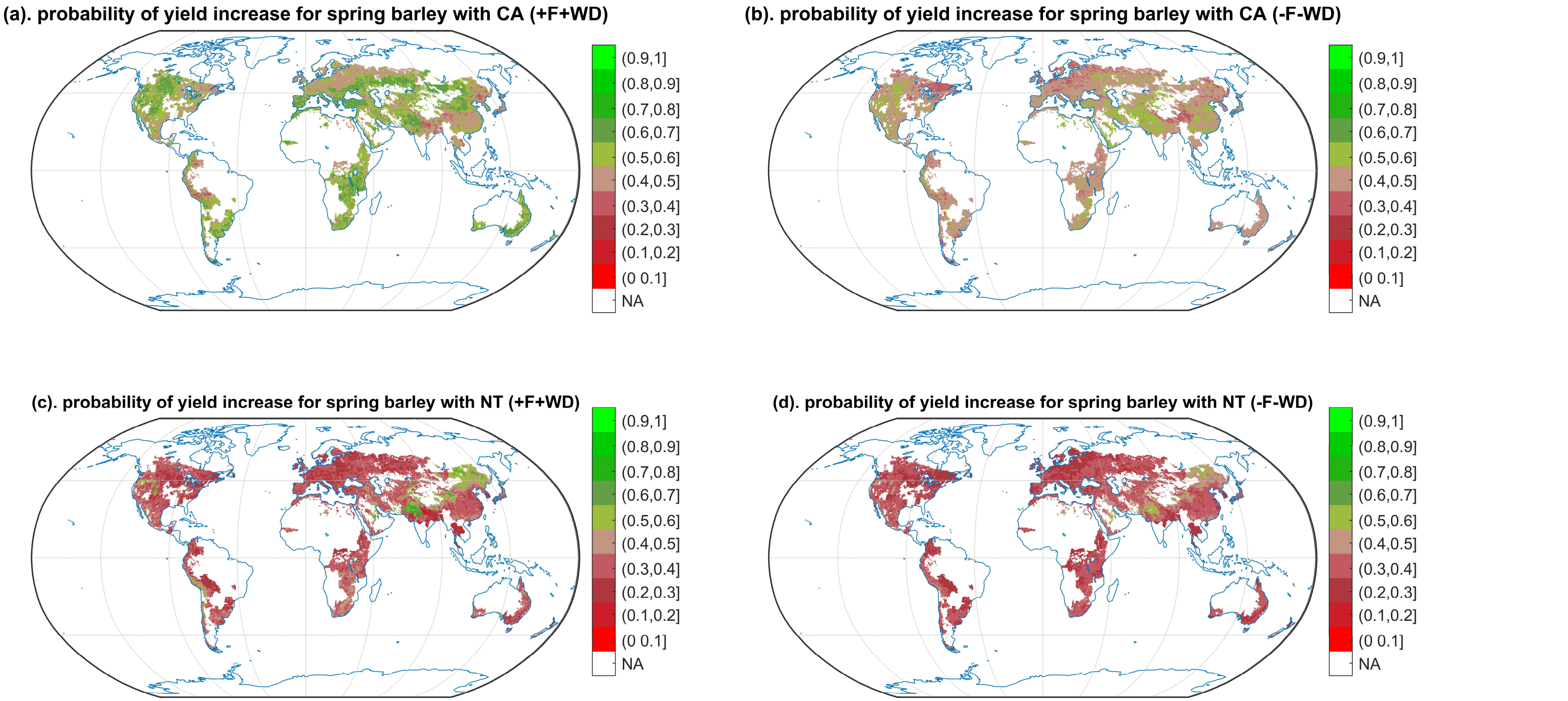

Probability of yield gain with CA and NT vs. CT spring barley. Only the cropping regions were presented. The different colours indicated different probabilities of yield gain from CA and NT comparing to CT system. The greener colour indicated a higher probability of yield gain. +/- F indicated NT or CA and CT with/without field fertilization. +/- WD indicated NT or CA and CT with/without weed and pest control.

S10 The relative yield change of spring barley with CA and NT vs. CT practice

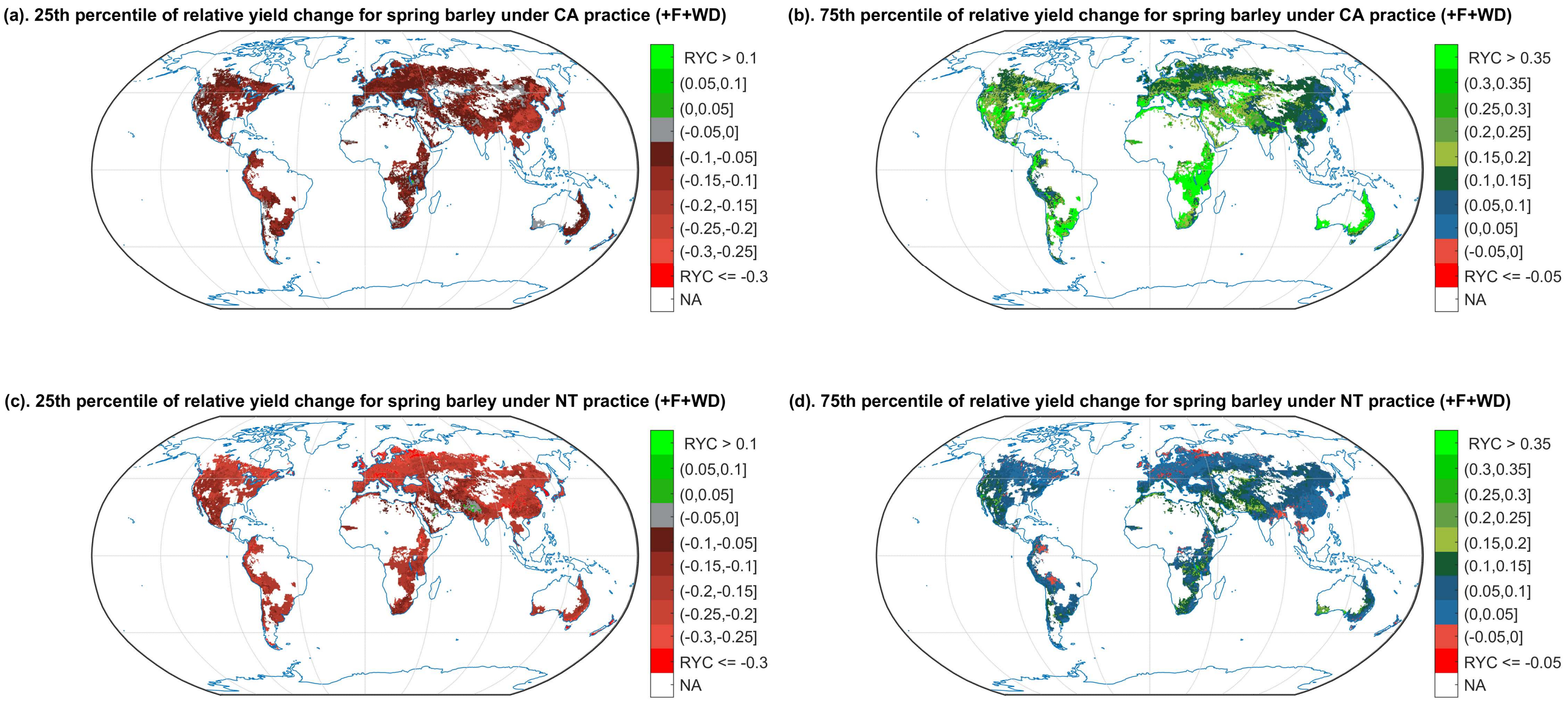

Relative yield change probability (1st and 3rd quartile estimate) of shifting CT to CA/NT for spring barley, with field fertilization and weed and pest control (+F+WD). There was a 75% chance that the relative yield change will be higher than the ratio shown on the map in plot a and c, and conversely a 25% chance that the relative change will be lower. There was a 75% chance that the relative yield change will be lower than the ratio shown on the map in plot b and d, and conversely a 25% chance that the relative change will be higher. The colours indicated different levels of yield change ratio, and the reddish colours indicated the negative yield change or yield loss.

S11 Productive performance of CA and NT vs. CT for cotton in relatively dry and relatively wet regions

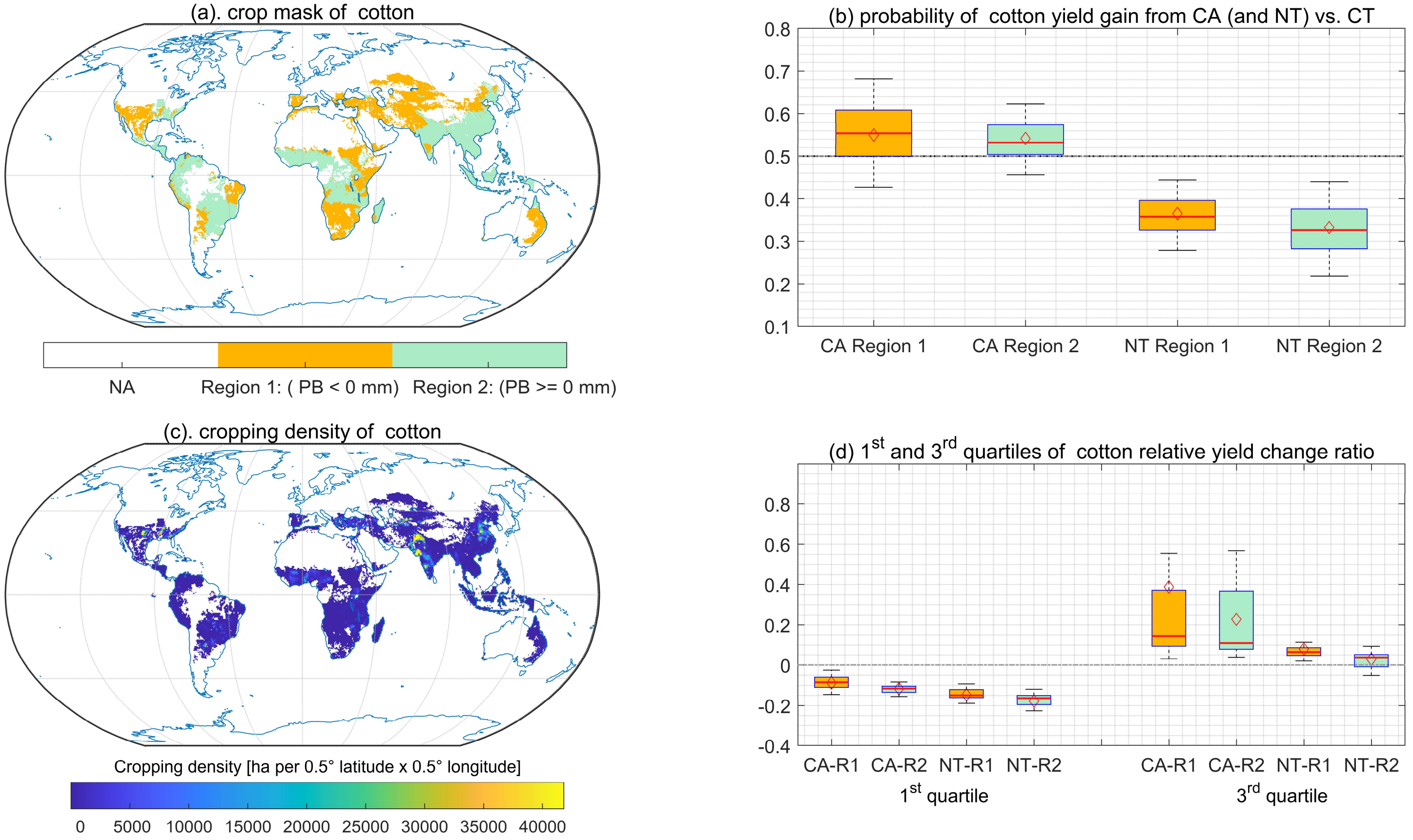

Productivity of Conservation Agriculture for cotton in a relatively dry (region #1) and a relatively wet (region #2) region. (a) Illustration of the two regions on a global map with the two different colours showed in the sub-legend of plot a, while the blank area indicated the non-cropping region of cotton. (b) The probability of cotton yield gain (CA and NT vs. CT). (c) Cotton cropping density on global map, the yellow shades indicated the higher density, and vice versa. The yellow colour in this map indicated the crop density is equal or higher than 20% of maximum density in the cell of 0.5° latitude ×0.5° longitude at the global scale. (d) The 1st and 3rd quartiles of cotton relative yield change under CA and NT vs. CT practice in the two regions, and the x axis tick label in plot d: R1, R2 indicated the two different regions, and the left part of plot d indicated the yield change ratios at the 1st quartile, while the right part were the yield change ratios at 3rd quartile. The colours in plot a, b and d indicated the same regions. In plot b and d, the mean value of relative yield change in its region is marked by the red diamond, while the median value is depicted by the red horizontal line.

S12 The probability of yield gain of cotton with CA and NT vs. CT practice

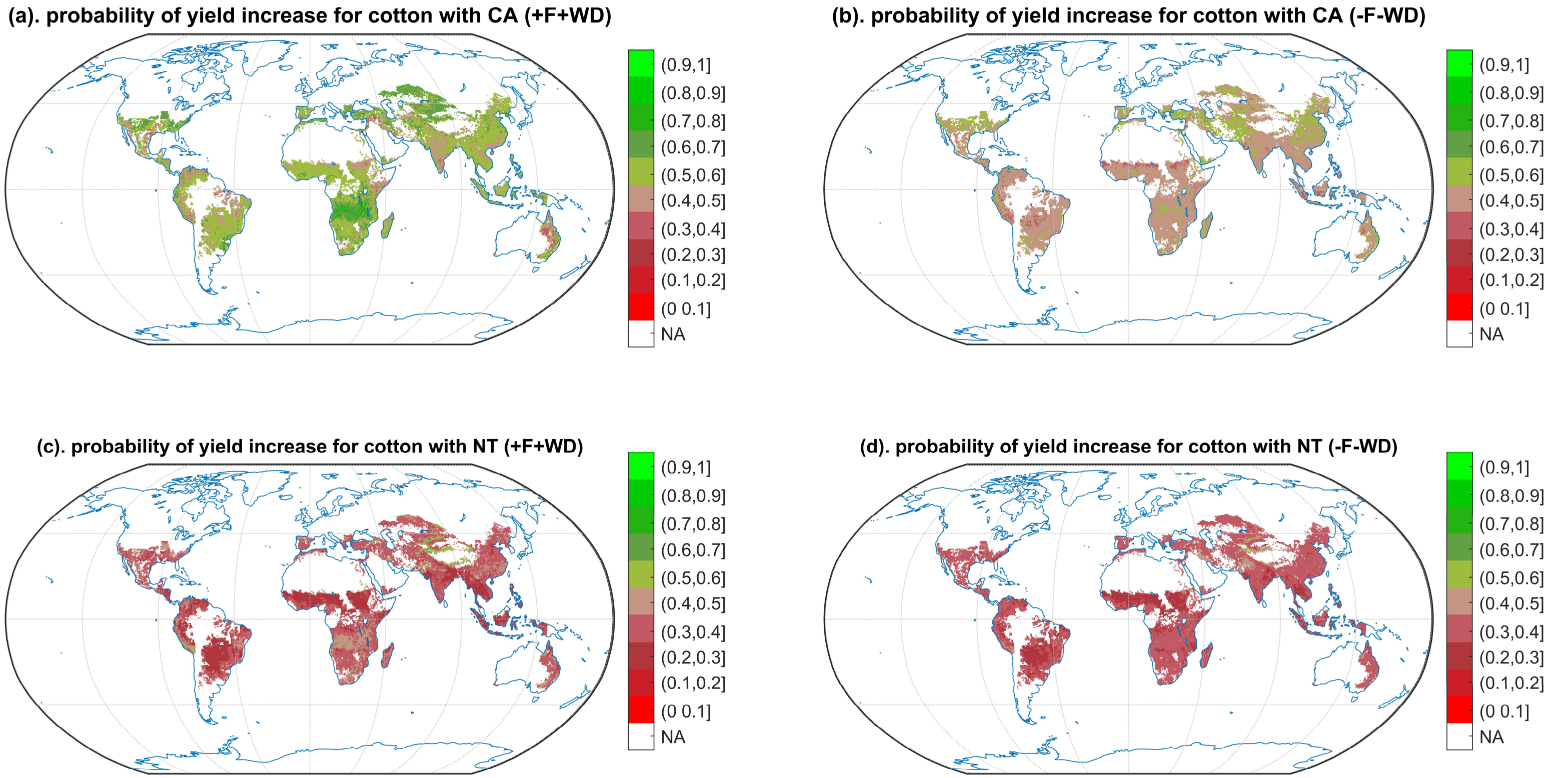

Probability of yield gain with CA and NT vs. CT cotton. Only the cropping regions were presented. The different colours indicated different probabilities of yield gain from CA and NT comparing to CT system. The greener colour indicated a higher probability of yield gain. +/- F indicated NT or CA and CT with/without field fertilization. +/- WD indicated NT or CA and CT with/without weed and pest control.

S13 The relative yield change of cotton with CA and NT vs. CT practice

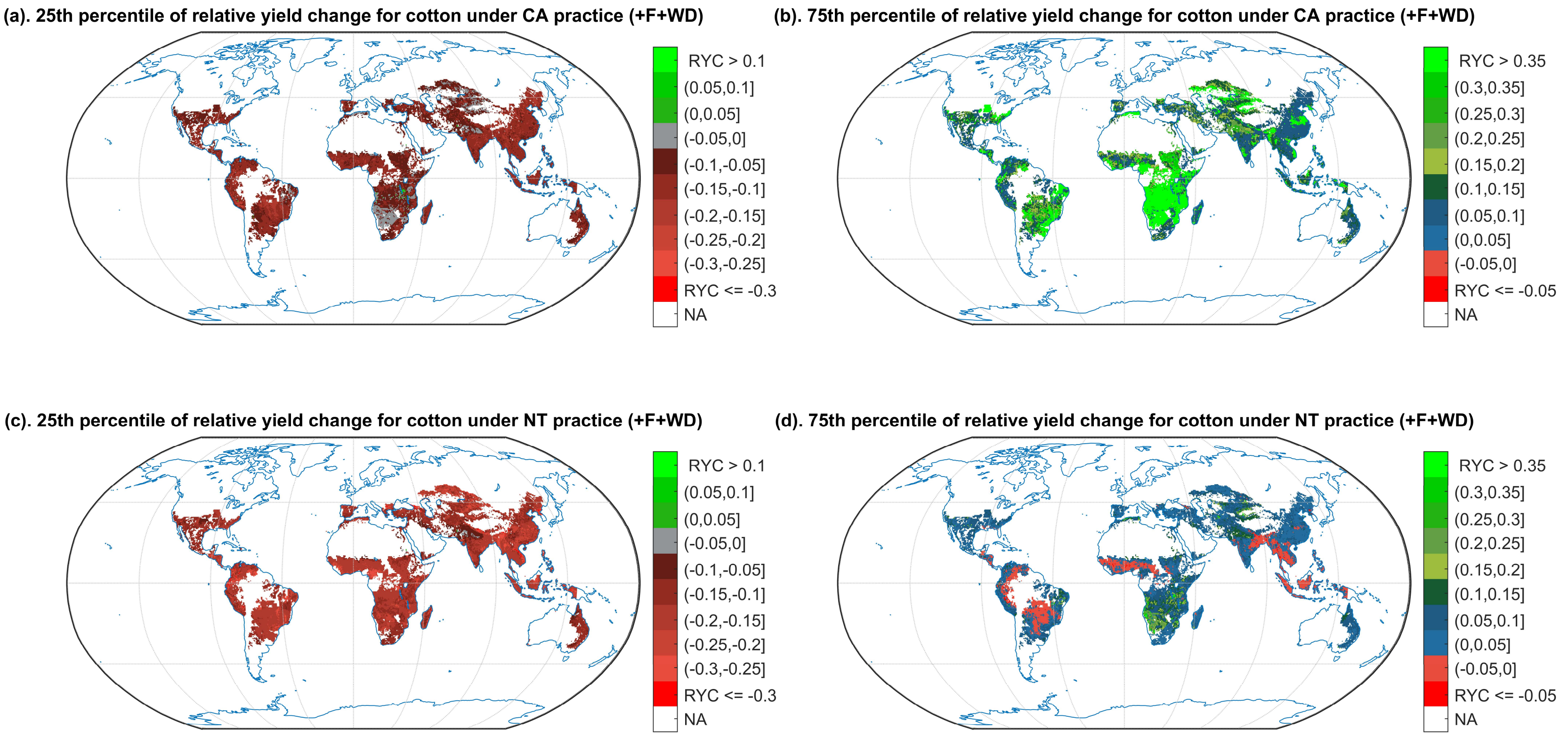

Relative yield change probability (1st and 3rd quartile estimate) of shifting CT to CA/NT for cotton, with field fertilization and weed and pest control (+F+WD). There was a 75% chance that the relative yield change will be higher than the ratio shown on the map in plot a and c, and conversely a 25% chance that the relative change will be lower. There was a 75% chance that the relative yield change will be lower than the ratio shown on the map in plot b and d, and conversely a 25% chance that the relative change will be higher. The colours indicated different levels of yield change ratio, and the reddish colours indicated the negative yield change or yield loss.

S14 Productive performance of CA and NT vs. CT for maize in relatively dry and relatively wet regions

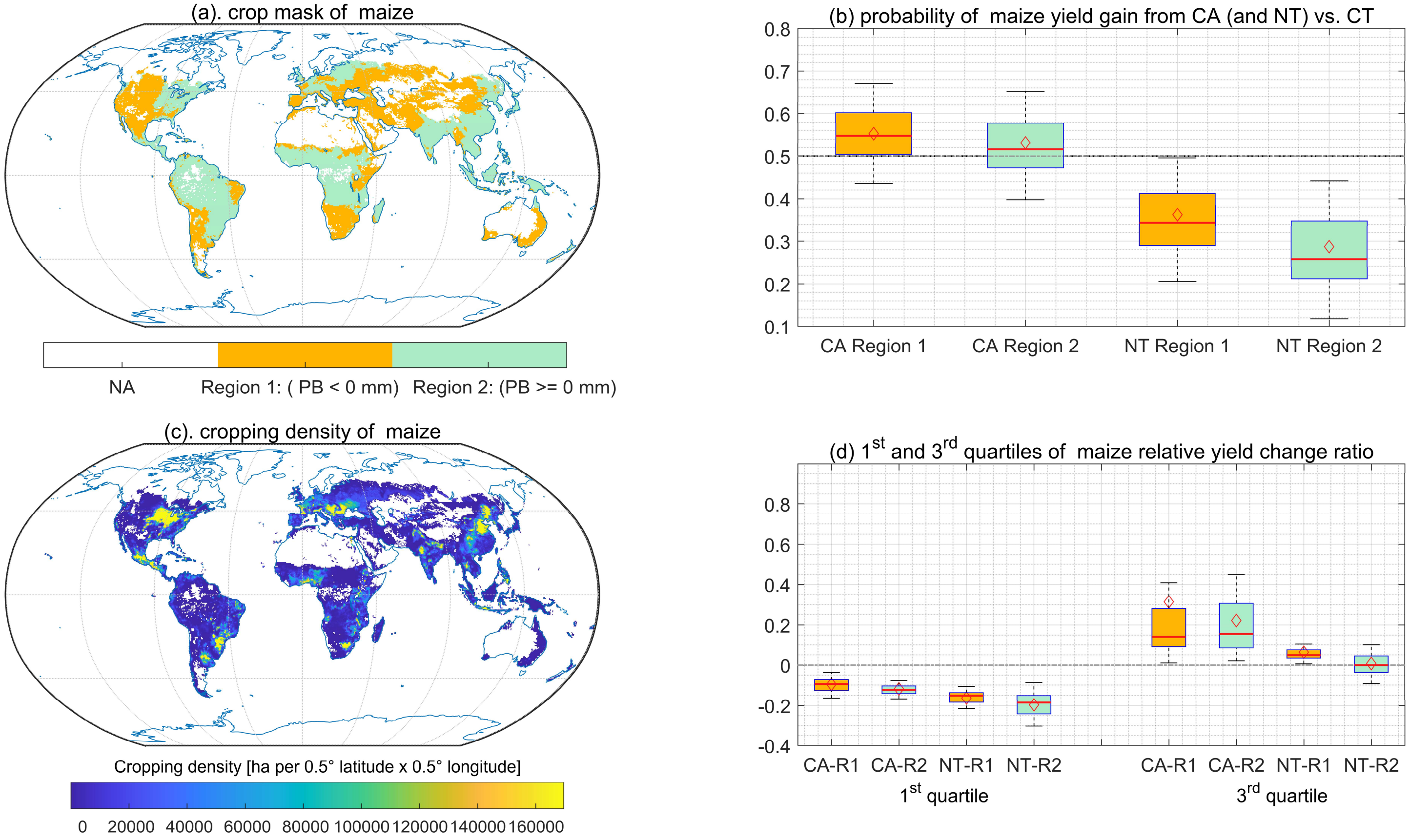

Productivity of Conservation Agriculture for maize in a relatively dry (region #1) and a relatively wet (region #2) region. (a) Illustration of the two regions on a global map with the two different colours showed in the sub-legend of plot a, while the blank area indicated the non-cropping region of maize. (b) The probability of maize yield gain (CA and NT vs. CT). (c) Maize cropping density on global map, the yellow shades indicated the higher density, and vice versa. The yellow colour in this map indicated the crop density is equal or higher than 20% of maximum density in the cell of 0.5° latitude ×0.5° longitude at the global scale. (d) The 1st and 3rd quartiles of maize relative yield change under CA and NT vs. CT practice in the two regions, and the x axis tick label in plot d: R1, R2 indicated the two different regions, and the left part of plot d indicated the yield change ratios at the 1st quartile, while the right part were the yield change ratios at 3rd quartile. The colours in plot a, b and d indicated the same regions. In plot b and d, the mean value of relative yield change in its region is marked by the red diamond, while the median value is depicted by the red horizontal line.

S15 The relative yield change of maize with CA and NT vs. CT practice

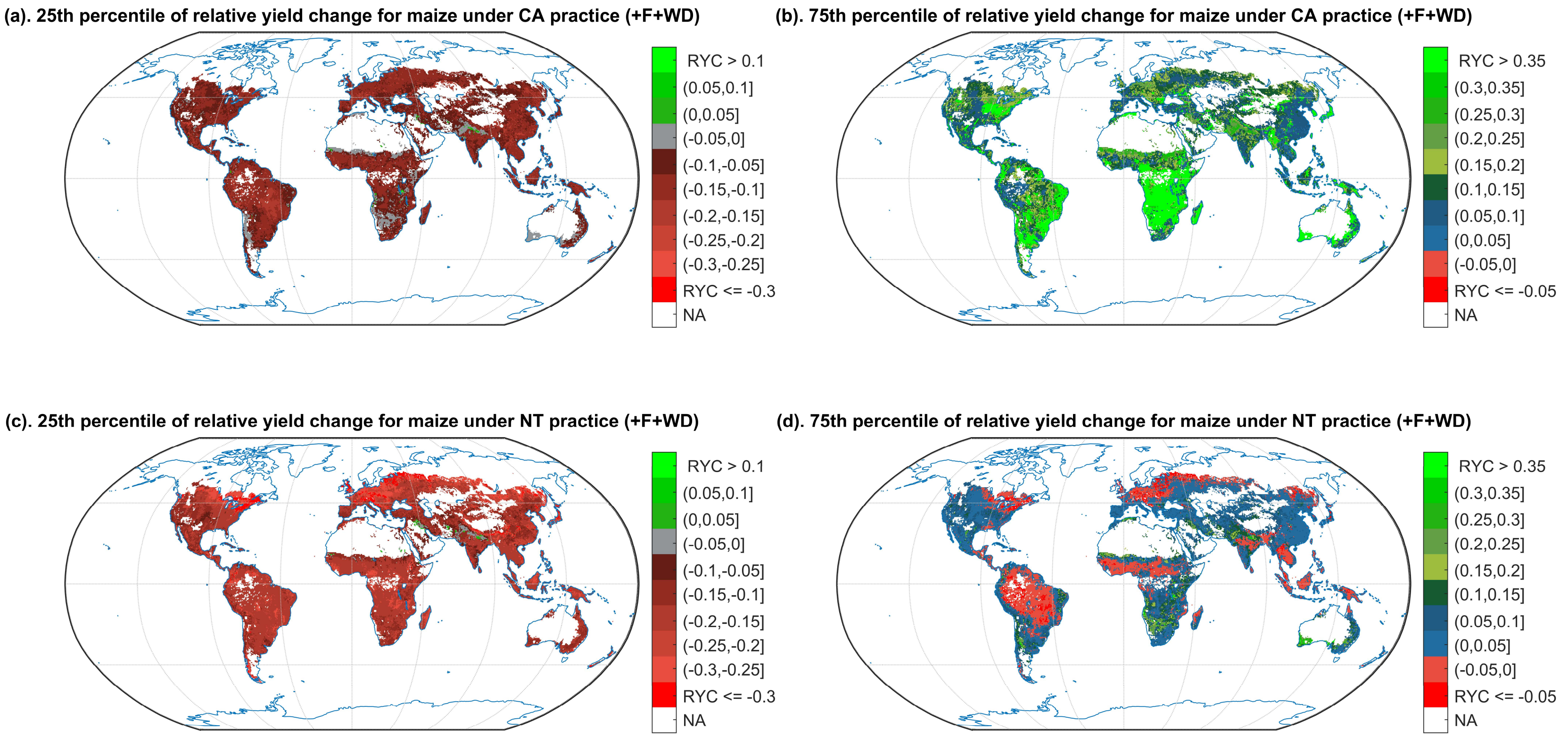

Relative yield change probability (1st and 3rd quartile estimate) of shifting CT to CA/NT for maize, with field fertilization and weed and pest control (+F+WD). There was a 75% chance that the relative yield change will be higher than the ratio shown on the map in plot a and c, and conversely a 25% chance that the relative change will be lower. There was a 75% chance that the relative yield change will be lower than the ratio shown on the map in plot b and d, and conversely a 25% chance that the relative change will be higher. The colours indicated different levels of yield change ratio, and the reddish colours indicated the negative yield change or yield loss.

S16 Productive performance of CA and NT vs. CT for rice in relatively dry and relatively wet regions

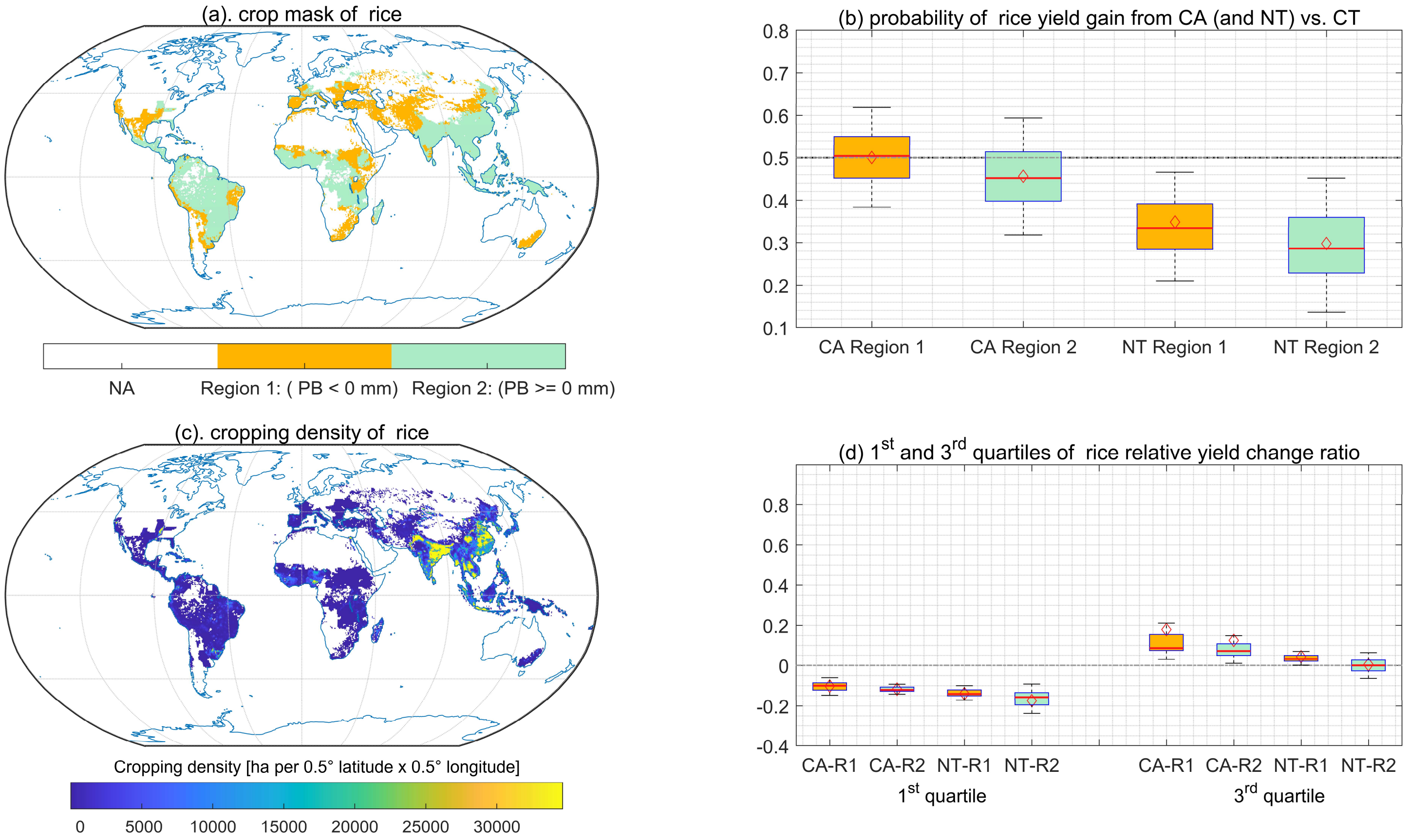

Productivity of Conservation Agriculture for rice in a relatively dry (region #1) and a relatively wet (region #2) region. (a) Illustration of the two regions on a global map with the two different colours showed in the sub-legend of plot a, while the blank area indicated the non-cropping region of rice. (b) The probability of rice yield gain (CA and NT vs. CT). (c) Rice cropping density on global map, the yellow shades indicated the higher density, and vice versa. The yellow colour in this map indicated the crop density is equal or higher than 20% of maximum density in the cell of 0.5° latitude ×0.5° longitude at the global scale. (d) The 1st and 3rd quartiles of rice relative yield change under CA and NT vs. CT practice in the two regions, and the x axis tick label in plot d: R1, R2 indicated the two different regions, and the left part of plot d indicated the yield change ratios at the 1st quartile, while the right part were the yield change ratios at 3rd quartile. The colours in plot a, b and d indicated the same regions. In plot b and d, the mean value of relative yield change in its region is marked by the red diamond, while the median value is depicted by the red horizontal line.

S17 The probability of yield gain of rice with CA and NT vs. CT practice

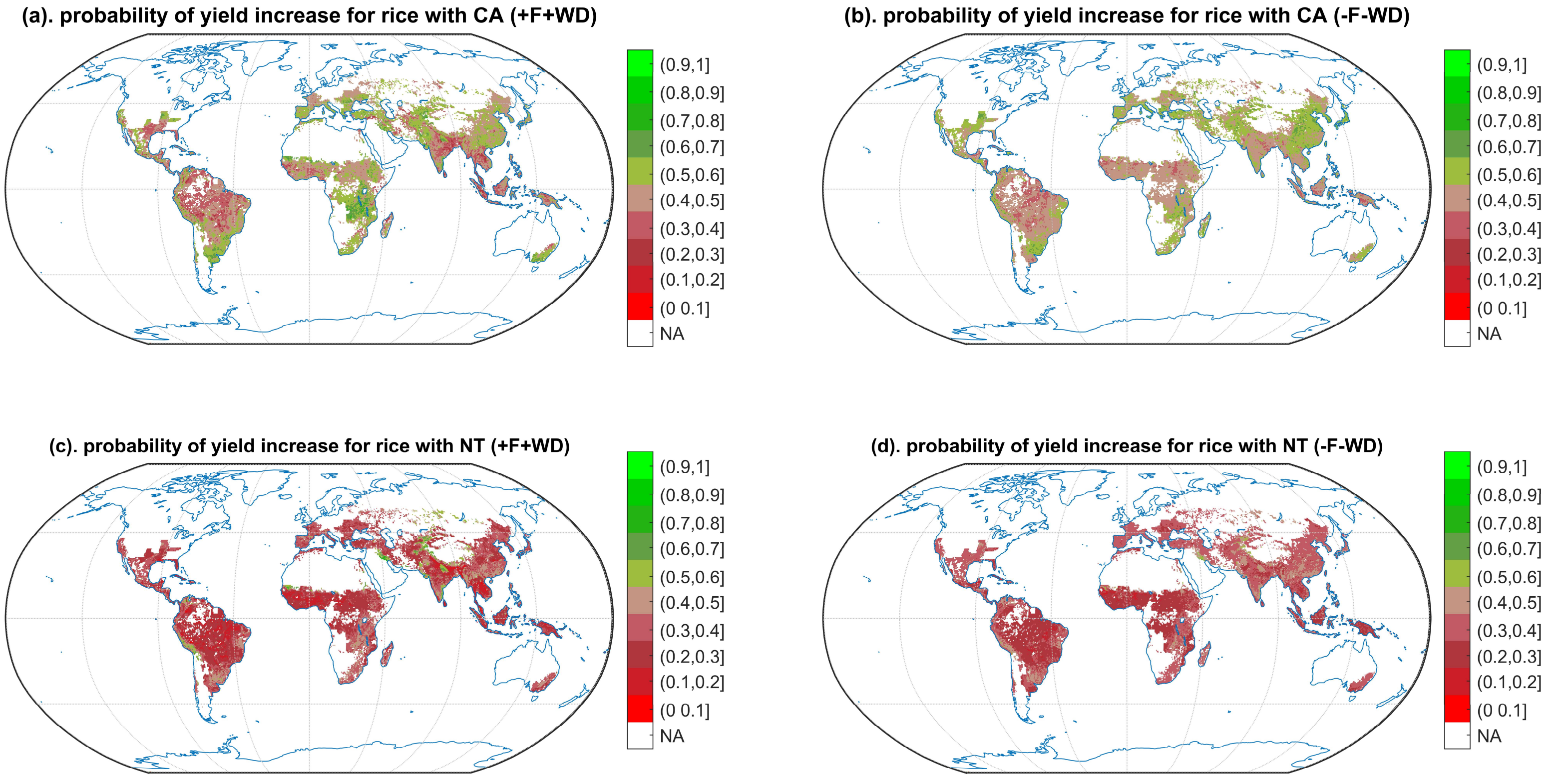

Probability of yield gain with CA and NT vs. CT rice. Only the cropping regions were presented. The different colours indicated different probabilities of yield gain from CA and NT comparing to CT system. The greener colour indicated a higher probability of yield gain. +/- F indicated NT or CA and CT with/without field fertilization. +/- WD indicated NT or CA and CT with/without weed and pest control.

S18 The relative yield change of rice with CA and NT vs. CT practice

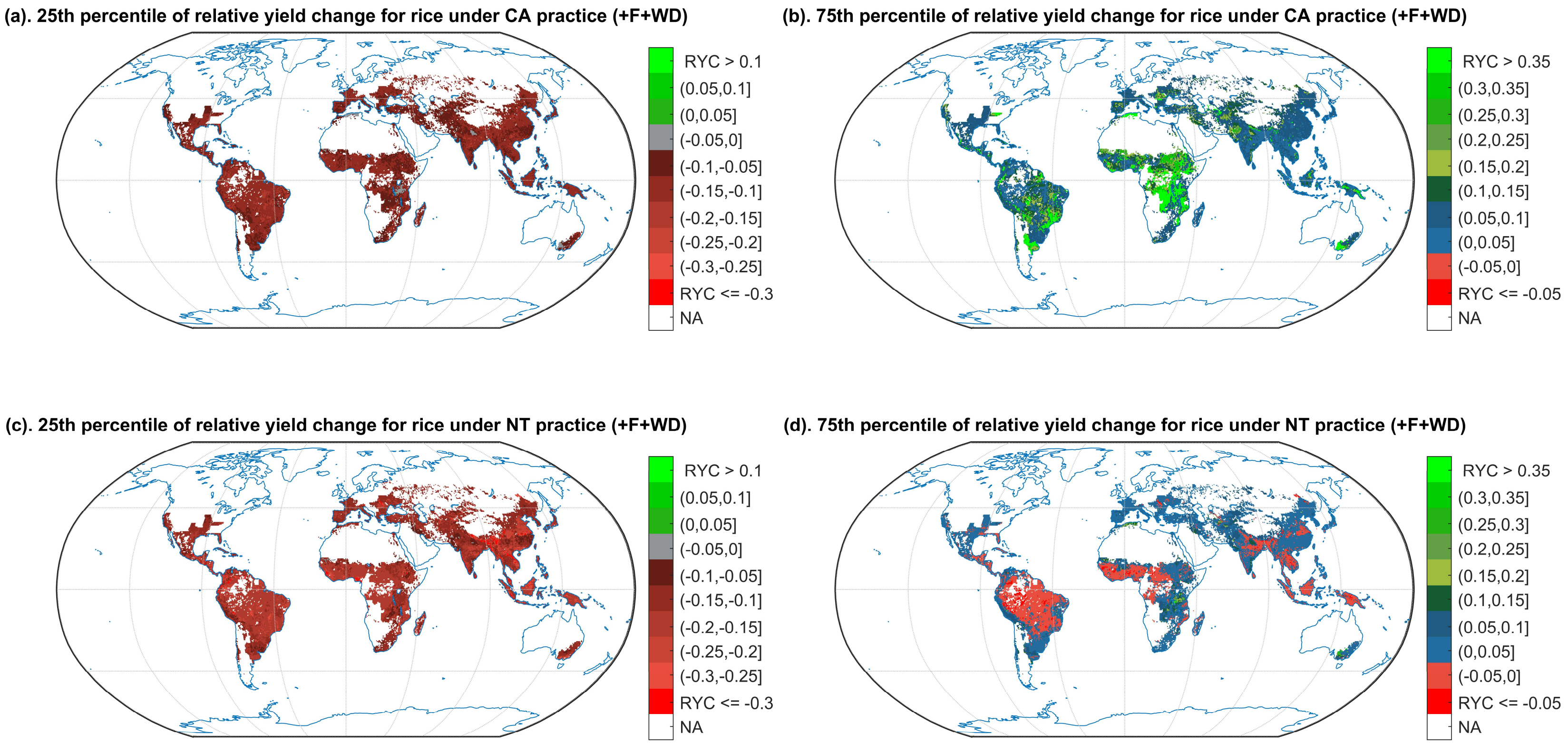

Relative yield change probability (1st and 3rd quartile estimate) of shifting CT to CA/NT for rice, with field fertilization and weed and pest control (+F+WD). There was a 75% chance that the relative yield change will be higher than the ratio shown on the map in plot a and c, and conversely a 25% chance that the relative change will be lower. There was a 75% chance that the relative yield change will be lower than the ratio shown on the map in plot b and d, and conversely a 25% chance that the relative change will be higher. The colours indicated different levels of yield change ratio, and the reddish colours indicated the negative yield change or yield loss.

S19 Productive performance of CA and NT vs. CT for sorghum in relatively dry and relatively wet regions

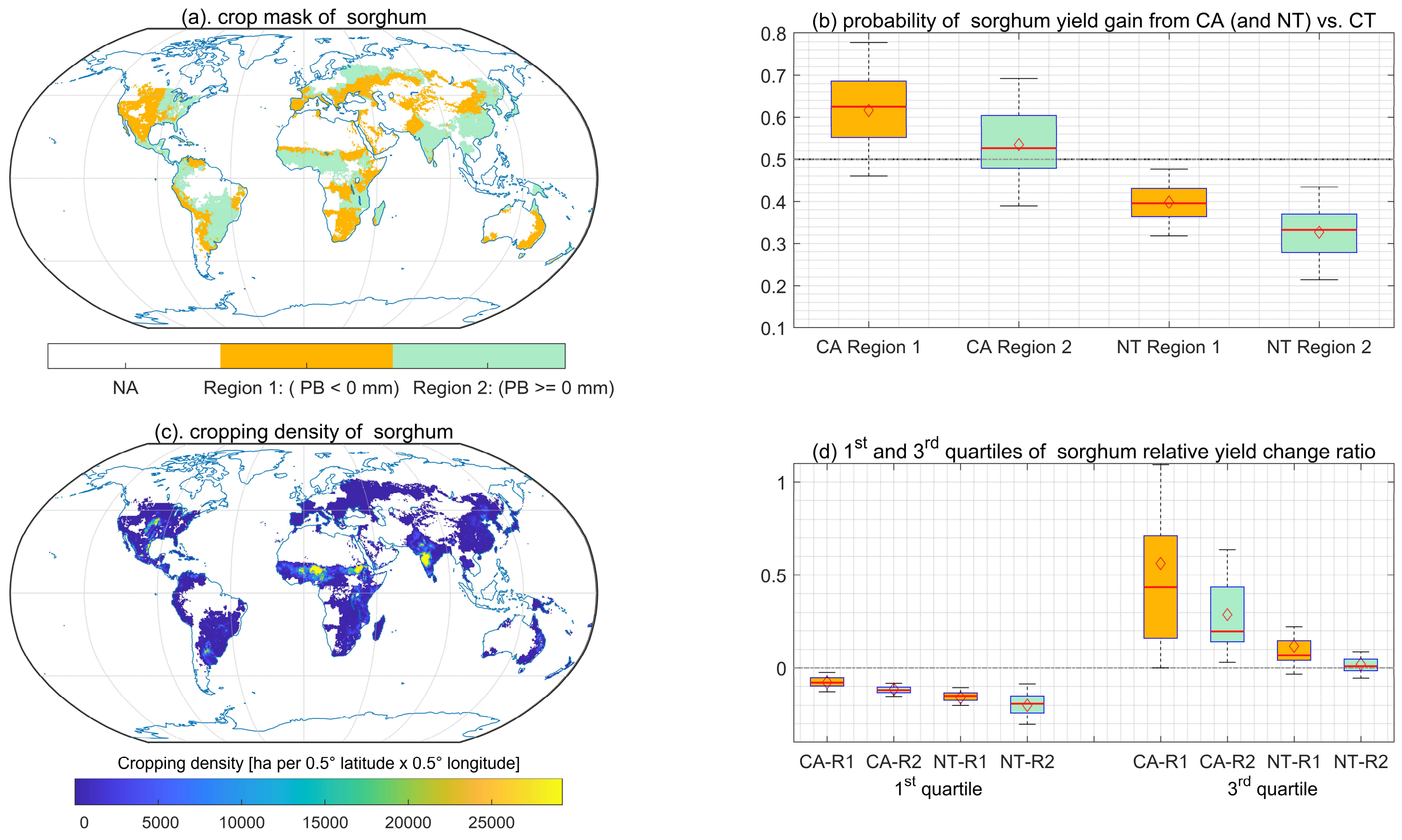

Productivity of Conservation Agriculture for sorghum in a relatively dry (region #1) and a relatively wet (region #2) region. (a) Illustration of the two regions on a global map with the two different colours showed in the sub-legend of plot a, while the blank area indicated the non-cropping region of sorghum. (b) The probability of sorghum yield gain (CA and NT vs. CT). (c) Sorghum cropping density on global map, the yellow shades indicated the higher density, and vice versa. The yellow colour in this map indicated the crop density is equal or higher than 20% of maximum density in the cell of 0.5° latitude ×0.5° longitude at the global scale. (d) The 1st and 3rd quartiles of sorghum relative yield change under CA and NT vs. CT practice in the two regions, and the x axis tick label in plot d: R1, R2 indicated the two different regions, and the left part of plot d indicated the yield change ratios at the 1st quartile, while the right part were the yield change ratios at 3rd quartile. The colours in plot a, b and d indicated the same regions. In plot b and d, the mean value of relative yield change in its region is marked by the red diamond, while the median value is depicted by the red horizontal line.

S20 The probability of yield gain of sorghum with CA and NT vs. CT practice

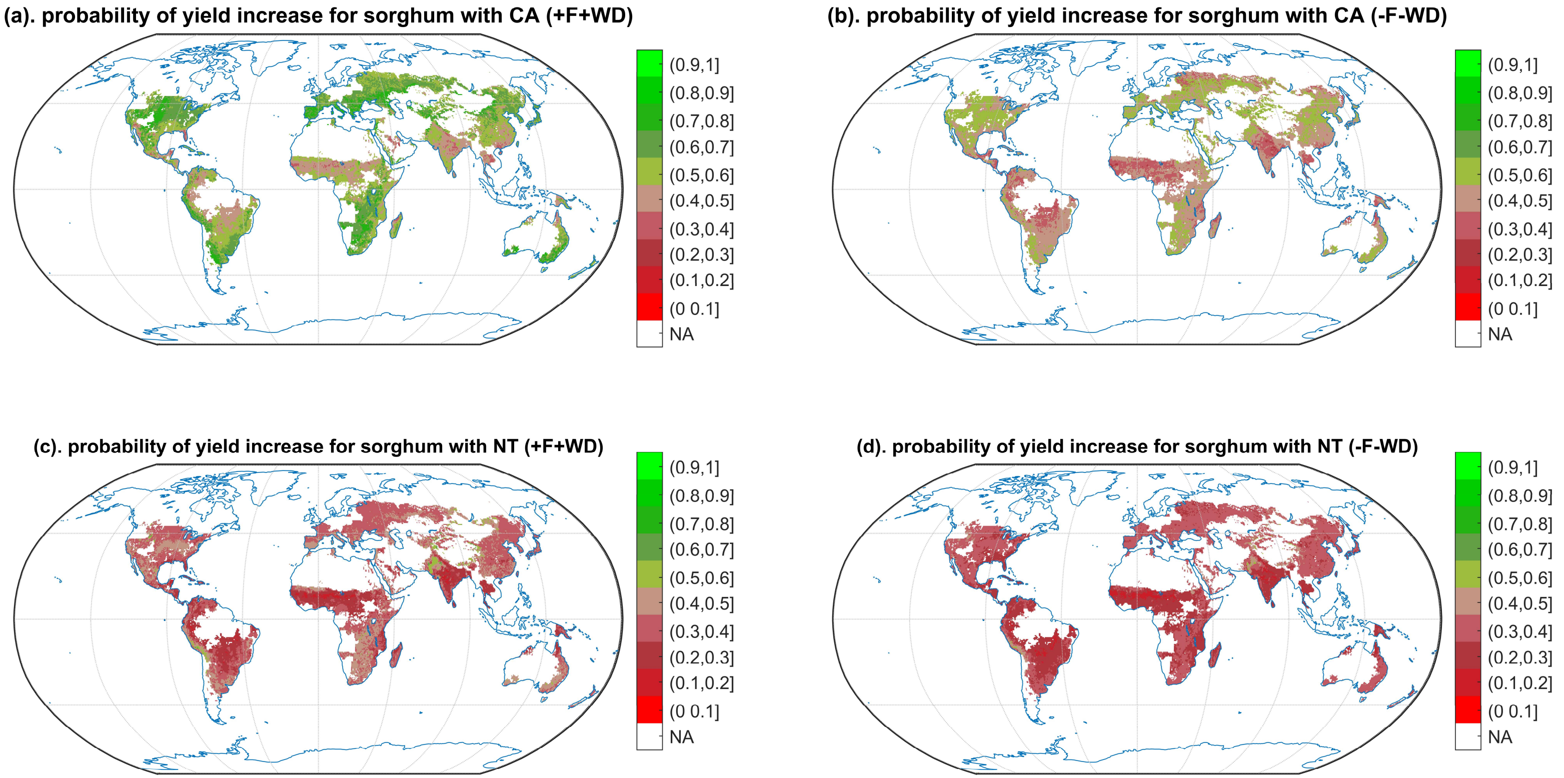

Probability of yield gain with CA and NT vs. CT sorghum. Only the cropping regions were presented. The different colours indicated different probabilities of yield gain from CA and NT comparing to CT system. The greener colour indicated a higher probability of yield gain. +/- F indicated NT or CA and CT with/without field fertilization. +/- WD indicated NT or CA and CT with/without weed and pest control.

S21 The relative yield change of sorghum with CA and NT vs. CT practice

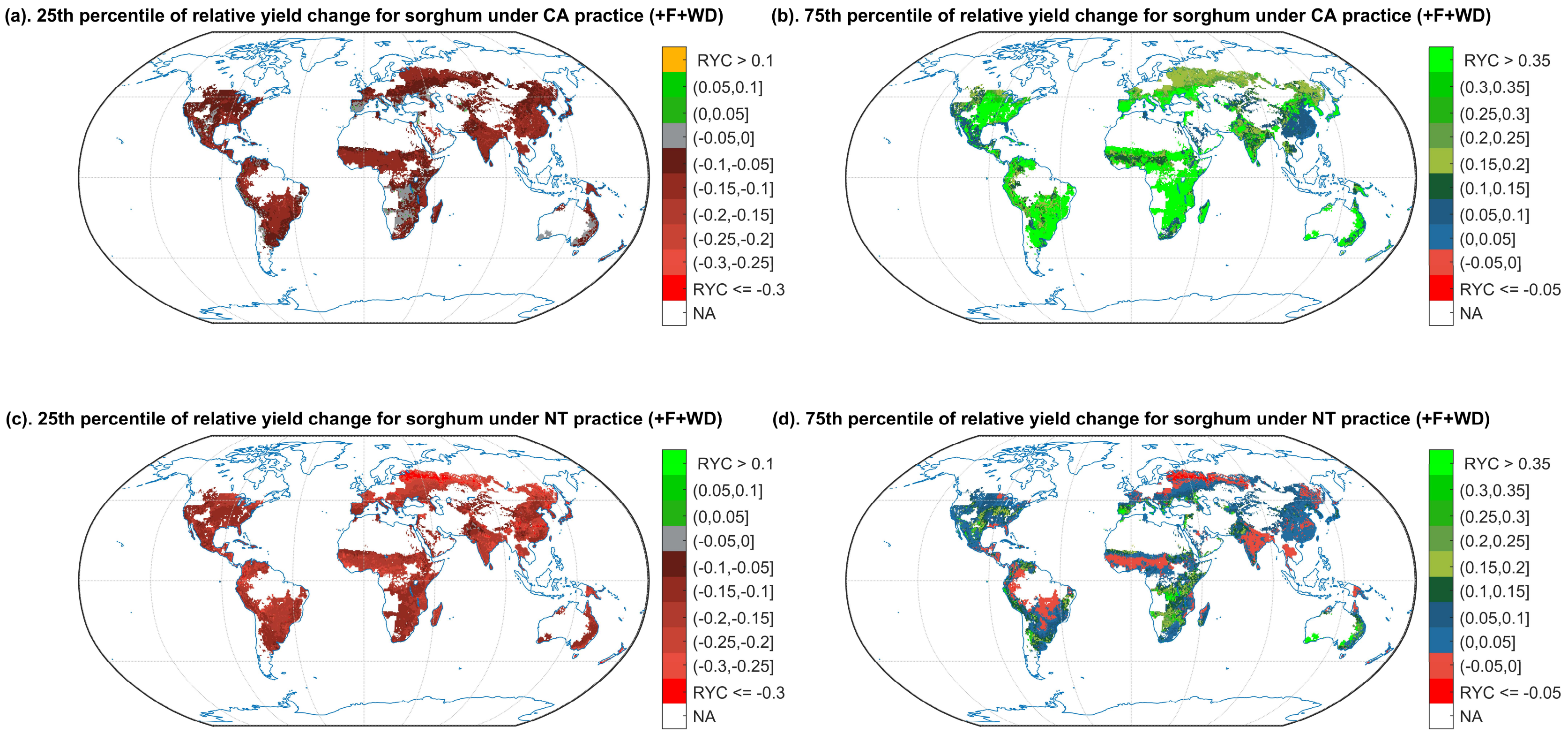

Relative yield change probability (1st and 3rd quartile estimate) of shifting CT to CA/NT for sorghum, with field fertilization and weed and pest control (+F+WD). There was a 75% chance that the relative yield change will be higher than the ratio shown on the map in plot a and c, and conversely a 25% chance that the relative change will be lower. There was a 75% chance that the relative yield change will be lower than the ratio shown on the map in plot b and d, and conversely a 25% chance that the relative change will be higher. The colours indicated different levels of yield change ratio, and the reddish colours indicated the negative yield change or yield loss.

S22 Productive performance of CA and NT vs. CT for soybean in relatively dry and relatively wet regions

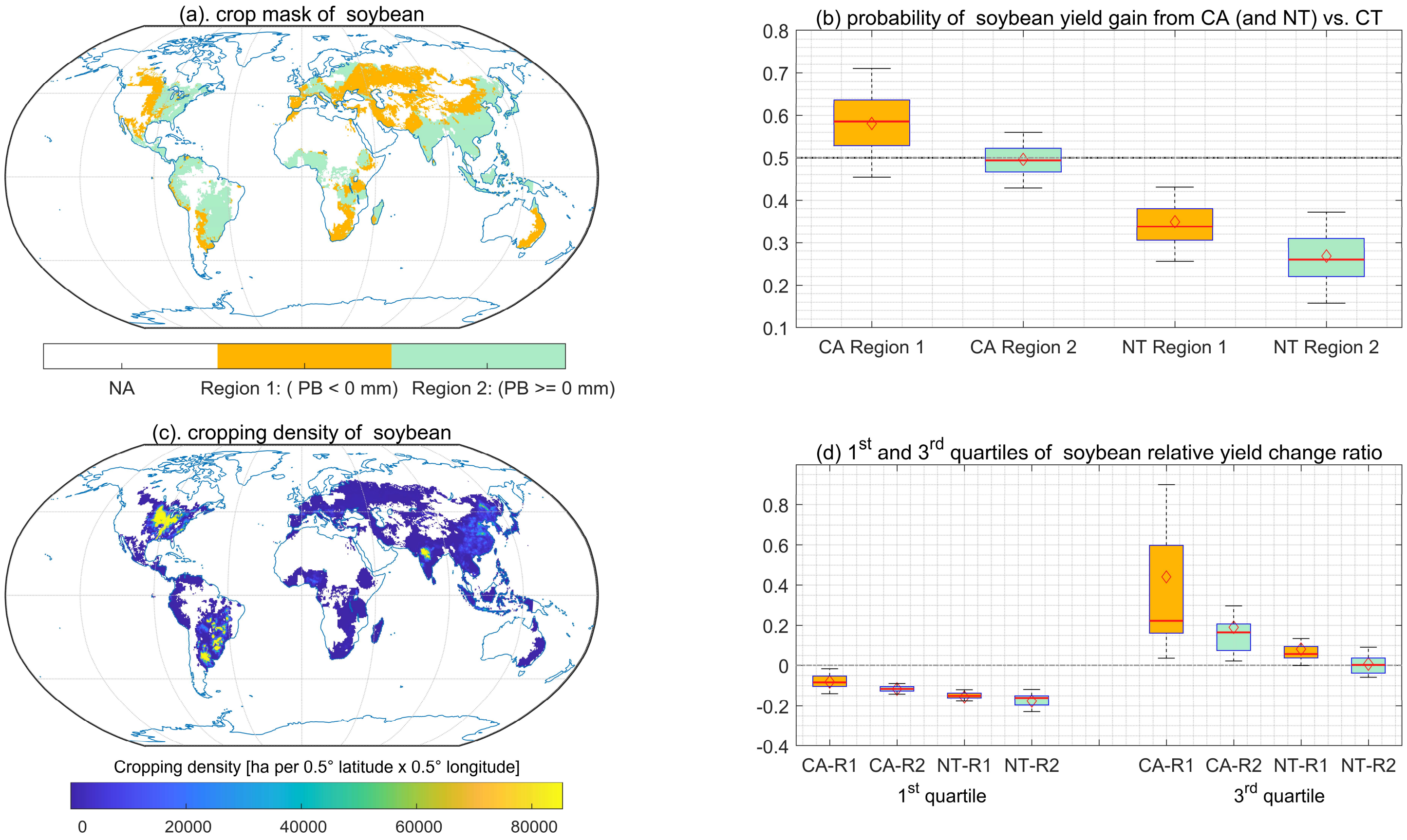

Productivity of Conservation Agriculture for soybean in a relatively dry (region #1) and a relatively wet (region #2) region. (a) Illustration of the two regions on a global map with the two different colours showed in the sub-legend of plot a, while the blank area indicated the non-cropping region of soybean. (b) The probability of soybean yield gain (CA and NT vs. CT). (c) Soybean cropping density on global map, the yellow shades indicated the higher density, and vice versa. The yellow colour in this map indicated the crop density is equal or higher than 20% of maximum density in the cell of 0.5° latitude ×0.5° longitude at the global scale. (d) The 1st and 3rd quartiles of soybean relative yield change under CA and NT vs. CT practice in the two regions, and the x axis tick label in plot d: R1, R2 indicated the two different regions, and the left part of plot d indicated the yield change ratios at the 1st quartile, while the right part were the yield change ratios at 3rd quartile. The colours in plot a, b and d indicated the same regions. In plot b and d, the mean value of relative yield change in its region is marked by the red diamond, while the median value is depicted by the red horizontal line.

S23 The probability of yield gain of soybean with CA and NT vs. CT practice

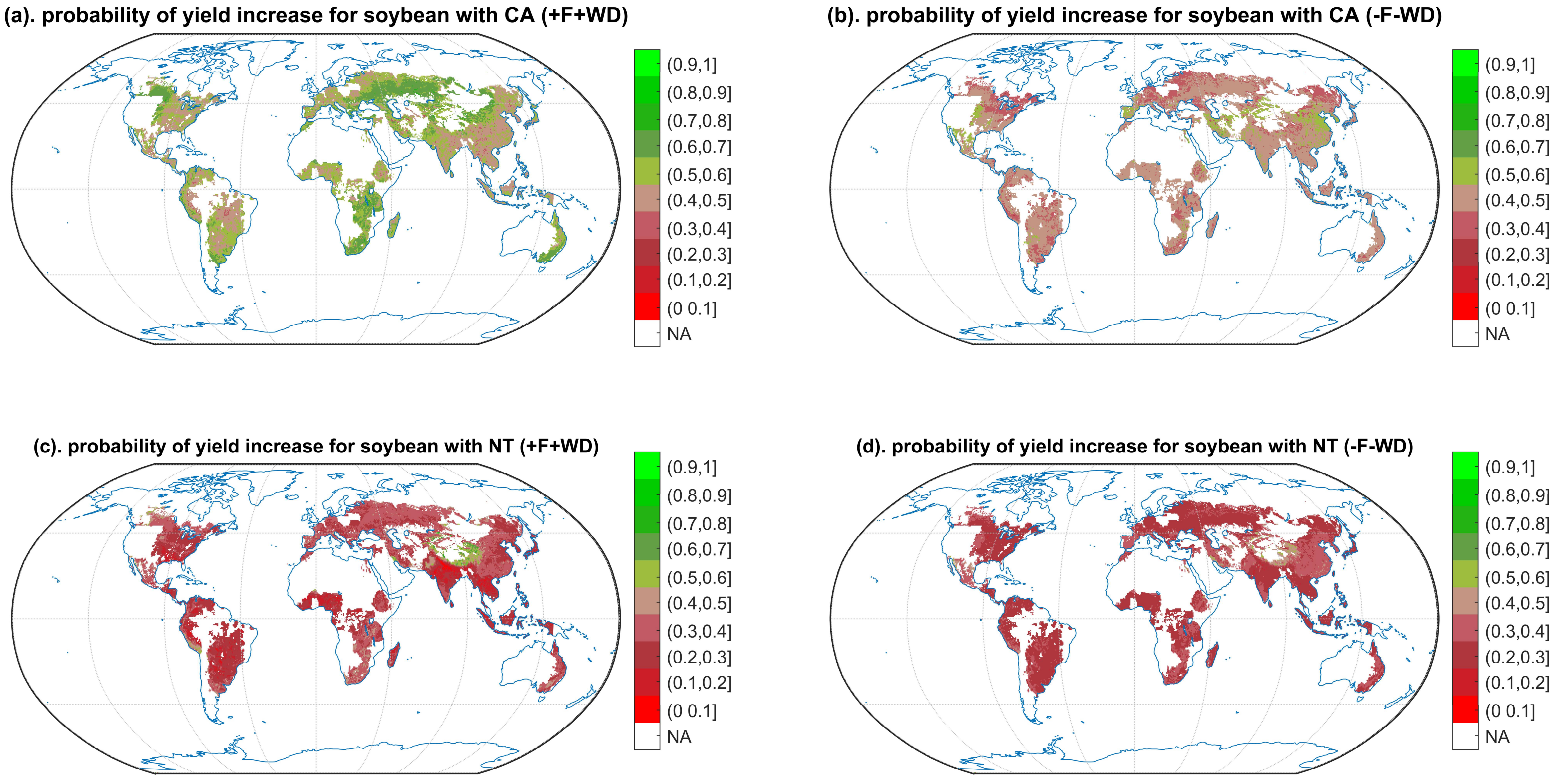

Probability of yield gain with CA and NT vs. CT soybean. Only the cropping regions were presented. The different colours indicated different probabilities of yield gain from CA and NT comparing to CT system. The greener colour indicated a higher probability of yield gain. +/- F indicated NT or CA and CT with/without field fertilization. +/- WD indicated NT or CA and CT with/without weed and pest control.

S24 The relative yield change of soybean with CA and NT vs. CT practice

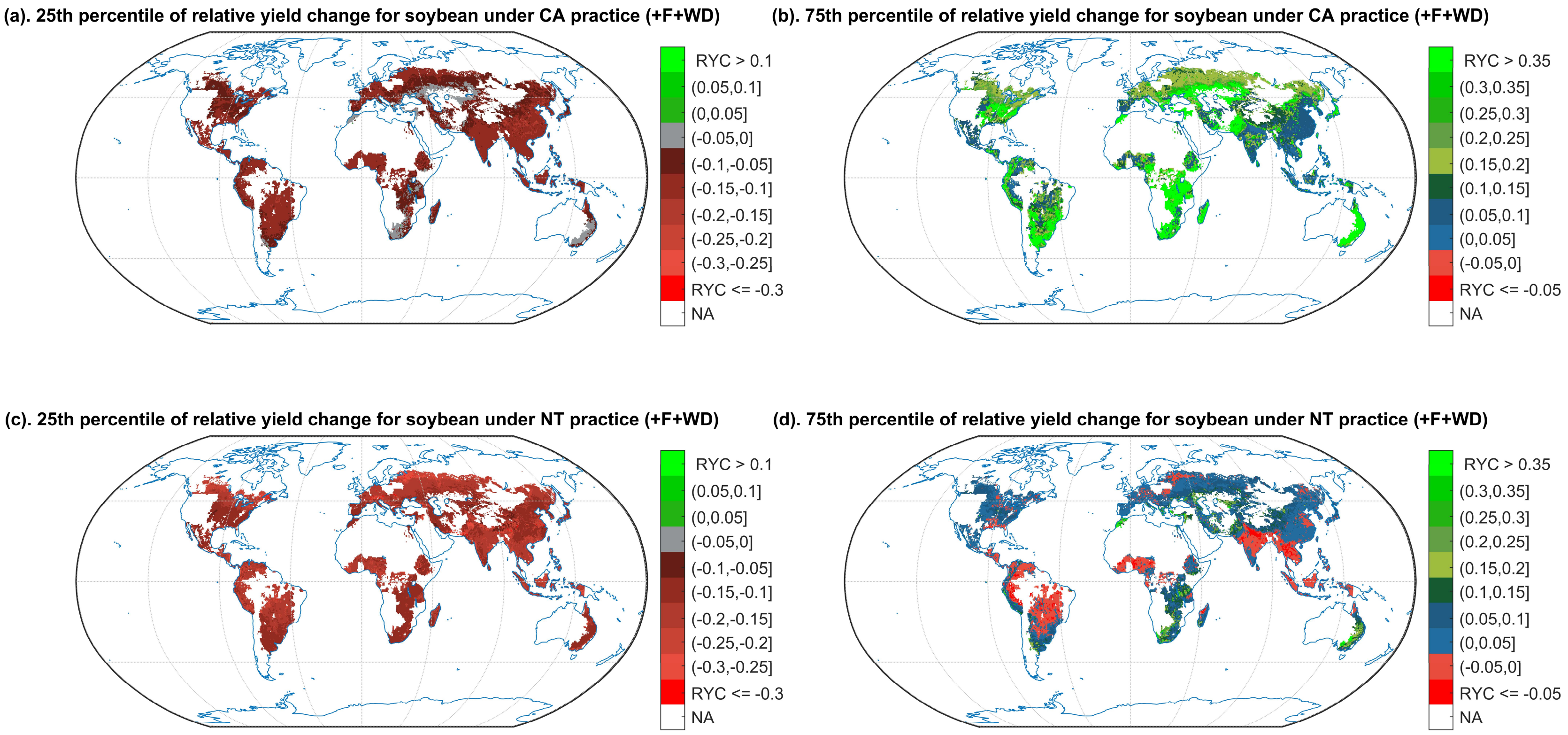

Relative yield change probability (1st and 3rd quartile estimate) of shifting CT to CA/NT for soybean, with field fertilization and weed and pest control (+F+WD). There was a 75% chance that the relative yield change will be higher than the ratio shown on the map in plot a and c, and conversely a 25% chance that the relative change will be lower. There was a 75% chance that the relative yield change will be lower than the ratio shown on the map in plot b and d, and conversely a 25% chance that the relative change will be higher. The colours indicated different levels of yield change ratio, and the reddish colours indicated the negative yield change or yield loss.

S25 Productive performance of CA and NT vs. CT for sunflower in relatively dry and relatively wet regions

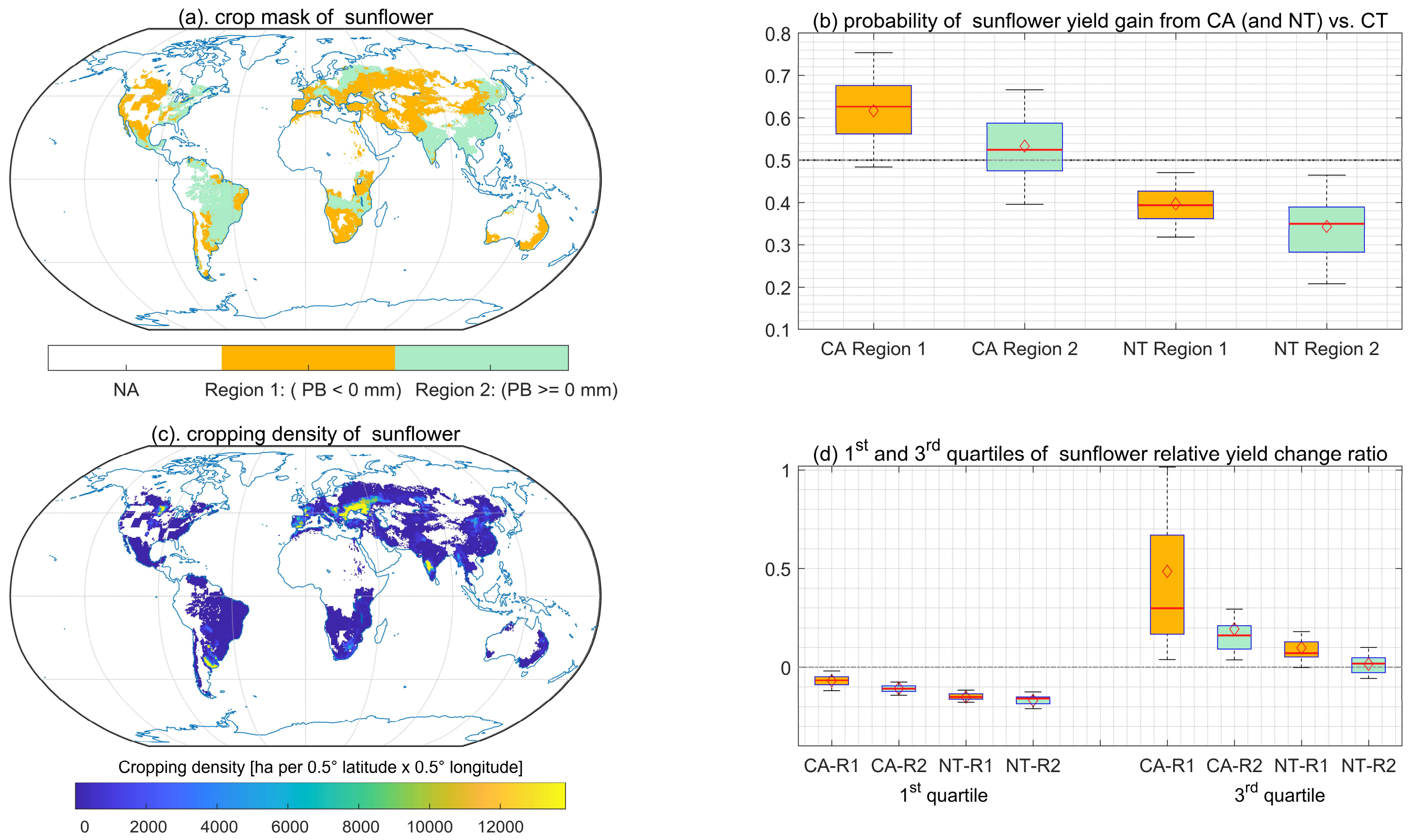

Productivity of Conservation Agriculture for sunflower in a relatively dry (region #1) and a relatively wet (region #2) region. (a) Illustration of the two regions on a global map with the two different colours showed in the sub-legend of plot a, while the blank area indicated the non-cropping region of sunflower. (b) The probability of sunflower yield gain (CA and NT vs. CT). (c) Sunflower cropping density on global map, the yellow shades indicated the higher density, and vice versa. The yellow colour in this map indicated the crop density is equal or higher than 20% of maximum density in the cell of 0.5° latitude ×0.5° longitude at the global scale. (d) The 1st and 3rd quartiles of sunflower relative yield change under CA and NT vs. CT practice in the two regions, and the x axis tick label in plot d: R1, R2 indicated the two different regions, and the left part of plot d indicated the yield change ratios at the 1st quartile, while the right part were the yield change ratios at 3rd quartile. The colours in plot a, b and d indicated the same regions. In plot b and d, the mean value of relative yield change in its region is marked by the red diamond, while the median value is depicted by the red horizontal line.

S26 The probability of yield gain of sunflower with CA and NT vs. CT practice

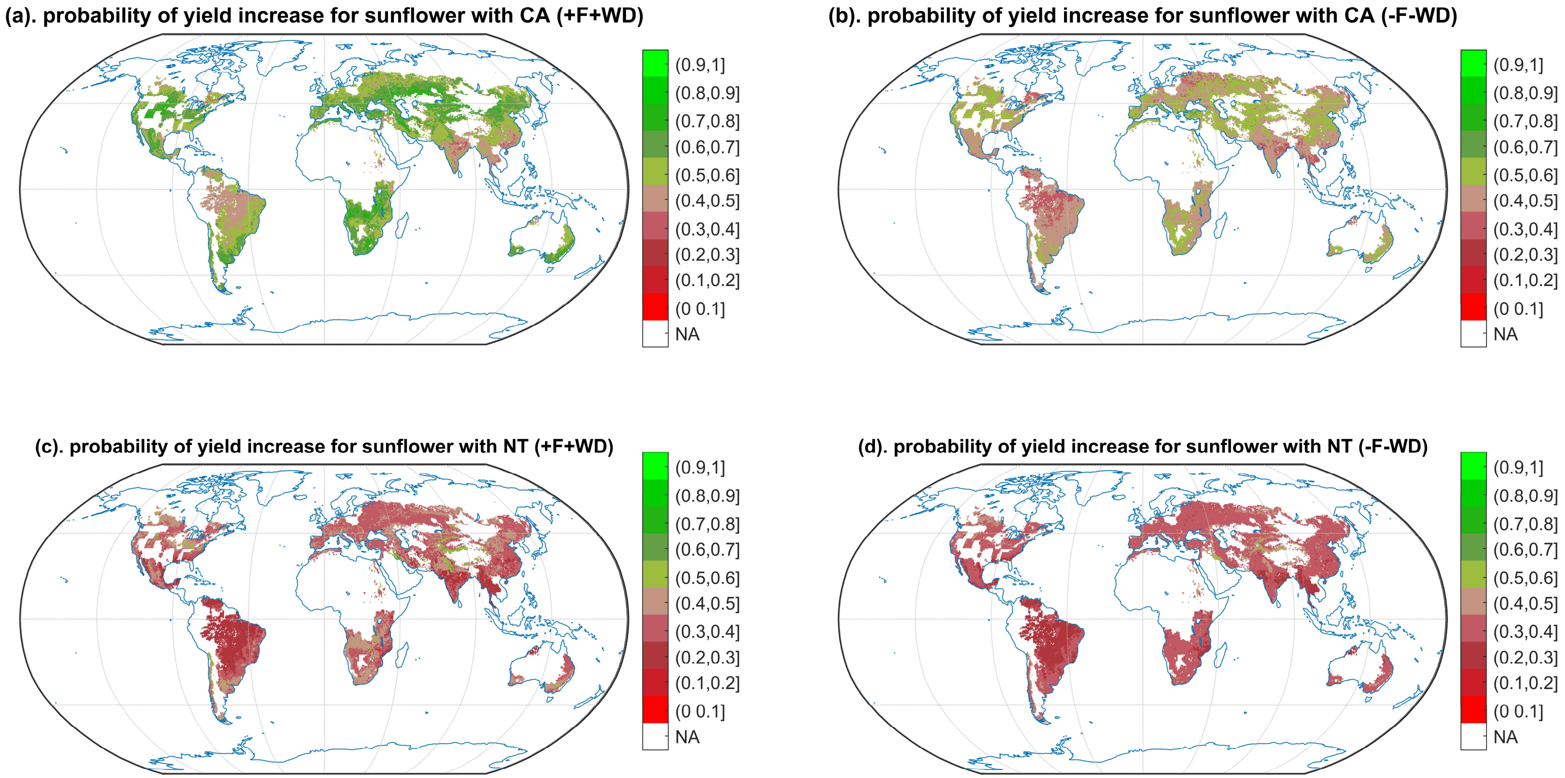

Probability of yield gain with CA and NT vs. CT sunflower. Only the cropping regions were presented. The different colours indicated different probabilities of yield gain from CA and NT comparing to CT system. The greener colour indicated a higher probability of yield gain. +/- F indicated NT or CA and CT with/without field fertilization. +/- WD indicated NT or CA and CT with/without weed and pest control.

S27 The relative yield change of sunflower with CA and NT vs. CT practice

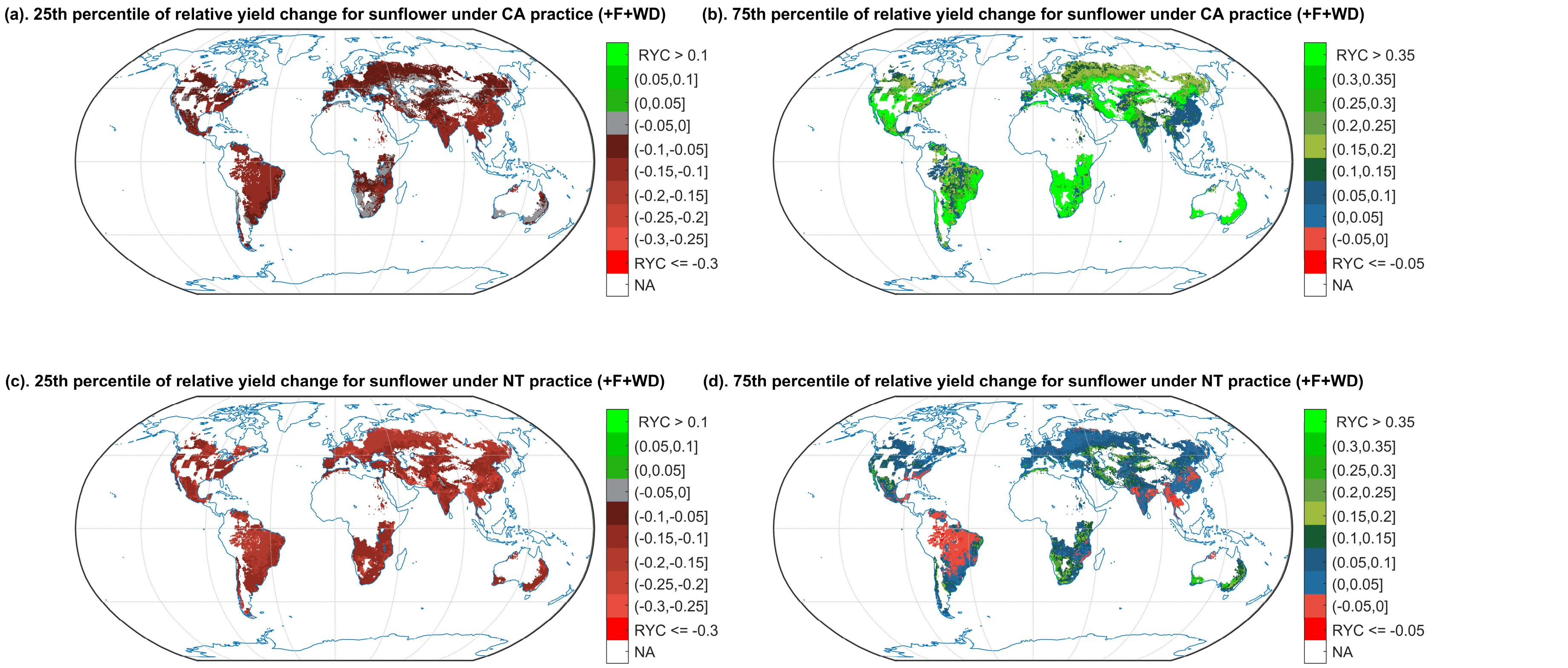

Relative yield change probability (1st and 3rd quartile estimate) of shifting CT to CA/NT for sunflower, with field fertilization and weed and pest control (+F+WD). There was a 75% chance that the relative yield change will be higher than the ratio shown on the map in plot a and c, and conversely a 25% chance that the relative change will be lower. There was a 75% chance that the relative yield change will be lower than the ratio shown on the map in plot b and d, and conversely a 25% chance that the relative change will be higher. The colours indicated different levels of yield change ratio, and the reddish colours indicated the negative yield change or yield loss.

S28 Methods

Database establishment

The literature search was done in February 2020 using the following keywords ‘Conservation agriculture / No-till / No tillage / Zero tillage’ & ‘Yield / Yield change’ in the websites ‘ScienceDirect’, ‘Science Citation Index (web of science)’. A total of 1012 potentially relevant papers were identified by reviewing the title and abstract, and these papers were then screened according to the procedure summarized in S1. Papers not reporting yield data for CT and NT systems were excluded, as well as papers reporting experiments on reduced tillage (RT) systems. Papers reporting only mean yield data across different years or sites were also excluded. We then checked whether information on fertilization, weed and pest control, crop irrigation, crop rotation (at least three crops involved based on the definition of CA by FAO [11]) and crop residue management were reported for both CT and NT practices. After these screening and selection steps, all relevant data were manually extracted from the selected papers, including general information about the paper, location and year of the experiment, the number of years under NT when the crop was sown, soil characteristics, crop growing season, crop type, crop management practices and crop yield of CT and NT. However, due to a large number of missing data, the crop growing season, climatic variables and soil characteristics were finally collected through several external databases. The growing season information was generated from a crop calendar database [3] based on the crop type and the locations of the experiments reported in the papers. The precipitation, average temperature in the growing season were extracted from the UDel_AirT_Precip data provided by NOAA/OAR/ESRL PSL[12]. The maximum and minimum air temperature during the growing season were generated from CPC Global Temperature data provided by NOAA/OAR/ESRL PSL [6] and the potential evapotranspiration data over the growing season were extracted from GLEAM database[12]. Soil textures were collected from the HWSD database [7] using the locations of the experimental sites reported in the selected papers. The experiments for which it was not possible to obtain the requested information from the external databases were excluded. The final dataset includes the results extracted from 413 papers (published between 1983 to 2020), 4403 paired yield observations from NT and CT for 8 major crop species, including 370 observations for barley (232 observations for spring barley and 138 for winter barley), 94 observations for cotton, 1690 observations for maize, 195 observation for rice, 160 observations for sorghum, 583 observations for soybean, 61 observations for sunflower, 1250 observations for wheat (1041 observations for winter wheat and 209 observations for spring wheat) in 50 countries from 1980 to 2017 (S2).

Analysis

Pairs of yield data were used to compute two types of effect size, namely yield ratio $\left(\frac{Yiel_{CA\ or\ NT}}{Yie_{CT}}\right)$ and relative yield change ratio $\left(\frac{Yield_{CA\ or\ NT} - Yield_{CT}}{Yield_{CT}}\right)$. The category of the yield gain and loss were then related to the inputs of climatic conditions, soil data, and agricultural management practices using a random forest algorithm in order to map the probability of yield gain with CA and NT vs. CT (probability that yield ratio > 1). Relative yield changes were related to the same input data using quantile regression forest to estimate the 25th and 75th percentiles of relative yield change in different climatic conditions.

The R package “randomForest” (version 4.6 - 14) was used to train the random forest model to analyse the yield ratios of CA or NT vs. CT as the function of climatic variables, crop types, soil textures, and agricultural management practices. To do so, set the climatic variables throughout the growing season such as PB, Tmin/Tave/Tmax as numerical explanatory variables; Set crop type, soil texture, and agricultural management activities including crop irrigation, field fertilization, control of pests and weeds, crop rotation and soil cover as categorical explanatory variables; Set the category of yield change (yield ratio $\left(\frac{Yield_{CA}}{Yield_{CT}}\right)$ >1 or <=1) as response variable. The sample code for training the random forest

model is available in "Code.zip" in figshare repository provided in the main manuscript. When setting the "proximity" as Ture, the model output is the probability of yield gain from NT vs. CT. As for the quantile regression model, the R package "ranger" (version 0.11.2) was used to train a quantile regression forest model to analyse relative yield change of shifting from CT to CA or NT as the same explanatory variables as random forest. Both random forest model and quantile regression forest model were tree-based ensemble machine learning method using multiple deep decision trees to improve the accuracy and stability of their predictions[12]. These methods did not make assumptions on the distributions of the input data and were able to deal with high-dimensional data. The importance ranking of the inputs were derived using the mean decrease of Gini impurity index [13](results available in S7).

Global projection of yield gain probability and relative yield change ratio

To predict the global productivity of CA and NT vs. CT, the trained machine learning models were fed by the numerical explanatory variables, such as climatic variables, and categorical explanatory variables, including crop types, soil textures, agricultural management practices. The details of the model input settings were available in S5. The global climatic variables were based on the latest NCEI's three-decade averaged climatic conditions[14], also called "1981-2010 U.S. Climate Normals". The growing season information for each crop was defined as the period between seeding month and harvesting month from the crop calendar database[8,9]. Mean precipitation and average temperature were calculated throughout the growing season for all the years from 1981 to 2010 using the global precipitation and air temperature database[3]. A similar approach was implemented for maximum and minimum temperature[6], and potential evapotranspiration[4,5]. As for the categorical explanatory variables, the global soil texture input data came from HWSD database[15], the crop irrigation input data was based on the irrigation mask from MIRCA2000 database[10], in each grid cell, crop irrigation was marked as yes if irrigation was the dominated practices in this region. Crop rotation and soil cover were set as "yes" for CA, and "no" for NT. Crop fertilization and integrated weed and pest control were set as "yes" for the scenario (+F+WD), while "no" for the scenario (-F-WD).

The model outputs were the probabilities of yield gain with CA or NT vs. CT, and 25th and 75th percentiles of relative yield change, which indicated the CA or NT productivity at the global scale under the averaged climate condition of 1981-2010. All maps in the paper were generated by MATLAB R2020a (Version 9.8.0.1451342, https://fr.mathworks.com/products/matlab.html).

S29 Reference for meta-analysis

1. Aase, J. K., Schaefer, G. M. & Pikul, J. L. Hayland conversion to wheat production in semiarid eastern Montana: Tillage, yield and hay production comparisons. *Soil and Tillage Research* **44**, 225–234 (1997).

2. Abidela Hussein, M. *et al.* Deep tillage improves degraded soils in the (sub) humid Ethiopian highlands. *Land* **8**, 159 (2019).

3. Acharya, B. S. *et al.* Winter cover crops effect on soil moisture and soybean growth and yield under different tillage systems. *Soil and Tillage Research* **195**, 104430 (2019).

4. Adimassu, Z., Alemu, G. & Tamene, L. Effects of tillage and crop residue management on runoff, soil loss and crop yield in the Humid Highlands of Ethiopia. *Agricultural Systems* **168**, 11–18 (2019).

5. Ahmed, W. *et al.* Tillage practices improve rice yield and soil phosphorus fractions in two typical paddy soils. *Journal of Soils and Sediments* **20**, 850–861 (2020).

6. Ahrens, W. H. & Endres, G. J. Trifluralin and ethalfluralin granules in conservation-tillage soybeans (Glycine max). *Canadian Journal of Plant Science* **76**, 891–897 (1996).

7. Alam, M. *et al.* Banding of fertilizer improves phosphorus acquisition and yield of zero tillage maize by concentrating phosphorus in surface soil. *Sustainability* **10**, 3234 (2018).

8. Ali, S., Tedone, L., Verdini, L., Cazzato, E. & De Mastro, G. Wheat response to no-tillage and nitrogen fertilization in a long-term faba bean-based rotation. *Agronomy* **9**, 50 (2019).

9. Almaraz, J. J. *et al.* Greenhouse gas fluxes associated with soybean production under two tillage systems in southwestern Quebec. *Soil and Tillage Research* **104**, 134–139 (2009).

10. Almaraz, J. J. *et al.* Carbon Dioxide and Nitrous Oxide Fluxes in Corn Grown under Two Tillage Systems in Southwestern Quebec. *Soil Science Society of America Journal* **73**, 113–119 (2009).

11. Anapalli, S. S. *et al.* Effectiveness of RZWQM for simulating alternative great plains cropping systems. *Agronomy Journal* **97**, 1183–1193 (2005).

12. Andales, A. A., Batchelor, W. D., Anderson, C. E., Farnham, D. E. & Whigham, D. K. Incorporating tillage effects into a soybean model. *Agricultural Systems* **66**, 69–98 (2000).

13. Anderson, R. L. A 2-year small grain interval reduces need for herbicides in no-till soybean. *Weed Technology* **23**, 398–403 (2009).

14. Angás, P., Lampurlanés, J. & Cantero-Martínez, C. Tillage and N fertilization: Effects on N dynamics and Barley yield under semiarid Mediterranean conditions. *Soil and Tillage Research* **87**, 59–71 (2006).

15. Armstrong, R. D., Millar, G., Halpin, N. V., Reid, D. J. & Standley, J. Using zero tillage, fertilisers and legume rotations to maintain productivity and soil fertility in opportunity cropping systems on a shallow Vertosol. *Australian Journal of Experimental Agriculture* **43**, 141–153 (2003).

16. Arora, V. K., Sidhu, A. S., Sandhu, K. S. & Thind, S. S. Effects of tillage intensity, planting time and nitrogen rate on wheat yield following rice. *Experimental Agriculture* **46**, 267–275 (2010).

17. Arshad, M. A. & Gill, K. S. Barley, canola and wheat production under different tillage-fallow-green manure combinations on a clay soil in a cold, semiarid climate. *Soil and Tillage Research* **43**, 263–275 (1997).

18. Arshad, M. A., Gill, K. S. & Coy, G. R. Wheat yield and weed population as influenced by three tillage systems on a clay soil in temperate continental climate. *Soil and Tillage Research* **28**, 227–238 (1994).

19. Arshad, M. A., Gill, K. S. & Izaurralde, R. C. Wheat Production, Weed Population and Soil Properties Subsequent to 20 Years of Sod as Affected by Crop Rotation and Tillage. *Journal of Sustainable Agriculture* **12**, 131–154 (1998).

20. Arvidsson, J. Energy use efficiency in different tillage systems for winter wheat on a clay and silt loam in Sweden. *European Journal of Agronomy* **33**, 250–256 (2010).

21. Aulakh, M. S. *et al.* Crop production and nutrient use efficiency of conservation agriculture for soybean-wheat rotation in the Indo-Gangetic Plains of Northwestern India. *Soil and Tillage Research* **120**, 50–60 (2012).

22. Azooz, R. H. & Arshad, M. A. Effect of tillage and residue management on barley and canola growth and water use efficiency. *Canadian Journal of Soil Science* **78**, 649–656 (1998).

23. Baan, C. D., Grevers, M. C. J. & Schoenau, J. J. Effects of a single cycle of tillage on long-term no-till prairie soils. *Canadian Journal of Soil Science* **89**, 521–530 (2009).

24. Baggs, E. M. *et al.* Nitrous oxide emissions following application of residues and fertiliser under zero and conventional tillage. *Plant and Soil* **254**, 361–370 (2003).

25. Baghdadi, A., Halim, R. A., Majidian, M., Wan Daud, W. N. & Ahmad, I. Forage corn yield and physiological indices under different plant densities and tillage systems. *Journal of Food, Agriculture and Environment* **10**, 707–712 (2012).

26. Bajpai, R. K. & Tripathi, R. P. Evaluation of non-puddling under shallow water tables and alternative tillage methods on soil and crop parameters in a rice–wheat system in Uttar Pradesh. *Soil and Tillage Research* **55**, 99–106 (2000).

27. Balkcom, K. S., Reeves, D. W., Kemble, J. M., Dawkins, R. A. & Raper, R. L. Tillage requirements of sweet corn, field pea, and watermelon following stocker cattle grazing. *Journal of Sustainable Agriculture* **34**, 169–182 (2010).

28. Balkcom, K. S. *et al.* Row spacing, tillage system, and herbicide technology affects cotton plant growth and yield. *Field Crops Research* **117**, 219–225 (2010).

29. Ball, B. C., O'Sullivan, M. F. & Lang, R. W. Cultivation and nitrogen requirement for winter barley as assessed from a reduced-tillage experiment on a brown forest soil. *Soil and Tillage Research* **6**, 95–109 (1985).

30. Barber, R. G., Orellana, M., Navarro, F., Diaz, O. & Soruco, M. A. Effects of conservation and conventional tillage systems after land clearing on soil properties and crop yield in Santa Cruz, Bolivia. *Soil and Tillage Research* **38**, 133–152 (1996).

31. Barrios, M. B., Bozzo, A. A., Debelis, S. P., Pereyra, A. M. & Buján, A. Soil physical properties and root activity in a soybean second crop/maize rotation under direct sowing and conventional tillage. *Spanish Journal of Agricultural Research* **4**, 355–362 (2006).

32. Baumhardt, R. L., Wendt, C. W. & Keeling, J. W. Tillage and furrow diking effects on water balance and yields of sorghum and cotton. *Soil Science Society of America Journal* **57**, 1077–1083 (1993).

33. Baumhardt, R. . & Jones, O. . Residue management and tillage effects on soil-water storage and grain yield of dryland wheat and sorghum for a clay loam in Texas. *Soil and Tillage Research* **68**, 71–82 (2002).

34. Behnke, G. D., Zuber, S. M., Pittelkow, C. M., Nafziger, E. D. & Villamil, M. B. Long-term crop rotation and tillage effects on soil greenhouse gas emissions and crop production in Illinois, USA. *Agriculture, Ecosystems & Environment* **261**, 62–70 (2018).

35. Bermudez, M. & Mallarino, A. P. Corn response to starter fertilizer and tillage across and within fields having no-till management histories. *Agronomy Journal* **96**, 776–785 (2004).

36. Bernstein, E. R., Posner, J. L., Stoltenberg, D. E. & Hedtcke, J. L. Organically managed no-tillage rye-soybean systems: agronomic, economic, and environmental assessment. *Agronomy Journal* **103**, 1169–1179 (2011).

37. Beyaert, R. P., Schott, J. W. & White, P. H. Tillage effects on corn production in a coarse-textured soil in Southern Ontario. *Agronomy Journal* **94**, 767–774 (2002).

38. Bisen, P. K. & Singh, R. Effect of tillage and weed control practices on weed growth and yield of wheat (Triticum aestivum) in rice (Oryza sativa) - Wheat system. *Indian Journal of Agricultural Sciences* **78**, 347–350 (2008).

39. Blackshaw, R. E., Semach, G., Li, X., O'Donovan, J. T. & Harker, K. N. Tillage, fertiliser and glyphosate timing effects on foxtail barley (Hordeum jubatum) management in wheat. *Canadian Journal of Plant Science* **80**, 655–660 (2000).

40. Bocianowski, J., Szulc, P. & Nowosad, K. Soil tillage methods by years interaction for dry matter of plant yield of maize (Zea mays L.) using additive main effects and multiplicative interaction model. *Journal of Integrative Agriculture* **17**, 2836–2839 (2018).

41. Boeckx, P., Van Nieuland, K. & Van Cleemput, O. Short-term effect of tillage intensity on N2O and CO2 emissions. *Agronomy for Sustainable Development* **31**, 453–461 (2011).

42. Boehmel, C., Lewandowski, I. & Claupein, W. Comparing annual and perennial energy cropping systems with different management intensities. *Agricultural Systems* **96**, 224–236 (2008).

43. Bogunovic, I., Pereira, P., Kisic, I., Sajko, K. & Sraka, M. Tillage management impacts on soil compaction, erosion and crop yield in Stagnosols (Croatia). *CATENA* **160**, 376–384 (2018).

44. Bono, A., Alvarez, R., Buschiazzo, D. E. & Cantet, R. J. C. Tillage Effects on Soil Carbon Balance in a Semiarid Agroecosystem. *Soil Science Society of America Journal* **72**, 1140–1149 (2008).

45. Bordovsky, J. P., Lyle, W. M. & Keeling, J. W. Crop rotation and tillage rffects on soil water and cotton yield. *Agronomy Journal* **86**, 1–6 (1994).

46. Botta, G. F., Tolon-Becerra, A., Lastra-Bravo, X. & Tourn, M. Tillage and traffic effects (planters and tractors) on soil compaction and soybean (Glycine max L.) yields in Argentinean pampas. *Soil and Tillage Research* **110**, 167–174 (2010).

47. Brandt, S. A. Zero vs. conventional tillage and their effects on crop yield and soil moisture. *Canadian Journal of Plant Science* **72**, 679–688 (1992).

48. Brown, V., Barbosa, F. T., Bertol, I., Mafra, Á. L. & Muzeka, L. M. Effects on soil and crops after 20 years of conventional and zero tillage. *Revista Brasileira de Ciências Agrárias - Brazilian Journal of Agricultural Sciences* **13**, 1–7 (2018).

49. Büchi, L., Wendling, M., Amossé, C., Necpalova, M. & Charles, R. Importance of cover crops in alleviating negative effects of reduced soil tillage and promoting soil fertility in a winter wheat cropping system. *Agriculture, Ecosystems & Environment* **256**, 92–104 (2018).

50. Busscher, W. J., Khalilian, A. & Jones, M. A. Tillage Management for Cotton in Southeastern Coastal Soils during Dry Years. *Communications in Soil Science and Plant Analysis* **43**, 2564–2574 (2012).

51. Cahoon, J. E. *et al.* Corn Yield Response to Tillage with Furrow Irrigation. *Journal of Production Agriculture* **12**, 269–275 (1999).

52. Calzarano, F. *et al.* Durum wheat quality, yield and sanitary status under conservation agriculture. *Agriculture (Switzerland)* **8**, 1–13 (2018).

53. Camarotto, C. *et al.* Conservation agriculture and cover crop practices to regulate water, carbon and nitrogen cycles in the low-lying Venetian plain. *Catena* **167**, 236–249 (2018).

54. Campbell, C. A. *et al.* Converting from no-tillage to pre-seeding tillage: Influence on weeds, spring wheat grain yields and N, and soil quality. *Soil and Tillage Research* **46**, 175–185 (1998).

55. Campbell, D. J., Dickson, J. W., Ball, B. C. & Hunter, R. Controlled seedbed traffic after ploughing or direct drilling under winter barley in Scotland, 1980–1984. *Soil and Tillage Research* **8**, 3–28 (1986).

56. Cannell, R. Q., Christian, D. G. & Henderson, F. K. G. A study of mole drainage with simplified cultivation for autumn-sown crops on a clay soil. 4. A comparison of direct drilling and mouldboard ploughing on drained and undrained land on root and shoot growth, nutrient uptake and yield. *Soil and Tillage Research* **7**, 251–272 (1986).

57. Cantero-Martínez, C., Angás, P. & Lampurlanés, J. Long-term yield and water use efficiency under various tillage systems in Mediterranean rainfed conditions. *Annals of Applied Biology* **150**, 293–305 (2007).

58. Carefoot, J. M., Nyborg, M. & Lindwall, C. W. Tillage-induced soil changes and related grain yield in a semi-arid region. *Canadian Journal of Soil Science* **70**, 203–214 (1990).

59. Carignano, M., Staggenborg, S. A. & Shroyer, J. P. Management practices to minimize tan spot in a continuous wheat rotation. *Agronomy Journal* **100**, 145–153 (2008).

60. Carof, M., Tourdonnet, S., Saulas, P., Floch, D. & Roger-Estrade, J. Undersowing wheat with different living mulches in a no-till system. I. Yield analysis. *Agronomy for Sustainable Development* **27**, 347–356 (2007).

61. Cassel, D. K., Raczkowski, C. W. & Denton, H. P. Tillage Effects on Corn Production and Soil Physical Conditions. *Soil Science Society of America Journal* **59**, 1436–1443 (1995).

62. Cavigelli, M. A., Teasdale, J. R. & Conklin, A. E. Long-term agronomic performance of organic and conventional field crops in the Mid-Atlantic region. *Agronomy Journal* **100**, 785–794 (2008).

63. Celik, I. *et al.* Impacts of different tillage practices on some soil microbiological properties and crop yield under semi-arid Mediterranean conditions. *International Journal of Plant Production* **5**, 237–254 (2011).

64. Chatskikh, D. & Olesen, J. E. Soil tillage enhanced CO2 and N2O emissions from loamy sand soil under spring barley. *Soil and Tillage Research* **97**, 5–18 (2007).

65. Chauhan, B. S. & Opeña, J. Effect of tillage systems and herbicides on weed emergence, weed growth, and grain yield in dry-seeded rice systems. *Field Crops Research* **137**, 56–69 (2012).

66. Chen, S. Y., Stienstra, W. C., Lueschen, W. E. & Hoverstad, T. R. Response of Heterodera glycines and soybean cultivar to tillage and row spacing. *Plant Disease* **85**, 311–316 (2001).

67. Chen, S. Tillage and crop sequence effects on Heterodera glycines and soybean yields. *Agronomy Journal* **99**, 797–807 (2007).

68. Chikoye, D., Udensi, U. E. & Ogunyemi, S. Integrated management of cogongrass [imperata cylindrica (L.) rauesch.] in corn using tillage, glyphosate, row spacing, cultivar, and cover cropping. *Agronomy Journal* **97**, 1164–1171 (2005).

69. Christian, D. G. & Bacon, E. T. G. A long-term comparison of ploughing, tine cultivation and direct drilling on the growth and yield of winter cereals and oilseed rape on clayey and silty soils. *Soil and Tillage Research* **18**, 311–331 (1990).

70. Cline, G. R. & Silvernail, A. F. Effects of cover crops, nitrogen, and tillage on sweet corn. *HortTechnology* **12**, 118–125 (2002).

71. Cociu, A. I. Long-term tillage and crop sequence effects on Maize and Soybean grain yield under eastern Romanian danube plain climate conditions. *Romanian Agricultural Research* **2019**, (2019).

72. Cociu, A. I. Long-term tillage and crop sequence effects on Winter wheat and Triticale grain yield under eastern Romanian danube plain climate conditions. *Romanian Agricultural Re* **36**, (2019).

73. Costa, S. E. V. G. A. *et al.* Patterns in phosphorus and corn root distribution and yield in long-term tillage systems with fertilizer application. *Soil and Tillage Research* **109**, 41–49 (2010).

74. Coventry, D. R., Hirth, J. R. & Reeves, T. G. Interactions of tillage and lime in wheat-subterranean clover rotations on an acidic sandy clay loam in southeastern Australia. *Soil and Tillage Research* **25**, 53–65 (1992).

75. Cox, W. J., Zobel, R. W., van Es, H. M. & Otis, D. J. Growth development and yield of maize under three tillage systems in the northeastern U.S.A. *Soil and Tillage Research* **18**, 295–310 (1990).

76. Cullum, R. F. Influence of tillage on maize yield in soil with shallow fragipan. *Soil and Tillage Research* **119**, 1–6 (2012).

77. D. C. Erbach *et al.* Maize response to tillage-induced soil conditions. *Transactions of the ASAE* **29**, 690–695 (1986).

78. Daigh, A. L. M. *et al.* Yields and yield stability of no-till and chisel-plow fields in the Midwestern US Corn Belt. *Field Crops Research* **218**, 243–253 (2018).

79. Dam, R. F. *et al.* Soil bulk density and crop yield under eleven consecutive years of corn with different tillage and residue practices in a sandy loam soil in central Canada. *Soil and Tillage Research* **84**, 41–53 (2005).

80. Das, T. K. *et al.* Conservation agriculture effects on crop and water productivity, profitability and soil organic carbon accumulation under a maize-wheat cropping system in the North-western Indo-Gangetic Plains. *Field Crops Research* **215**, 222–231 (2018).

81. de Cárcer, P. S., Sinaj, S., Santonja, M., Fossati, D. & Jeangros, B. Long-term effects of crop succession, soil tillage and climate on wheat yield and soil properties. *Soil and Tillage Research* **190**, 209–219 (2019).

82. De Vita, P., Di Paolo, E., Fecondo, G., Di Fonzo, N. & Pisante, M. No-tillage and conventional tillage effects on durum wheat yield, grain quality and soil moisture content in southern Italy. *Soil and Tillage Research* **92**, 69–78 (2007).

83. Decker, J. E., Epplin, F. M., Morley, D. L. & Peeper, T. F. Economics of five wheat production systems with no-till and conventional tillage. *Agronomy Journal* **101**, 364–372 (2009).

84. Dekemati, I., Simon, B., Vinogradov, S. & Birkás, M. The effects of various tillage treatments on soil physical properties, earthworm abundance and crop yield in Hungary. *Soil and Tillage Research* **194**, 104334 (2019).

85. Díaz-Zorita, M. Effect of deep-tillage and nitrogen fertilization interactions on dryland corn (Zea mays L.) productivity. *Soil and Tillage Research* **54**, 11–19 (2000).

86. Díaz-Zorita, M., Grove, J. H., Murdock, L., Herbeck, J. & Perfect, E. Soil Structural Disturbance Effects on Crop Yields and Soil Properties in a No-Till Production System. *Agronomy Journal* **96**, 1651–1659 (2004).

87. Díaz-Zorita, M., Duarte, G. A. & Grove, J. H. A review of no-till systems and soil management for sustainable crop production in the subhumid and semiarid Pampas of Argentina. *Soil and Tillage Research* **65**, 1–18 (2002).

88. Dickey, E. C., Jasa, P. J. & Grisso, R. D. Long term tillage effects on grain yield and soil properties in a soybean/grain sorghum rotation. *Journal of Production Agriculture* **7**, 465–470 (1994).

89. Dixit, A. K. *et al.* Soil properties, crop productivity and energetics under different tillage practices in fodder sorghum + cowpea–wheat cropping system. *Archives of Agronomy and Soil Science* **65**, 492–506 (2019).

90. Domínguez, G. F., Diovisalvi, N. V., Studdert, G. A. & Monterubbianesi, M. G. Soil organic C and N fractions under continuous cropping with contrasting tillage systems on mollisols of the southeastern Pampas. *Soil and Tillage Research* **102**, 93–100 (2009).

91. Donald, P. A., Tyler, D. D. & Boykin, D. L. Short- and long-term tillage effects on Heterodera glycines reproduction in soybean monoculture in west Tennessee. *Soil and Tillage Research* **104**, 126–133 (2009).

92. Drinkwater, L. E., Janke, R. R. & Rossoni-Longnecker, L. Effects of tillage intensity on nitrogen dynamics and productivity in legume-based grain systems. *Plant and Soil* **227**, 99–113 (2000).

93. Drury, C. F. *et al.* Emissions of nitrous oxide and carbon dioxide: influence of tillage type and nitrogen placement depth. *Soil Science Society of America Journal* **70**, 570–581 (2006).

94. Drury, C. F. *et al.* Nitrogen source, application time, and tillage effects on soil nitrous oxide emissions and corn grain yields. *Soil Science Society of America Journal* **76**, 1268–1279 (2012).

95. Drury, C. F. *et al.* Impacts of Zone Tillage and Red Clover on Corn Performance and Soil Physical Quality. *Soil Science Society of America Journal* **67**, 867 (2003).

96. Duiker, S. W. & Curran, W. S. Rye cover crop management for corn production in the Northern Mid-Atlantic region. *Agronomy Journal* **97**, 1413–1418 (2005).

97. Edwards, J. H., Thurlow, D. L. & Eason, J. T. Influence of Tillage and Crop Rotation on Yields of Corn, Soybean, and Wheat. *Agronomy Journal* **80**, 76 (1988).

98. Eghball, B. & Power, J. F. Composted and noncomposted manure application to conventional and no-tillage systems: corn yield and nitrogen uptake. *Agronomy Journal* **91**, 819–825 (1999).

99. Ellington, A. Effects of deep ripping, direct drilling, gypsum and lime on soils, wheat growth and yield. *Soil and Tillage Research* **8**, 29–49 (1986).

100. Endale, D. M. *et al.* Impact of conservation tillage and nutrient management on soil water and yield of cotton fertilized with poultry litter or ammonium nitrate in the Georgia Piedmont. *Soil and Tillage Research* **66**, 55–68 (2002).

101. Endale, D. M. *et al.* No-till corn productivity in a southeastern United States ultisol amended with poultry litter. *Agronomy Journal* **100**, 1401–1408 (2008).

102. Ernani, P. R., Bayer, C. & Maestri, L. Corn yield as affected by liming and tillage system on an acid Brazilian oxisol. *Agronomy Journal* **94**, 305–309 (2002).

103. Fecák, P., Šariková, D. & Černý, I. Influence of tillage system and starting N fertilization on seed yield and quality of soybean Glycine max (L.) Merrill. *Plant, Soil and Environment* **56**, 105–110 (2010).

104. Feng, F. X., Huang, G. B., Chai, Q. & Yu, A. Z. Tillage and straw management impacts on soil properties, root growth, and grain yield of winter wheat in Northwestern China. *Crop Science* **50**, 1465–1473 (2010).

105. Fengyun, Z., Pute, W., Xining, Z., Enhe, Z. & Xuefeng, C. Effects of conservation tillage on soil water regimes and water use efficiency in farmland of Heihe River Basin in Northwest China. *African Journal of Agricultural Research* **6**, 5959–5966 (2011).

106. Filipovic, D., Husnjak, S., Kosutic, S. & Gospodaric, Z. Effects of tillage systems on compaction and crop yield of Albic Luvisol in Croatia. *Journal of Terramechanics* **43**, 177–189 (2006).

107. Fiorini, A., Maris, S. C., Abalos, D., Amaducci, S. & Tabaglio, V. Combining no-till with rye (Secale cereale L.) cover crop mitigates nitrous oxide emissions without decreasing yield. *Soil and Tillage Research* **196**, 104442 (2020).

108. Fischer, R. A., Santiveri, F. & Vidal, I. R. Crop rotation, tillage and crop residue management for wheat and maize in the sub-humid tropical highlands I. Wheat and legume performance. *Field Crops Research* **79**, 107–122 (2002).

109. Fischer, R. A., Santiveri, F. & Vidal, I. R. Crop rotation, tillage and crop residue management for wheat and maize in the sub-humid tropical highlands II. Maize and system performance. *Field Crops Research* **79**, 123–137 (2002).

110. Flowers, M. . & Lal, R. Axle load and tillage effects on soil physical properties and soybean grain yield on a mollic ochraqualf in northwest Ohio. *Soil and Tillage Research* **48**, 21–35 (1998).

111. Franchini, J. C. *et al.* Evolution of crop yields in different tillage and cropping systems over two decades in southern Brazil. *Field Crops Research* **137**, 178–185 (2012).

112. Franzluebbers, A. J. & Stuedemann, J. A. Crop and cattle responses to tillage systems for integrated crop–livestock production in the Southern Piedmont, USA. *Renewable Agriculture and Food Systems* **22**, 168–180 (2007).

113. Gajri, P. R., Arora, V. K. & Prihar, S. S. Tillage management for efficient water and nitrogen use in wheat following rice. *Soil and Tillage Research* **24**, 167–182 (1992).

114. Galvez, L., Douds, D. D., Drinkwater, L. E. & Wagoner, P. Effect of tillage and farming system upon VAM fungus populations and mycorrhizas and nutrient uptake of maize. *Plant and Soil* **228**, 299–308 (2001).

115. Galvez, L., Douds, D. D. & Wagoner, P. Tillage and farming system affect AM fungus populations, mycorrhizal formation, and nutrient uptake by winter wheat in a high-P soil. *American Journal of Alternative Agriculture* **16**, 152–160 (2001).

116. Gangwar, K. S., Singh, K. K. & Sharma, S. K. Effect of tillage on growth, yield and nutrient uptake in wheat after rice in the Indo-Gangetic Plains of India. *The Journal of Agricultural Science* **142**, 453–459 (2004).

117. Gangwar, K. S., Singh, K. K., Sharma, S. K. & Tomar, O. K. Alternative tillage and crop residue management in wheat after rice in sandy loam soils of Indo-Gangetic plains. *Soil and Tillage Research* **88**, 242–252 (2006).

118. Gao, J., Hao, X., Thelen, K. D. & Robertson, G. P. Agronomic management system and precipitation effects on soybean oil and fatty acid profiles. *Crop Science* **49**, 1049–1057 (2009).

119. Gathala, M. K. *et al.* Effect of tillage and crop establishment methods on physical properties of a medium-textured soil under a seven-year rice−wheat rotation. *Soil Science Society of America Journal* **75**, 1851–1862 (2011).

120. Ghuman, B. S. & Sur, H. S. Tillage and residue management effects on soil properties and yields of rainfed maize and wheat in a subhumid subtropical climate. *Soil and Tillage Research* **58**, 1–10 (2001).

121. Giannitsopoulos, M. L., Burgess, P. J. & Rickson, R. J. Effects of conservation tillage systems on soil physical changes and crop yields in a wheat–oilseed rape rotation. *Journal of Soil and Water Conservation* **74**, 247–258 (2019).

122. Gracia-Romero, A. *et al.* Phenotyping conservation agriculture management effects on ground and aerial remote sensing assessments of maize hybrids performance in Zimbabwe. *Remote Sensing* **10**, 349 (2018).

123. Grageda-Cabrera, O. A. *et al.* Fertilizer dynamics in different tillage and crop rotation systems in a Vertisol in Central Mexico. *Nutrient Cycling in Agroecosystems* **89**, 125–134 (2011).

124. Grandy, A. S., Robertson, G. P. & Thelen, K. D. Do productivity and environmental trade-offs justify periodically cultivating no-till cropping systems? *Agronomy Journal* **98**, 1377–1383 (2006).

125. Grant, C. A. & Bailey, L. D. The effect of KCl, KNO 3 , and CaCl 2 fertilization under conventional- and zero-till systems on common root rot, dry matter yield and grain yield of Heartland barley. *Canadian Journal of Plant Science* **74**, 1–6 (1994).

126. Griffith, D. R., Kladivko, E. J., Mannering, J. V, West, T. D. & Parsons, S. D. Long-term tillage and rotation effects on corn growth and yield on high and low organic matter, poorly drained soils. *Agronomy Journal* **80**, 599–605 (1988).

127. Grigoras, M. A., Popescu, A., Pamfil, D., Has, I. & Gidea, M. Influence of no-tillage agriculture system and fertilization on wheat yield and grain protein and gluten contents. *Journal of Food, Agriculture and Environment* **10**, 532–539 (2012).

128. Gruber, S., Pekrun, C., Möhring, J. & Claupein, W. Long-term yield and weed response to conservation and stubble tillage in SW Germany. *Soil and Tillage Research* **121**, 49–56 (2012).

129. Gul, B., Marwat, K. B., Saeed, M., Hussain, Z. & Ali, H. Impact of tillage, plant population and mulches on weed management and grain yield of maize. *Pakistan Journal of Botany* **46**, 1603–1606 (2011).

130. Guo, Y. *et al.* Reduced irrigation and nitrogen coupled with no-tillage and plastic mulching increase wheat yield in maize-wheat rotation in an arid region. *Field Crops Research* **243**, 107615 (2019).

131. Guy, S. O. & Cox, D. B. Reduced tillage increases residue groundcover in subsequent dry pea and winter wheat crops in the Palouse region of Idaho. *Soil and Tillage Research* **66**, 69–77 (2002).

132. Gwenzi, W., Gotosa, J., Chakanetsa, S. & Mutema, Z. Effects of tillage systems on soil organic carbon dynamics, structural stability and crop yields in irrigated wheat (Triticum aestivum L.)–cotton (Gossypium hirsutum L.) rotation in semi-arid Zimbabwe. *Nutrient Cycling in Agroecosystems* **83**, 211–221 (2009).

133. Halvorson, A. D., Black, A. L., Krupinsky, J. M., Merrill, S. D. & Tanaka, D. L. Sunflower response to tillage and nitrogen fertilization under intensive cropping in a wheat rotation. *Agronomy Journal* **91**, 637–642 (1999).

134. Halvorson, A. D., Black, A. L., Krupinsky, J. M. & Merrill, S. D. Dryland winter wheat response to tillage and nitrogen within an annual cropping system. *Agronomy Journal* **91**, 702–707 (1999).

135. Halvorson, A. D., Mosier, A. R., Reule, C. A. & Bausch, W. C. Nitrogen and tillage effects on irrigated continuous corn yields. *Agronomy Journal* **98**, 63–71 (2006).

136. Halwani, M., Reckling, M., Schuler, J., Bloch, R. & Bachinger, J. Soybean in no-till cover-crop systems. *Agronomy* **9**, 883 (2019).

137. Hamblin, A. P. The effect of tillage on soil surface properties and the water balance of a xeralfic alfisol. *Soil and Tillage Research* **4**, 543–559 (1984).

138. He, W. *et al.* Estimating the impacts of climate change on crop yields and N2O emissions for conventional and no-tillage in Southwestern Ontario, Canada. *Agricultural Systems* **159**, 187–198 (2018).

139. Hemmat, A. & Eskandari, I. Dryland winter wheat response to conservation tillage in a continuous cropping system in northwestern Iran. *Soil and Tillage Research* **86**, 99–109 (2006).

140. Hemmat, A. & Eskandari, I. Tillage system effects upon productivity of a dryland winter wheat–chickpea rotation in the northwest region of Iran. *Soil and Tillage Research* **78**, 69–81 (2004).

141. Hendrix, B. J., Young, B. G. & Chong, S. K. Weed Management in Strip Tillage Corn. *Agronomy Journal* **96**, 229–235 (2004).

142. Holanda, F. S. R. *et al.* Contribution of tillage systems on the organic matter of Gley soil and the productivity of corn and soybean. *Semina:Ciencias Agrarias* **32**, 983–994 (2011).

143. Hou, X. *et al.* Effects of rotational tillage practices on soil properties, winter wheat yields and water-use efficiency in semi-arid areas of north-west China. *Field Crops Research* **129**, 7–13 (2012).

144. Houria, C., Hannachi, A., Fellahi, Z. E. A. & Bouzerzour, H. Tillage and residue management effect on durum wheat [Triticum turgidum (L.) thell. ssp. turgidum conv. durum (Desf.) mackey] growth and yield under semi arid climate. *Advances in Environmental Biology* **5**, 3231–3241 (2011).

145. Houšť, M., Procházková, B. & Hledík, P. Effect of different tillage intensity on yields and yield-forming factors in winter wheat. *Acta Universitatis Agriculturae et Silviculturae Mendelianae Brunensis* **60**, 89–96 (2012).

146. Howard, D. D., Gwathmey, C. O., Roberts, R. K. & Lessman, G. M. Potassium fertilization of cotton on two high testing soils under two tillage systems. *Journal of Plant Nutrition* **20**, 1645–1656 (1997).

147. Hu, W. *et al.* Effects of tillage, compaction and nitrogen inputs on crop production and nitrogen losses following simulated forage crop grazing. *Agriculture, Ecosystems and Environment* **289**, (2020).

148. Huang, G. B. *et al.* Productivity and sustainability of a spring wheat-field pea rotation in a semi-arid environment under conventional and conservation tillage systems. *Field Crops Research* **107**, 43–55 (2008).

149. Huang, M., Jiang, P., Zhou, X. & Zou, Y. No-tillage increases nitrogen scavenging by fallow weeds in a double-season rice cropping system in China. *Weed Biology and Management* **18**, 105–109 (2018).

150. Huang, M. *et al.* No-tillage and direct seeding for super hybrid rice production in rice–oilseed rape cropping system. *European Journal of Agronomy* **34**, 278–286 (2011).

151. Huang, M. *et al.* Effect of tillage on soil and crop properties of wet-seeded flooded rice. *Field Crops Research* **129**, 28–38 (2012).

152. Huang, X., Wang, L., Yang, L. & Kravchenko, A. N. Management effects on relationships of crop yields with topography represented by wetness index and precipitation. *Agronomy Journal* **100**, 1463–1471 (2008).

153. Hunt, P. G., Bauer, P. J., Matheny, T. A. & Busscher, W. J. Crop yield and nitrogen accumulation response to tillage of a coastal plain soil. *Crop Science* **44**, 1673–1681 (2004).

154. Huynh, H. T., Hufnagel, J., Wurbs, A. & Bellingrath-Kimura, S. D. Influences of soil tillage, irrigation and crop rotation on maize biomass yield in a 9-year field study in Müncheberg, Germany. *Field Crops Research* **241**, 107565 (2019).

155. Iijima, M., Asai, T., Zegada-Lizarazu, W., Nakajima, Y. & Hamada, Y. Productivity and water source of intercropped wheat and rice in a direct-sown sequential cropping system: the effects of no-tillage and drought. *Plant Production Science* **8**, 368–374 (2005).

156. Iqbal, M., Anwar-ul-Hassan & Ibrahim, M. Effects of tillage systems and mulch on soil physical quality parameters and maize (Zea mays L.) yield in semi-arid Pakistan. *Biological Agriculture & Horticulture* **25**, 311–325 (2008).

157. Iragavarapu, T. K. & Randall, G. W. Yield and nitrogen uptake of monocropped maize from a long-term tillage experiment on a poorly drained soil. *Soil and Tillage Research* **34**, 145–156 (1995).

158. Irmak, S., Kukal, M. S., Mohammed, A. T. & Djaman, K. Disk-till vs. no-till maize evapotranspiration, microclimate, grain yield, production functions and water productivity. *Agricultural Water Management* **216**, 177–195 (2019).

159. Ishaque, W. *et al.* Short-term effects of tillage and residue management practices on dry matter yield and fate of 15N-urea in a continuous maize cropping system under subtropical conditions. *Soil and Tillage Research* **182**, 78–85 (2018).

160. Ismail, I., Blevins, R. L. & Frye, W. W. Long-term no-tillage effects on soil properties and continuous corn yields. *Soil Science Society of America Journal* **58**, 193–198 (1994).

161. Issaka, F. *et al.* Zero tillage improves soil properties, reduces nitrogen loss and increases productivity in a rice farmland in Ghana. *Agronomy* **9**, 1–13 (2019).

162. Izaurralde, R. C., Choudhary, M., Juma, N. G., McGill, W. B. & Haderlein, L. Crop and nitrogen yield in legume-based rotations practiced with zero tillage and low-input methods. *Agronomy Journal* **87**, 958–964 (1995).

163. Izumi, Y., Uchida, K. & Iijima, M. Crop production in successive wheat-soybean rotation with no-tillage practice in relation to the root system development. *Plant Production Science* **7**, 329–336 (2004).

164. Jat, M. L. *et al.* Evaluation of precision land leveling and double zero-till systems in the rice-wheat rotation: Water use, productivity, profitability and soil physical properties. *Soil and Tillage Research* **105**, 112–121 (2009).

165. Jat, R. K. *et al.* Ten years of conservation agriculture in a rice–maize rotation of Eastern Gangetic Plains of India: Yield trends, water productivity and economic profitability. *Field Crops Research* **232**, 1–10 (2019).

166. Jones, M. J. Comparison of conservation tillage systems in barley-based cropping systems in Northern Syria. *Experimental Agriculture* **36**, 15–26 (2000).

167. Jones, O. R. & Popham, T. W. Cropping and tillage systems for dryland grain production in the Southern High Plains. *Agronomy Journal* **89**, 222–232 (1997).

168. Jug, D. *et al.* Effect of conservation tillage on crop productivity and nitrogen use efficiency. *Soil and Tillage Research* **194**, 104327 (2019).

169. Kabanza, A. K. & Rwehumbiza, F. B. R. Assessment of the contribution of tied ridges and farmyard manure application to sorghum production in semi-arid areas of Tanzania. *Advances in Integrated Soil Fertility Management in sub-Saharan Africa: Challenges and Opportunities* 723–730 (2007) doi:10.1007/978-1-4020-5760-1_67.

170. Kabir, Z., O'Halloran, I. P., Fyles, J. W. & Hamel, C. Seasonal changes of arbuscular mycorrhizal fungi as affected by tillage practices and fertilization: Hyphal density and mycorrhizal root colonization. *Plant and Soil* **192**, 285–293 (1997).

171. Kafesu, N. *et al.* Comparative fertilization effects on maize productivity under conservation and conventional tillage on sandy soils in a smallholder cropping system in Zimbabwe. *Field Crops Research* **218**, 106–114 (2018).

172. Kandel, T. P., Gowda, P. H., Northup, B. K. & Rocateli, A. C. Impacts of tillage systems, nitrogen fertilizer rates and a legume green manure on light interception and yield of winter wheat. *Cogent Food & Agriculture* **5**, 1–13 (2019).

173. Karlen, D. L., Hunt, P. G. & Matheny, T. A. Fertilizer 15 nitrogen recovery by corn, wheat, and cotton grown with and without pre-plant tillage on norfolk loamy sand. *Crop Science* **36**, 975–981 (1996).

174. Karlen, D. L., Berry, E. C., Colvin, T. S. & Kanwar, R. S. Twelve-year tillage and crop rotation effects on yields and soil chemical properties in northeast Iowa. *Communications in Soil Science and Plant Analysis* **22**, 1985–2003 (1991).

175. Karlen, D. L., Kovar, J. L., Cambardella, C. A. & Colvin, T. S. Thirty-year tillage effects on crop yield and soil fertility indicators. *Soil and Tillage Research* **130**, 24–41 (2013).

176. Karunatilake, U., van Es, H. M. & Schindelbeck, R. R. Soil and maize response to plow and no-tillage after alfalfa-to-maize conversion on a clay loam soil in New York. *Soil and Tillage Research* **55**, 31–42 (2000).

177. Kennedy, C. W. & Hutchinson, R. L. Cotton growth and development under different tillage systems. *Crop Science* **41**, 1162–1168 (2001).

178. Kihara, J., Bationo, A., Mugendi, D. N., Martius, C. & Vlek, P. L. G. Conservation tillage, local organic resources and nitrogen fertilizer combinations affect maize productivity, soil structure and nutrient balances in semi-arid Kenya. *Nutrient Cycling in Agroecosystems* **90**, 213–225 (2011).

179. Kisić, I., Bašić, F., Birkas, M., Jurišić, A. & Bićanić, V. Crop yield and plant density under different tillage systems. *Agriculturae Conspectus Scientificus* **75**, 1–7 (2010).

180. Kitonyo, O. M., Sadras, V. O., Zhou, Y. & Denton, M. D. Nitrogen fertilization modifies maize yield response to tillage and stubble in a sub-humid tropical environment. *Field Crops Research* **223**, 113–124 (2018).

181. Kladivko, E. J., Griffith, D. R. & Mannering, J. V. Conservation tillage effects on soil properties and yield of corn and soya beans in Indiana. *Soil and Tillage Research* **8**, 277–287 (1986).

182. Knight, C. W. & Lewis, C. E. Conservation tillage in the Subarctic. *Soil and Tillage Research* **7**, 341–353 (1986).

183. Kobayashi, H., Miura, S. & Oyanagi, A. Effects of winter barley as a cover crop on the weed vegetation in a no-tillage soybean. *Weed Biology and Management* vol. 4 195–205 (2004).

184. Korucu, T. & Merdun, H. Effects of Tillage Systems on Wheat Yield and Residue in Turkey. *Journal of Animal and Veterinary Advances* **8**, 1973–1978 (2009).

185. Kumudini, S., Grabau, L., Van Sanford, D. & Omielan, J. Analysis of yield-formation processes under no-till and conventional tillage for soft red winter wheat in the south-central region. *Agronomy Journal* **100**, 1026–1032 (2008).

186. Kurle, J. E., Grau, C. R., Oplinger, E. S. & Mengistu, A. Tillage, crop sequence, and cultivar effects on sclerotinia stem rot incidence and yield in soybean. *Agronomy Journal* **93**, 973–982 (2001).

187. Kushwaha, C. P. & Singh, K. P. Crop productivity and soil fertility in a tropical dryland agro-ecosystem: Impact of residue and tillage management. *Experimental Agriculture* **41**, 39–50 (2005).

188. Kutcher, H. R., Johnston, A. M., Bailey, K. L. & Malhi, S. S. Managing crop losses from plant diseases with foliar fungicides, rotation and tillage on a Black Chernozem in Saskatchewan, Canada. *Field Crops Research* **124**, 205–212 (2011).

189. Lafond, G. P., May, W. E., Stevenson, F. C. & Derksen, D. A. Effects of tillage systems and rotations on crop production for a thin Black Chernozem in the Canadian Prairies. *Soil and Tillage Research* **89**, 232–245 (2006).

190. Lal, R. Axle load and tillage effects on crop yields on a Mollic Ochraqualf in Northwest Ohio. *Soil and Tillage Research* **37**, 143–160 (1996).

191. Lal, R. Long-term tillage and maize monoculture effects on a tropical Alfisol in western Nigeria. I. Crop yield and soil physical properties. *Soil and Tillage Research* **42**, 145–160 (1997).

192. Lal, R. Soil degradative effects of slope length and tillage methods on alfisols in western Nigeria. I. Runoff, erosion and crop response. *Land Degradation & Development* **8**, 201–219 (1997).

193. Lal, R. & Ahmadi, M. Axle load and tillage effects on crop yield for two soils in central Ohio. *Soil and Tillage Research* **54**, 111–119 (2000).

194. Lal, R., Logan, T. J. & Fausey, N. R. Long-term tillage and wheel traffic effects on a poorly drained mollic ochraqualf in northwest Ohio. 1. Soil physical properties, root distribution and grain yield of corn and soybean. *Soil and Tillage Research* **14**, 341–358 (1989).

195. Lampurlanés, J., Angás, P. & Cantero-Martínez, C. Root growth, soil water content and yield of barley under different tillage systems on two soils in semiarid conditions. *Field Crops Research* **69**, 27–40 (2001).

196. Landschoot, S. *et al.* Does shifting from conventional to zero tillage in combination with a cover crop offers opportunities for silage maize cultivation in Flanders? *Journal of Plant Nutrition and Soil Science* **182**, 980–989 (2019).

197. Latifmanesh, H. *et al.* Integrative impacts of rotational tillage on wheat yield and dry matter accumulation under corn-wheat cropping system. *Soil and Tillage Research* **184**, 100–108 (2018).

198. Latta, J. & O'Leary, G. J. Long-term comparison of rotation and fallow tillage systems of wheat in Australia. *Field Crops Research* **83**, 173–190 (2003).

199. Lawrance, K. F., Prinsloo, M. A. & Berry, W. A. J. Long and short term effects of tillage systems on grain yield, the incidence of diseases and production costs for maize. *South African Journal of Plant and Soil* **16**, 85–91 (1999).

200. Lenssen, A. W., Sainju, U. M., Allen, B. L., Jabro, J. D. & Stevens, W. B. Dryland corn production and water use affected by tillage and crop management intensity. *Agronomy Journal* **110**, 2439–2446 (2018).

201. Li, Z. *et al.* In search of long-term sustainable tillage and straw mulching practices for a maize-winter wheat-soybean rotation system in the Loess Plateau of China. *Field Crops Research* **217**, 199–210 (2018).

202. Licht, M. A. & Al-Kaisi, M. Corn response, nitrogen uptake, and water use in strip-tillage compared with no-tillage and chisel plow. *Agronomy Journal* **97**, 705–710 (2005).

203. Linden, D. R., Clapp, C. E. & Dowdy, R. H. Long-term corn grain and stover yields as a function of tillage and residue removal in east central Minnesota. *Soil and Tillage Research* **56**, 167–174 (2000).

204. Lithourgidis, A. S., Tsatsarelis, C. A. & Dhima, K. V. Tillage effects on corn emergence, silage yield, and labor and fuel inputs in double cropping with wheat. *Crop Science* **45**, 2523–2528 (2005).

205. Liu, J., Fan, Y., Ma, Y. & Li, Q. Response of photosynthetic active radiation interception, dry matter accumulation, and grain yield to tillage in two winter wheat genotypes. *Archives of Agronomy and Soil Science* 1–12 (2019) doi:10.1080/03650340.2019.1657232.

206. Liu, T., Huang, J., Chai, K., Cao, C. & Li, C. Effects of N fertilizer sources and tillage practices on NH3 volatilization, grain yield, and n use efficiency of rice fields in central China. *Frontiers in Plant Science* **9**, 1–10 (2018).

207. Liu, Z. *et al.* Soil organic carbon increment sources and crop yields under long-term conservation tillage practices in wheat-maize systems. *Land Degradation & Development* (2020) doi:10.1002/ldr.3531.

208. López, M. V. & Arrúe, J. L. Growth, yield and water use efficiency of winter barley in response to conservation tillage in a semi-arid region of Spain. *Soil and Tillage Research* **44**, 35–54 (1997).

209. López-Bellido, L., Fuentes, M., Castillo, J. E., López-Garrido, F. J. & Fernández, E. J. Long-term tillage, crop rotation, and nitrogen fertilizer effects on wheat yield under rainfed Mediterranean conditions. *Agronomy Journal* **88**, 783–791 (1996).

210. López-Bellido, L., López-Bellido, R. J., Castillo, J. E. & López-Bellido, F. J. Chickpea response to tillage and soil residual nitrogen in a continuous rotation with wheat. *Field Crops Research* **88**, 191–200 (2004).

211. López-Bellido, L. *et al.* Wheat response to nitrogen splitting applied to a Vertisols in different tillage systems and cropping rotations under typical Mediterranean climatic conditions. *European Journal of Agronomy* **43**, 24–32 (2012).

212. López-Vázquez, A., Cadena-Zapata, M., Campos-Magaña, S., Zermeño-Gonzalez, A. & Mendez-Dorado, M. Comparison of energy used and effects on bulk density and yield by tillage systems in a semiarid condition of Mexico. *Agronomy* **9**, 189 (2019).

213. Lötjönen, T. & Isolahti, M. Direct drilling of cereals after ley and slurry spreading. *Acta Agriculturae Scandinavica, Section B - Plant Soil Science* **60**, 307–319 (2010).

214. Lowery, B. Groundwater quality and crop-yield responses to tillage management on a Sparta sand. *Soil and Tillage Research* **48**, 225–237 (1998).

215. Lv, S. H. *et al.* An opportunity for regenerative rice production: Combining plastic film cover and plant biomass mulch with no-till soil management to build soil carbon, curb nitrogen pollution, and maintain high-stable yield. *Agronomy* **9**, 1–22 (2019).

216. Ma, Y. *et al.* Mitigation of nitrous oxide emissions from paddy soil under conventional and no-till practices using nitrification inhibitors during the winter wheat-growing season. *Biology and Fertility of Soils* **49**, 627–635 (2013).

217. Maali, S. H. & Agenbag, G. A. Effect of soil tillage, crop rotation and nitrogen application rates on grain yield of spring wheat (Triticum aestivum L.) in the Swartland wheat producing area of the Republic of South Africa. *South African Journal of Plant and Soil* **20**, 111–118 (2003).

218. Machado, S., Petrie, S., Rhinhart, K. & Qu, A. Long-term continuous cropping in the Pacific Northwest: Tillage and fertilizer effects on winter wheat, spring wheat, and spring barley production. *Soil and Tillage Research* **94**, 473–481 (2007).

219. Mahata, K. R., Sen, H. S., Pradhan, S. K. & Mandal, L. N. No-tillage and dry ploughing compared with puddling for wet-season rice on an alluvial sandy clay–loam in eastern India. *The Journal of Agricultural Science* **114**, 79–86 (1990).

220. Małecka, I., Blecharczyk, A., Sawinska, Z. & Dobrzeniecki, T. The effect of various long-term tillage systems on soil properties and spring barley yield. *Turkish Journal of Agriculture and Forestry* **36**, 217–226 (2012).

221. Malhi, S. S., Lemke, R., Wang, Z. H. & Chhabra, B. S. Tillage, nitrogen and crop residue effects on crop yield, nutrient uptake, soil quality, and greenhouse gas emissions. *Soil and Tillage Research* **90**, 171–183 (2006).

222. Malhi, S. S., McAndrew, D. W. & Carter, M. R. Effect of tillage and N fertilization of a Solonetzic soil on barley production and some soil properties. *Soil and Tillage Research* **22**, 95–107 (1992).

223. Malhi, S. S. & Lemke, R. Tillage, crop residue and N fertilizer effects on crop yield, nutrient uptake, soil quality and nitrous oxide gas emissions in a second 4-yr rotation cycle. *Soil and Tillage Research* **96**, 269–283 (2007).

224. Martínez, E., Fuentes, J.-P., Pino, V., Silva, P. & Acevedo, E. Chemical and biological properties as affected by no-tillage and conventional tillage systems in an irrigated Haploxeroll of Central Chile. *Soil and Tillage Research* **126**, 238–245 (2013).

225. Martin-Rueda, I. *et al.* Tillage and crop rotation effects on barley yield and soil nutrients on a Calciortidic Haploxeralf. *Soil and Tillage Research* **92**, 1–9 (2007).

226. Maurya, P. R. Effect of tillage and residue management of maize and wheat yield and on physical properties of an irrigated sandy loam soil in Northern Nigeria. *Soil and Tillage Research* **8**, 161–170 (1986).

227. McAndrew, D. W., Fuller, L. G. & Wetter, L. G. Grain and straw yields of barley under four tillage systems in northeastern Alberta. *Canadian Journal of Plant Science* **74**, 713–722 (1994).

228. McConkey, B. G., Campbell, C. A., Zentner, R. P., Dyck, F. B. & Selles, F. Long-term tillage effects on spring wheat production on three soil textures in the brown soil zone. *Canadian Journal of Plant Science* **76**, 747–756 (1996).

229. McConkey, B. G., Ulrich, D. J. & Dyck, F. B. Snow management and deep tillage for increasing crop yields on a rolling landscape. *Canadian Journal of Soil Science* **77**, 479–486 (1997).

230. Melero, S. *et al.* Long-term effect of tillage, rotation and nitrogen fertiliser on soil quality in a Mediterranean Vertisol. *Soil and Tillage Research* **114**, 97–107 (2011).

231. Messiga, A. J. *et al.* Long term impact of tillage practices and biennial P and N fertilization on maize and soybean yields and soil P status. *Field Crops Research* **133**, 10–22 (2012).

232. Mishra, J. S. & Singh, V. P. Tillage and weed control effects on productivity of a dry seeded rice-wheat system on a Vertisol in Central India. *Soil and Tillage Research* **123**, 11–20 (2012).

233. Mitra, B. *et al.* Nutrient management in wheat (Triticum aestivum) production system under conventional and zero tillage in eastern sub-Himalayan plains of India. *Indian Journal of Agricultural Sciences* **89**, 775–784 (2019).

234. Monneveux, P., Quillérou, E., Sanchez, C. & Lopez-Cesati, J. Effect of zero tillage and residues conservation on continuous maize cropping in a subtropical environment (Mexico). *Plant and Soil* **279**, 95–105 (2006).

235. Morell, F. J., Lampurlanés, J., Álvaro-Fuentes, J. & Cantero-Martínez, C. Yield and water use efficiency of barley in a semiarid Mediterranean agroecosystem: Long-term effects of tillage and N fertilization. *Soil and Tillage Research* **117**, 76–84 (2011).

236. Moret, D., Arrúe, J. L., López, M. V & Gracia, R. Winter barley performance under different cropping and tillage systems in semiarid Aragon (NE Spain). *European Journal of Agronomy* **26**, 54–63 (2007).

237. Morrison, M. J. *et al.* Tillage and crop rotation effects on the yield of corn, soybean and wheat in eastern Canada. *Canadian Journal of Plant Science* **98**, 183–191 (2017).

238. Mosier, A. R., Halvorson, A. D., Reule, C. A. & Liu, X. J. Net global warming potential and greenhouse gas intensity in irrigated cropping systems in Northeastern Colorado. *Journal of Environmental Quality* **35**, 1584–1598 (2006).

239. Mrabet, R. Differential response of wheat to tillage management systems in a semiarid area of Morocco. *Field Crops Research* **66**, 165–174 (2000).

240. Mtyobile, M., Muzangwa, L. & Mnkeni, P. N. S. Tillage and crop rotation effects on selected soil chemical properties and wheat yield in a sandy loam oakleaf soil in the Eastern Cape, South Africa. *International Journal of Agriculture and Biology* **21**, 367–374 (2019).

241. Mulugeta, D. & Stoltenberg, D. E. Weed and seedbank management with integrated methods as influenced by tillage. *Weed Science* **45**, 706–715 (1997).

242. Mutsamba, E. F., Nyagumbo, I. & Mupangwa, W. Forage and maize yields in mixed crop-livestock farming systems. *NJAS - Wageningen Journal of Life Sciences* 100317 (2019) doi:10.1016/j.njas.2019.100317.

243. Mutsamba, E. F., Nyagumbo, I. & Mupangwa, W. Forage and maize yields in mixed crop-livestock farming systems: Enhancing forage and maize yields in mixed crop-livestock systems under conservation agriculture in sub-humid Zimbabwe. *NJAS - Wageningen Journal of Life Sciences* 100317 (2019) doi:10.1016/j.njas.2019.100317.

244. Nandan, R. *et al.* Crop establishment with conservation tillage and crop residue retention in rice-based cropping systems of Eastern India: yield advantage and economic benefit. *Paddy and Water Environment* **16**, 477–492 (2018).

245. Narayan, D., Tiwari, A. K., Lal, B. & Katiyar, V. S. Effect of tillage practices and cover management on soil and water conservation and yield of sorghum. *Annals of Arid Zone* **48**, 133–138 (2009).

246. Nazirah, L., Purba, E., Hanum, C. & Rauf, A. Effect of soil tillage and mycorrhiza application on growth and yields of upland rice in drought condition. *Asian Journal of Agriculture and Biology* **6**, 251–258 (2018).

247. Ndoli, A. *et al.* Conservation agriculture with trees amplifies negative effects of reduced tillage on maize performance in East Africa. *Field Crops Research* **221**, 238–244 (2018).

248. Newton, A. C. *et al.* Soil tillage effects on the efficacy of cultivars and their mixtures in winter barley. *Field Crops Research* **128**, 91–100 (2012).

249. Ngwira, A. R., Aune, J. B. & Mkwinda, S. On-farm evaluation of yield and economic benefit of short term maize legume intercropping systems under conservation agriculture in Malawi. *Field Crops Research* **132**, 149–157 (2012).

250. Ngwira, A. R., Kabambe, V., Simwaka, P., Makoko, K. & Kamoyo, K. Productivity and profitability of maize-legume cropping systems under conservation agriculture among smallholder farmers in Malawi. *Acta Agriculturae Scandinavica Section B: Soil and Plant Science* **0**, 1–11 (2020).

251. Nielsen, D. C. *et al.* Cropping system influence on planting water content and yield of winter wheat. *Agronomy Journal* **94**, 962–967 (2002).

252. Noel, G. R. & Wax, L. M. Population dynamics of Heterodera glycines in conventional tillage and no-tillage soybean/corn cropping systems. *Journal of Nematology* **35**, 104–109 (2003).

253. Norwood, C. Profile water distribution and grain yield as affected by cropping system and tillage. *Agronomy Journal* **86**, 558–563 (1994).

254. Norwood, C. A. Water use and yield of dryland row crops as affected by tillage. *Agronomy Journal* **91**, 108–115 (1999).

255. Norwood, C. A. & Currie, R. S. Tillage, planting date, and plant population effects on dryland corn. *Journal of Production Agriculture* **9**, 119–122 (1996).

256. Nouri, A. *et al.* Soil physical properties and soybean yield as influenced by long-term tillage systems and cover cropping in the Midsouth USA. *Sustainability (Switzerland)* **10**, (2018).

257. Nyamadzawo, G., Nyamugafata, P., Wuta, M. & Nyamangara, J. Maize yields under coppicing and non coppicing fallows in a fallow–maize rotation system in central Zimbabwe. *Agroforestry Systems* **84**, 273–286 (2012).

258. Nyborg, M., Solberg, E. D., Izaurralde, R. C., Malhi, S. S. & Molina-Ayala, M. Influence of long-term tillage, straw and N fertilizer on barley yield, plant-N uptake and soil-N balance. *Soil and Tillage Research* **36**, 165–174 (1995).

259. Ogunremi, L. T., Lal, R. & Babalola, O. Effects of tillage methods and water regimes on soil properties and yield of lowland rice from a sandy loam soil in Southwest Nigeria. *Soil and Tillage Research* **6**, 223–234 (1986).

260. Ogunremi, L. T., Lal, R. & Babalola, O. Effects of tillage and seeding methods on soil physical properties and yield of upland rice for an ultisol in southeast Nigeria. *Soil and Tillage Research* **6**, 305–324 (1986).

261. Ojeniyi, S. O. Nutrient availability and maize yield under reduced tillage practices. *Soil and Tillage Research* **26**, 89–92 (1993).

262. Olson, K. R., Ebelhar, S. A. & Lang, J. M. Effects of 24 years of conservation tillage systems on soil organic carbon and soil productivity. *Applied and Environmental Soil Science* **2013**, (2013).

263. Opoku, G., Vyn, T. J. & Swanton, C. J. Modified no-till systems for corn following wheat on clay softs. *Agronomy Journal* **89**, 549–556 (1997).

264. Osuji, G. E. Water storage, water use and maize yield for tillage systems on a tropical alfisol in Nigeria. *Soil and Tillage Research* **4**, 339–348 (1984).

265. Ouédraogo, E., Mando, A., Brussaard, L. & Stroosnijder, L. Tillage and fertility management effects on soil organic matter and sorghum yield in semi-arid West Africa. *Soil and Tillage Research* **94**, 64–74 (2007).

266. Pabin, J., Lipiec, J., Włodek, S. & Biskupski, A. Maize response to different straw management and tillage systems under cereal crop rotation. *International Agrophysics* **20**, 141–146 (2006).

267. Pagnani, G. *et al.* Effect of soil tillage and crop sequence on grain yield and quality of durum wheat in Mediterranean areas. *Agronomy* **9**, 488 (2019).

268. Pala, M., Harris, H. C., Ryan, J., Makboul, R. & Dozom, S. Tillage systems and stubble management in a Mediterranean-type environment in relation to crop yield and soil moisture. *Experimental Agriculture* **36**, 223–242 (2000).

269. Pareja-Sánchez, E., Plaza-Bonilla, D., Álvaro-Fuentes, J. & Cantero-Martínez, C. Is it feasible to reduce tillage and N use while improving maize yield in irrigated Mediterranean agroecosystems? *European Journal of Agronomy* **109**, 125919 (2019).

270. Parihar, C. M. *et al.* Effects of precision conservation agriculture in a maize-wheat-mungbean rotation on crop yield, water-use and radiation conversion under a semiarid agro-ecosystem. *Agricultural Water Management* **192**, 306–319 (2017).

271. Parihar, C. M. *et al.* Conservation agriculture in irrigated intensive maize-based systems of north-western India: Effects on crop yields, water productivity and economic profitability. *Field Crops Research* **193**, 104–116 (2016).

272. Parihar, C. M. *et al.* Soil water dynamics, water productivity and radiation use efficiency of maize under multi-year conservation agriculture during contrasting rainfall events. *Field Crops Research* **241**, 107570 (2019).

273. Parihar, M. D. *et al.* Effect of different tillage and residue management practices on crop and water productivity and economics in maize (zea mays) based rotations. *Indian Journal of Agricultural Sciences* **89**, 360–366 (2019).

274. Parkin, T. B. & Kaspar, T. C. Nitrous oxide emissions from corn-soybean systems in the Midwest. *Journal of Environmental Quality* **35**, 1496–1506 (2006).

275. Peachey, B. E., William, R. D. & Mallory-smith, C. Effect of spring tillage sequence on summer annual weeds in vegetable row crop rotations. *Weed Technology* **20**, 204–214 (2018).

276. Pearce, A. D., Dillon, C. R., Keisling, T. C. & Wilson, C. E. Economic and agronomic effects of four tillage practices on rice produced on saline soils. *Journal of Production Agriculture* **12**, 305–312 (1999).

277. Pelster, D. E. *et al.* Nitrogen fertilization but not soil tillage affects nitrous oxide emissions from a clay loam soil under a maize–soybean rotation. *Soil and Tillage Research* **115–116**, 16–26 (2011).

278. Peng, Z. *et al.* Conservation tillage increases water use efficiency of spring wheat by optimizing water transfer in a semi-arid environment. *Agronomy* **9**, 583 (2019).

279. Perego, A. *et al.* Agro-environmental aspects of conservation agriculture compared to conventional systems: A 3-year experience on 20 farms in the Po valley (Northern Italy). *Agricultural Systems* **168**, 73–87 (2019).

280. Pettigrew, W. T. & Jones, M. A. Cotton growth under no-till production in the lower Mississippi river valley alluvial flood plain. *Agronomy Journal* **93**, 1398–1404 (2001).

281. Plaza-Bonilla, D. *et al.* No-tillage reduces long-term yield-scaled soil nitrous oxide emissions in rainfed Mediterranean agroecosystems: A field and modelling approach. *Agriculture, Ecosystems & Environment* **262**, 36–47 (2018).

282. Potter, K. N., Morrison, J. E. & Torbert, H. A. Tillage intensity effects on corn and grain sorghum growth and productivity on a vertisol. *Journal of Production Agriculture* **9**, 385–390 (1996).

283. Pradhan, P. R. *et al.* Tillage and crop residue management practices on crop productivity, phosphorus uptake and forms in wheat (Triticum aestivum)-based cropping systems. *Indian Journal of Agricultural Sciences* **81**, 1168–1173 (2011).

284. Qin, J. *et al.* The effect of mulching, tillage and rotation on yield in non-flooded compared with flooded rice production. *Journal of Agronomy and Crop Science* **196**, 397–406 (2010).

285. Quincke, J. A. *et al.* One-time tillage of no-till systems: Soil physical properties, phosphorus runoff, and crop yield. *Agronomy Journal* **99**, 1104–1110 (2007).

286. R. S. Kanwar, J. L. Baker & D. G. Baker. Tillage and split N-fertilization effects on subsurface drainage water quality and crop yields. *Transactions of the ASAE* **31**, 453–461 (1988).

287. Radford, B. J. & Thornton, C. M. Effects of 27 years of reduced tillage practices on soil properties and crop performance in the semi-arid subtropics of Australia. *International Journal of Energy Environment and Economics* **19**, 565 (2011).

288. Ram, H., Kler, D. S., Singh, Y. & Kumar, K. Productivity of maize (Zea mays) - wheat (Triticum aestivum) system under different tillage and crop establishment practices. *Indian Journal of Agronomy* **55**, 185–190 (2010).

289. Ram, H., Kumar, K., Kler, D. S. & Singh, Y. Effect of permanent bed planting and tillage options on microenvironment, crop productivity, water use efficiency, and soil properties under soybean (Glycine max L.) - wheat (Triticum aestivum L.) cropping system. *Ecology, Environment and Conservation* **16**, 593–599 (2010).

290. Ramos, M. C., Pareja-Sánchez, E., Plaza-Bonilla, D., Cantero-Martínez, C. & Lampurlanés, J. Soil sealing and soil water content under no-tillage and conventional tillage in irrigated corn: Effects on grain yield. *Hydrological Processes* **33**, 2095–2109 (2019).

291. Rao, S. C. & Dao, T. H. Nitrogen placement and tillage effects on dry matter and nitrogen accumulation and redistribution in winter wheat. *Agronomy Journal* **88**, 365–371 (1996).

292. Rashid, M. H., Timsina, J., Islam, N. & Islam, S. Tillage and residue-management effects on productivity, profitability and soil properties in a rice-maize-mungbean system in the Eastern Gangetic Plains. *Journal of Crop Improvement* **33**, 683–710 (2019).

293. Rasmussen, P. E. & Douglas, C. L. The influence of tillage and cropping-intensity on cereal response to nitrogen, sulfur, and phosphorus. *Fertilizer Research* **31**, 15–19 (1992).

294. Rasse, D. P. & Smucker, A. J. M. Tillage effects on soil nitrogen and plant biomass in a corn-alfalfa rotation. *Journal of Environmental Quality* **28**, 873–880 (1999).

295. Reddy, C. K., Nyakatawa, E. Z. & Reeves, D. W. Tillage and poultry litter application effects on cotton growth and yield. *Agronomy Journal* **96**, 1641–1650 (2004).

296. Reddy, S. S. *et al.* Long-term effects of poultry litter and conservation tillage on crop yields and soil phosphorus in cotton–cotton–corn rotation. *Field Crops Research* **114**, 311–319 (2009).

297. Reddy, S. S., Nyakatawa, E. Z. & Reddy, C. K. Nitrogen uptake pattern by cotton in a long-term no-tillage system with poultry litter application. *International Journal of Agriculture and Biology* **14**, 29–37 (2012).

298. Rembon, F. S. & MacKenzie, A. F. Soybean nitrogen contribution to corn and residual nitrate under conventional tillage and no-till. *Canadian Journal of Soil Science* **77**, 543–551 (1997).

299. Ren, Y., Gao, C., Han, H. & Li, Q. Response of water use efficiency and carbon emission to no-tillage and winter wheat genotypes in the North China Plain. *Science of The Total Environment* **635**, 1102–1109 (2018).

300. Renner, K. A., Schabenberger, O. & Kells, J. J. Effect of tillage and application method on corn (Zea mays) response to imidazolinone residues in soil. *Weed Technology* **12**, 281–285 (1998).

301. Ribera, L. A., Hons, F. M. & Richardson, J. W. An economic comparison between conventional and no-tillage farming systems in Burleson county, Texas. *Agronomy Journal* **96**, 415–424 (2004).

302. Rochette, P., Angers, D. A., Chantigny, M. H. & Bertrand, N. Nitrous oxide emissions respond differently to no-till in a loam and a heavy clay soil. *Soil Science Society of America Journal* **72**, 1363–1369 (2008).

303. Roper, M. M., Ward, P. R., Keulen, A. F. & Hill, J. R. Under no-tillage and stubble retention, soil water content and crop growth are poorly related to soil water repellency. *Soil and Tillage Research* **126**, 143–150 (2013).

304. Rozas, H. S., Echeverría, H. E., Studdert, G. A. & Domínguez, G. Evaluation of the presidedress soil nitrogen test for no-tillage maize fertilized at planting. *Agronomy Journal* **92**, 1176–1183 (2000).

305. Saha, S. *et al.* Effect of tillage and residue management on soil physical properties and crop productivity in maize (Zea mays)-Indian mustard (Brassica juncea) system. *Indian Journal of Agricultural Sciences* **80**, 679–685 (2010).

306. Saharawat, Y. S. *et al.* Evaluation of alternative tillage and crop establishment methods in a rice–wheat rotation in North Western IGP. *Field Crops Research* **116**, 260–267 (2010).

307. Sainju, U. M., Lenssen, A. W., Caesar-TonThat, T. & Evans, R. G. Dryland crop yields and soil organic matter as influenced by long-term tillage and cropping sequence. *Agronomy Journal* **101**, 243–251 (2009).

308. Sainju, U. M. & Singh, B. P. Tillage, cover crop, and kill-planting date effects on corn yield and soil nitrogen. *Agronomy Journal* **93**, 878–886 (2001).

309. Sainju, U. M., Whitehead, W. F., Singh, B. P. & Wang, S. Tillage, cover crops, and nitrogen fertilization effects on soil nitrogen and cotton and sorghum yields. *European Journal of Agronomy* **25**, 372–382 (2006).

310. Schillinger, W. F., Cook, R. J. & Papendick, R. I. Increased dryland cropping intensity with no-till barley. *Agronomy Journal* **91**, 744–752 (1999).

311. Schlegel, A. J., Dhuyvetter, K. C., Thompson, C. R. & Havlin, J. L. Agronomic and economic impacts of tillage and rotation on wheat and sorghum. *Journal of Production Agriculture* **12**, 629–636 (1999).

312. Schlegel, A. J., Assefa, Y., Haag, L. A., Thompson, C. R. & Stone, L. R. Long-term tillage on yield and water use of grain sorghum and winter wheat. *Agronomy Journal* **110**, 269–280 (2018).

313. Schwab, G. J., Whitney, D. A., Kilgore, G. L. & Sweeney, D. W. Tillage and phosphorus management effects on crop production in soils with phosphorus stratification. *Agronomy Journal* **98**, 430–435 (2006).

314. Selles, F., McConkey, B. G. & Campbell, C. A. Distribution and forms of P under cultivator- and zero-tillage for continuous- and fallow-wheat cropping systems in the semi-arid Canadian prairies. *Soil and Tillage Research* **51**, 47–59 (1999).

315. Sepat, S., Bana, R. S., Meena, S. L. & Rana, D. S. Assessment of conservation agriculture and intercropping practices for enhanced productivity and profitability in maize (Zea mays). *Indian Journal of Agricultural Sciences* **89**, 714–720 (2019).

316. Sessiz, A., Alp, A. & Gursoy, S. Conservation and conventional tillage methods on selected soil physical properties and corn (Zea Mays L.) yield and quality under croppin system in Turkey. *Bulgarian Journal of Agricultural Science* **16**, 597–608 (2010).

317. Sessiz, A., Sogut, T., Alp, A. & Esgici, R. Tillage effects on sunflower (helianthus annuus, l.) emergence, yield, quality, and fuel consumption in double cropping system. *Journal of Central European Agriculture* **9**, 697–710 (2008).

318. Shapiro, C. A. *et al.* Tillage and management alternatives for returning conservation reserve program land to crops. *Agronomy Journal* **93**, 850–862 (2001).

319. Sharma, P. K., de Datta, S. K. & Redulla, C. A. Response of maize (Zea mays L.) and mungbean (Vigna radiata L.) to tillage in relation to water table depth in tropical lowland rice soils. *Soil and Tillage Research* **12**, 65–79 (1988).

320. Sharma, R. K., Srinivasa Babu, K., Chhokar, R. S. & Sharma, A. K. Effect of tillage on termites, weed incidence and productivity of spring wheat in rice-wheat system of North Western Indian plains. *Crop Protection* **23**, 1049–1054 (2004).

321. Sharratt, B. . Barley yield and evapotranspiration governed by tillage practices in interior Alaska. *Soil and Tillage Research* **46**, 225–229 (1998).

322. Si, P. *et al.* Effect of no-tillage with straw mulch and conventional tillage on soil organic carbon pools in Northern China. *Archives of Agronomy and Soil Science* **64**, 398–408 (2018).

323. Sidhu, D. & Duiker, S. W. Soil compaction in conservation tillage: crop impacts. *Agronomy Journal* **98**, 1257–1264 (2006).

324. Silva, F. A. M., Naudin, K., Corbeels, M., Scopel, E. & Affholder, F. Impact of conservation agriculture on the agronomic and environmental performances of maize cropping under contrasting climatic conditions of the Brazilian Cerrado. *Field Crops Research* **230**, 72–83 (2019).

325. Silva, P., Garrido, M., Shertzer, G. & Acevedo, E. Amount of Rain Until Third Leaf Explain Differences in Irrigated Durum Wheat Yield Between a Conventional and No-Tillage System in a Long-Term Crop Rotation System in Mediterranean Environment. *International Journal of Plant Production* **13**, 339–346 (2019).

326. Simón, M. R. *et al.* Integrated foliar disease management to prevent yield loss in Argentinian wheat production. *Agronomy Journal* **103**, 1441–1451 (2011).

327. Singer, J. W. *et al.* Tillage and compost affect yield of corn, soybean, and wheat and soil fertility. *Agronomy Journal* **96**, 531–537 (2004).

328. Singer, J. W., Logsdon, S. D. & Meek, D. W. Tillage and compost effects on corn growth, nutrient accumulation, and grain yield. *Agronomy Journal* **99**, 80–87 (2007).

329. Singer, J. W., Logsdon, S. D. & Meek, D. W. Soybean growth and seed yield response to tillage and compost. *Agronomy Journal* **100**, 1039–1046 (2008).

330. Singh, V., Ram, S., Bhatnagar, A. & Savita, U. Effect of tillage methods on soil properties and productivity of quality protein maize (Zea mays)-wheat (Triticum aestivum) system. *Indian Journal of Agronomy* **56**, 83–87 (2011).

331. Singh, V. K. *et al.* Soil physical properties, yield trends and economics after five years of conservation agriculture based rice-maize system in north-western India. *Soil and Tillage Research* **155**, 133–148 (2016).

332. Sistani, K. R., Sikora, F. J. & Rasnake, M. Poultry litter and tillage influences on corn production and soil nutrients in a Kentucky silt loam soil. *Soil and Tillage Research* **98**, 130–139 (2008).

333. Sistani, K. R. *et al.* Nutrient source and tillage impact on corn grain yield and soil properties. *Soil Science* **175**, 593–600 (2010).

334. Sithole, N. J. & Magwaza, L. S. Long-term changes of soil chemical characteristics and maize yield in no-till conservation agriculture in a semi-arid environment of South Africa. *Soil and Tillage Research* **194**, 104317 (2019).

335. Smith, D. R., Hernandez-Ramirez, G., Armstrong, S. D., Bucholtz, D. L. & Stott, D. E. Fertilizer and tillage management impacts on non-carbon-dioxide greenhouse gas emissions. *Soil Science Society of America Journal* **75**, 1070–1082 (2011).

336. So, H. B., Grabski, A. & Desborough, P. The impact of 14 years of conventional and no-till cultivation on the physical properties and crop yields of a loam soil at Grafton NSW, Australia. *Soil and Tillage Research* **104**, 180–184 (2009).

337. Soane, B. D. & Ball, B. C. Review of management and conduct of long-term tillage studies with special reference to a 25-yr experiment on barley in Scotland. *Soil and Tillage Research* **45**, 17–37 (1998).

338. Song, K. *et al.* Effects of tillage and straw return on water-stable aggregates, carbon stabilization and crop yield in an estuarine alluvial soil. *Scientific Reports* **9**, 4586 (2019).

339. Soon, Y. K. & Arshad, M. A. Tillage, crop residue and crop sequence effects on nitrogen availability in a legume-based cropping system. *Canadian Journal of Soil Science* **84**, 421–430 (2004).

340. Soon, Y. K., Malhi, S. S., Lemke, R. L., Lupwayi, N. Z. & Grant, C. A. Effect of polymer-coated urea and tillage on the dynamics of available N and nitrous oxide emission from Gray Luvisols. *Nutrient Cycling in Agroecosystems* **90**, 267–279 (2011).

341. Sow, A. A., Hossner, L. R., Unger, P. W. & Stewart, B. A. Tillage and residue effects on root growth and yields of grain sorghum following wheat. *Soil and Tillage Research* **44**, 121–129 (1997).

342. Spargo, J. T., Cavigelli, M. A., Mirsky, S. B., Maul, J. E. & Meisinger, J. J. Mineralizable soil nitrogen and labile soil organic matter in diverse long-term cropping systems. *Nutrient Cycling in Agroecosystems* **90**, 253–266 (2011).

343. Stecker, J. A., Buchholz, D. D., Hanson, R. G., Wollenhaupt, N. C. & McVay, K. A. Tillage and rotation effects on corn yield response to fertilizer nitrogen on aqualf soils. *Agronomy Journal* **87**, 409–415 (1995).

344. Steward, P. R., Thierfelder, C., Dougill, A. J. & Ligowe, I. Conservation agriculture enhances resistance of maize to climate stress in a Malawian medium-term trial. *Agriculture, Ecosystems and Environment* **277**, 95–104 (2019).

345. Stinner, B. R., Odum, E. P. & Crossley, D. A. Nutrient uptake by vegetation in relation to other ecosystem processes in conventional tillage, no-tillage and old-field systems. *Agriculture, Ecosystems & Environment* **10**, 1–13 (1983).

346. Su, Z. *et al.* Effects of conservation tillage practices on winter wheat water-use efficiency and crop yield on the Loess Plateau, China. *Agricultural Water Management* **87**, 307–314 (2007).

347. Sulek, A., Wyzinska, M. & Cacak-Pietrzak, G. Impact of tillage on yield and quality traits of grains of spring wheat cultivars. *Engineering for Rural Development* **18**, 600–606 (2019).

348. Sun, L. *et al.* Reasonable fertilization improves the conservation tillage benefit for soil water use and yield of rain-fed winter wheat: A case study from the Loess Plateau, China. *Field Crops Research* **242**, (2019).

349. Sun, L. *et al.* Conservation agriculture based on crop rotation and tillage in the semi-arid Loess Plateau, China: Effects on crop yield and soil water use. *Agriculture, Ecosystems & Environment* **251**, 67–77 (2018).

350. Sun, M. *et al.* Long-term evaluation of tillage methods in fallow season for soil water storage, wheat yield and water use efficiency in semiarid southeast of the Loess Plateau. *Field Crops Research* **218**, 24–32 (2018).

351. Susha, V. S. *et al.* Impacts of tillage and herbicide mixture on weed interference, agronomic productivity and profitability of a maize – Wheat system in the North-western Indo-Gangetic Plains. *Field Crops Research* **219**, 180–191 (2018).

352. Taa, A., Tanner, D. & Bennie, A. T. P. Effects of stubble management, tillage and cropping sequence on wheat production in the south-eastern highlands of Ethiopia. *Soil and Tillage Research* **76**, 69–82 (2004).

353. Tabaglio, V. & Gavazzi, C. Monoculture maize (Zea mays L.) cropped under conventional tillage, no-tillage and N fertilization:(I) Three year yield performances. *Italian Journal of Agronomy* 61–68 (2009).

354. Tan, Y., Wu, D., Bol, R., Wu, W. & Meng, F. Conservation farming practices in winter wheat–summer maize cropping reduce GHG emissions and maintain high yields. *Agriculture, Ecosystems & Environment* **272**, 266–275 (2019).

355. Tarkalson, D. D., Hergert, G. W. & Cassman, K. G. Long-term effects of tillage on soil chemical properties and grain yields of a dryland winter wheat-sorghum/corn-fallow rotation in the great plains. *Agronomy Journal* **98**, 26–33 (2006).

356. Teal, R. K. *et al.* Effect of tillage and anhydrous ammonia application on nitrogen use efficiency of hard red winter wheat. *Journal of Sustainable Agriculture* **30**, 51–67 (2007).

357. Tessier, S., Peru, M., Dyck, F. B., Zentner, F. P. & Campbell, C. A. Conservation tillage for spring wheat production in semi-arid Saskatchewan. *Soil and Tillage Research* **18**, 73–89 (1990).

358. Therrien, M. C. & Grant, C. A. Effect of tillage management on yield performance in barley. *Canadian Journal of Plant Science* **78**, 301–303 (1998).

359. Thind, H. S., Sharma, S., Yadvinder Singh & Sidhu, H. S. Rice–wheat productivity and profitability with residue, tillage and green manure management. *Nutrient Cycling in Agroecosystems* **113**, 113–125 (2019).

360. Thomsen, I. K. & Sørensen, P. Tillage-induced N mineralization and N uptake in winter wheat on a coarse sandy loam. *Soil and Tillage Research* **89**, 58–69 (2006).

361. Tolon-Becerra, A., Tourn, M., Botta, G. F. & Lastra-Bravo, X. Effects of different tillage regimes on soil compaction, maize (Zea mays L.) seedling emergence and yields in the eastern Argentinean Pampas region. *Soil and Tillage Research* **117**, 184–190 (2011).

362. Tørresen, K. S., Skuterud, R., Weiseth, L., Tandsæther, H. J. & Haugan Jonsen, S. Plant protection in spring cereal production with reduced tillage. I. Grain yield and weed development. *Crop Protection* **18**, 595–603 (1999).

363. Tsegaye, T. & Hill, R. L. Wheel traffic placement effects on corn response under no-tillage and conventional tillage. *Journal of Production Agriculture* **9**, 95–101 (1996).

364. Tsuji, H., Yamamoto, H., Matsuo, K. & Usuki, K. The effects of long-term conservation tillage, crop residues and P fertilizer on soil conditions and responses of summer and winter crops on an Andosol in Japan. *Soil and Tillage Research* **89**, 167–176 (2006).

365. Tueche, J. R. & Hauser, S. Maize (Zea mays L.) yield and soil physical properties as affected by the previous plantain cropping systems, tillage and nitrogen application. *Soil and Tillage Research* **115–116**, 88–93 (2011).

366. Unger, P. W. Tillage and residue effects on wheat, sorghum, and sunflower grown in rotation. *Soil Science Society of America Journal* **48**, 885–891 (1984).

367. Varner, B. T., Epplin, F. M. & Strickland, G. L. Economics of no-till versus tilled dryland cotton, grain sorghum, and wheat. *Agronomy Journal* **103**, 1329–1338 (2011).

368. Varsa, E. C., Chong, S. K., Abolaji, J. O., Farquhar, D. A. & Olsen, F. J. Effect of deep tillage on soil physical characteristics and corn (Zea mays L.) root growth and production. *Soil and Tillage Research* **43**, 219–228 (1997).

369. Vazquez, L., Myhre, D. L., Gallaher, R. N., Hanlon, E. A. & Portier, K. M. Soil compaction associated with tillage treatments for soybean. *Soil and Tillage Research* **13**, 35–45 (1989).

370. Venterea, R. T., Maharjan, B. & Dolan, M. S. Fertilizer source and tillage effects on yield-scaled nitrous oxide emissions in a corn cropping system. *Journal of Environmental Quality* **40**, 1521–1531 (2011).

371. Verch, G., Kächele, H., Höltl, K., Richter, C. & Fuchs, C. Comparing the profitability of tillage methods in Northeast Germany-A field trial from 2002 to 2005. *Soil and Tillage Research* **104**, 16–21 (2009).

372. Verhulst, N. *et al.* Conservation agriculture for wheat-based cropping systems under gravity irrigation: Increasing resilience through improved soil quality. *Plant and Soil* **340**, 467–479 (2011).

373. Verhulst, N. *et al.* The effect of tillage, crop rotation and residue management on maize and wheat growth and development evaluated with an optical sensor. *Field Crops Research* **120**, 58–67 (2011).

374. Verhulst, N. *et al.* Soil water content, maize yield and its stability as affected by tillage and crop residue management in rainfed semi-arid highlands. *Plant and Soil* **344**, 73–85 (2011).

375. Videnović, Ž., Simić, M., Srdić, J. & Dumanović, Z. Long term effects of different soil tillage systems on maize (Zea mays L.) yields. *Plant, Soil and Environment* **57**, 186–192 (2011).

376. Vyn, T. J. & Raimbault, B. A. Evaluation of strip tillage systems for corn production in Ontario. *Soil and Tillage Research* **23**, 163–176 (1992).

377. Vyn, T. J., Galic, D. M. & Janovicek, K. J. Corn response to potassium placement in conservation tillage. *Soil and Tillage Research* **67**, 159–169 (2002).

378. Vyn, T. J. & Janovicek, K. J. Potassium placement and tillage system effects on corn response following long-term no till. *Agronomy Journal* **93**, 487–495 (2001).

379. Wagger, M. G. & Denton, H. P. Crop and tillage rotations: grain yield, residue cover, and soil water. *Soil Science Society of America Journal* **56**, 1233–1237 (1992).

380. Wang, H., Lemke, R., Goddard, T. & Sprout, C. Tillage and root heat stress in wheat in central Alberta. *Canadian Journal of Soil Science* **87**, 3–10 (2007).

381. Wang, S. *et al.* The influence of rotational tillage on soil water storage, water use efficiency and maize yield in semi-arid areas under varied rainfall conditions. *Agricultural Water Management* **203**, 376–384 (2018).

382. Wang, X. *et al.* Tillage time affects soil hydro-thermal properties, seedling growth and yield of maize (Zea mays L.). *Applied Ecology and Environmental Research* **16**, 6007–6023 (2018).

383. Wang, X. *et al.* Tillage and crop residue effects on rainfed wheat and maize production in northern China. *Field Crops Research* **132**, 106–116 (2012).

384. Watts, D. B. & Allen Torbert, H. Long-term tillage and poultry litter impacts on soybean and corn grain yield. *Agronomy Journal* **103**, 1479–1486 (2011).

385. West, T. D., Griffith, D. R., Steinhardt, G. C., Kladivko, E. J. & Parsons, S. D. Effect of tillage and rotation on agronomic performance of corn and soybean: Twenty-year study on dark silty clay loam soil. *Journal of Production Agriculture* **9**, 241–248 (1996).

386. West, T. D., Griffith, D. R. & Steinhardt, G. C. Effect of paraplowing on crop yields with no-till planting. *Journal of Production Agriculture* **9**, 233–237 (1996).

387. Whalen, J. K., Prasher, S. O. & Benslim, H. Monitoring corn and soybean agroecosystems after establishing no-tillage practices in Québec, Canada. *Canadian Journal of Plant Science* **87**, 841–849 (2007).

388. Wiatrak, P. J., Wright, D. L. & Marois, J. J. The impact of tillage and residual nitrogen on wheat. *Soil and Tillage Research* **91**, 150–156 (2006).

389. Wilhelm, W. W. *et al.* Dryland maize development and yield resulting from tillage and nitrogen fertilization practices. *Soil and Tillage Research* **10**, 167–179 (1987).

390. Wilhelm, W. W. & Wortmann, C. S. Tillage and rotation interactions for corn and soybean grain yield as affected by precipitation and air temperature. *Agronomy Journal* **96**, 425–432 (2004).

391. Winter, S. R. & Unger, P. W. Irrigated Wheat Grazing and Tillage Effects on Subsequent Dryland Grain Sorghum Production. *Agronomy Journal* **93**, 504–510 (2001).

392. Wolkowski, R. P. Row-placed fertilizer for maize grown with an in-row crop residue management system in southern Wisconsin. *Soil and Tillage Research* **54**, 55–62 (2000).

393. Wortmann, C. S., Drijber, R. A. & Franti, T. G. One-time tillage of no-till crop land five years post-tillage. *Agronomy Journal* **102**, 1302–1307 (2010).

394. Xu, J., Han, H., Ning, T., Li, Z. & Lal, R. Long-term effects of tillage and straw management on soil organic carbon, crop yield, and yield stability in a wheat-maize system. *Field Crops Research* **233**, 33–40 (2019).

395. Xu, Y. *et al.* Agronomic performance of late-season rice under different tillage, straw, and nitrogen management. *Field Crops Research* **115**, 79–84 (2010).

396. Xue, L. *et al.* Effects of tillage practices on water consumption and grain yield of dryland winter wheat under different precipitation distribution in the loess plateau of China. *Soil and Tillage Research* **191**, 66–74 (2019).

397. Yadav, G. S. *et al.* Soil carbon dynamics and productivity of rice–rice system under conservation tillage in submerged and unsubmerged ecologies of Eastern Indian Himalaya. *Carbon Management* **10**, 51–62 (2019).

398. Yadav, G. S. *et al.* Impact of no-till and mulching on soil carbon sequestration under rice (Oryza sativa L.)-rapeseed (Brassica campestris L. var. rapeseed) cropping system in hilly agro-ecosystem of the Eastern Himalayas, India. *Agriculture, Ecosystems & Environment* **275**, 81–92 (2019).

399. Yadav, G. S. *et al.* Conservation tillage and mulching effects on the adaptive capacity of direct-seeded upland rice (Oryza sativa L.) to alleviate weed and moisture stresses in the North Eastern Himalayan Region of India. *Archives of Agronomy and Soil Science* **64**, 1254–1267 (2018).

400. Yadav, G. S. *et al.* Conservation tillage and nutrient management effects on productivity and soil carbon sequestration under double cropping of rice in north eastern region of India. *Ecological Indicators* **105**, 303–315 (2019).

401. Yadav, G. S. *et al.* Effect of No-Till and Raised-Bed Planting on Soil Moisture Conservation and Productivity of Summer Maize (Zea mays) in Eastern Himalayas. *Agricultural Research* **7**, 300–310 (2018).

402. Yadvinder-Singh *et al.* Nitrogen and residue management effects on agronomic productivity and nitrogen use efficiency in rice-wheat system in Indian Punjab. *Nutrient Cycling in Agroecosystems* **84**, 141–154 (2009).

403. Yang, X. *et al.* Modelling the effects of conservation tillage on crop water productivity, soil water dynamics and evapotranspiration of a maize-winter wheat-soybean rotation system on the Loess Plateau of China using APSIM. *Agricultural Systems* **166**, 111–123 (2018).

404. Yang, Y. *et al.* Effects of tillage and mulching measures on soil moisture and temperature, photosynthetic characteristics and yield of winter wheat. *Agricultural Water Management* **201**, 299–308 (2018).

405. Yiridoe, E. K., Vyn, T. J., Weersink, A., Hooker, D. C. & Swanton, C. Farm-level profitability analysis of alternative tillage systems on clay soils. *Canadian Journal of Plant Science* **80**, 65–73 (2000).

406. Yoo, K. H., Touchton, J. T. & Walker, R. H. Runoff, sediment and nutrient losses from various tillage systems of cotton. *Soil and Tillage Research* **12**, 13–24 (1988).

407. Youjun, L. & Ming, H. Effects of tillage managements on soil rapidly available nutrient content and the yield of winter wheat in west Henan province, China. *Procedia Environmental Sciences* **11**, 843–849 (2011).

408. Zhang, X. *et al.* Tillage and residue management for long-term wheat-maize cropping in the North China Plain: I. Crop yield and integrated soil fertility index. *Field Crops Research* **221**, 157–165 (2018).

409. Zhang, Y. *et al.* Soil water use and crop yield increase under different long-term fertilization practices incorporated with two-year tillage rotations. *Agricultural Water Management* **221**, 362–370 (2019).

410. Zhang, Y. *et al.* The effects of rotating conservation tillage with conventional tillage on soil properties and grain yields in winter wheat-spring maize rotations. *Agricultural and Forest Meteorology* **263**, 107–117 (2018).

411. Zhang, Y. *et al.* Crop yield and soil properties of dryland winter wheat-spring maize rotation in response to 10-year fertilization and conservation tillage practices on the Loess Plateau. *Field Crops Research* **225**, 170–179 (2018).

412. Zhao, C. *et al.* No-tillage reduces competition and enhances compensatory growth of maize (Zea mays L.) intercropped with pea (Pisum sativum L.). *Field Crops Research* **243**, 107611 (2019).

413. Žugec, I. The effect of reduced soil tillage on maize (Zea mays L.) grain yield in Eastern Crotia (Yugoslavia). *Soil and Tillage Research* **7**, 19–28 (1986).

Reference used in the supplementary materials

1. Dimitriadis, S., Liparas, D. & ADNI. How random is the random forest? Random forest algorithm on the service of structural imaging biomarkers for Alzheimer's disease: from Alzheimer's disease neuroimaging initiative (ADNI) database. *Neural Regeneration Research* **13**, 962 (2018).

2. Fawcett, T. An introduction to ROC analysis. *Pattern Recognition Letters* **27**, 861–874 (2006).

3. NOAA/OAR/ESRL PSL. University of Delaware Air Temperature & Precipitation. https://www.esrl.noaa.gov/psd/data/gridded/data.UDel_AirT_Precip.html.

4. Martens, B. *et al.* GLEAM v3: satellite-based land evaporation and root-zone soil moisture. *Geoscientific Model Development* **10**, 1903–1925 (2017).

5. Miralles, D. G. *et al.* Global land-surface evaporation estimated from satellite-based observations. *Hydrology and Earth System Sciences* **15**, 453–469 (2011).

6. NOAA/OAR/ESRL PSL. CPC Global Daily Temperature. https://www.esrl.noaa.gov/psd/data/gridded/data.cpc.globaltemp.html (2020).

7. University of Tokyo. Soil Texture Map. http://hydro.iis.u-tokyo.ac.jp/~sujan/research/gswp3/soil-texture-map.html.

8. Sacks, W. J., Deryng, D., Foley, J. A. & Ramankutty, N. Crop planting dates: an analysis of global patterns. *Global Ecology and Biogeography* (2010).

9. University of Wisconsin-Madison. Crop Calendar Dataset: netCDF 5 degree. https://nelson.wisc.edu/sage/data-and-models/crop-calendar-dataset/netCDF0-5degree.php.

10. Portmann, F. T., Siebert, S. & Döll, P. MIRCA2000-Global monthly irrigated and rainfed crop areas around the year 2000: A new high-resolution data set for agricultural and hydrological modeling. *Global Biogeochemical Cycles* **24**, (2010).

11. Food and Agriculture Organization of the United Nations (FAO). *Save and grow: A policymaker's guide to the sustainable intensification of smallholder crop production.* http://www.fao.org/3/a-i2215e.pdf (2013).

12. Bryll, R., Gutierrez-Osuna, R. & Quek, F. Attribute bagging: improving accuracy of classifier ensembles by using random feature subsets. *Pattern Recognition* **36**, 1291–1302 (2003).

13. Nembrini, S., König, I. R. & Wright, M. N. The revival of the Gini importance? *Bioinformatics* **34**, 3711–3718 (2018).

14. Arguez, A. *et al.* NOAA's 1981–2010 U.S. Climate Normals: An Overview. *Bulletin of the American Meteorological Society* **93**, 1687–1697 (2012).
